# Outcomes of Delaying Parenteral Nutrition for 1 Week vs Initiation Within 24 Hours Among Undernourished Children in Pediatric Intensive Care: A Subanalysis of the PEPaNIC Randomized Clinical Trial

**DOI:** 10.1001/jamanetworkopen.2018.2668

**Published:** 2018-09-14

**Authors:** Esther van Puffelen, Jessie M. Hulst, Ilse Vanhorebeek, Karolijn Dulfer, Greet Van den Berghe, Sascha C. A. T. Verbruggen, Koen F. M. Joosten

**Affiliations:** 1 Department of Pediatrics and Pediatric Surgery, Intensive Care Unit, Erasmus MC-Sophia Children’s Hospital, University Medical Center Rotterdam, Rotterdam, the Netherlands; 2 Division of Pediatric Gastroenterology, Department of Pediatrics, Erasmus MC-Sophia Children’s Hospital, University Medical Center Rotterdam, Rotterdam, the Netherlands; 3 Division of Gastroenterology, Hepatology, and Nutrition, The Hospital for Sick Children, Toronto, Ontario, Canada; 4 Clinical Division and Laboratory of Intensive Care Medicine, Department of Cellular and Molecular Medicine, University Hospitals Katholieke Universiteit Leuven, Leuven, Belgium

## Abstract

**Importance:**

Undernourishment has been associated with poor outcomes of critical illness in children. The effects of withholding parenteral nutrition (PN) for 1 week in undernourished critically ill children are unknown.

**Objective:**

To assess the outcome effects of withholding PN for 1 week in undernourished critically ill children.

**Design, Setting, and Participants:**

This is a subanalysis of the randomized clinical trial Pediatric Early vs Late Parenteral Nutrition in Intensive Care Unit (PEPaNIC; N = 1440), which focused on the subgroup of pediatric intensive care unit (PICU) patients identified as undernourished on admission. Children included in the PEPaNIC trial were enrolled between June 18, 2012, and July 27, 2015. Undernourishment was defined as weight-for-age *z* score less than −2 in children younger than 1 year, and body mass index–for-age *z* score less than −2 in children 1 year or older. Data analysis was conducted from August 3, 2017, to July 6, 2018.

**Interventions:**

Patients were randomized to initiation of supplemental PN within 24 hours (early PN) or after 1 week (late PN) when enteral nutrition was insufficient.

**Main Outcomes and Measures:**

Primary end points were risk of new infections acquired in the PICU and time to live PICU discharge, assessed via multivariable logistic regression and Cox proportional hazard analyses, adjusted for risk factors.

**Results:**

A total of 289 of 1440 children (20.1%), term newborn to age 17 years, were identified as undernourished, of whom 150 of 717 patients (20.9%) were in the late PN group and 139 of 723 patients (19.2%) were in the early PN group. On admission, characteristics were similar among the treatment groups. Mean (SD) weight *z* scores were −3.33 (1.18) in the late PN group and −3.21 (1.09) in the early PN group. Compared with well-nourished PICU patients, undernourishment on admission was associated with lower likelihood of an earlier live PICU discharge (adjusted hazard ratio, 0.86; 95% CI, 0.75-0.99; *P* = .03). Among undernourished PICU patients, late PN reduced the risk of new infections by 11.0% (adjusted odds ratio, 0.39; 95% CI, 0.19-0.78; *P* = .01), and shortened the duration of PICU stay by a median of 2 days (earlier live PICU discharge: adjusted hazard ratio, 1.37; 95% CI, 1.06-1.75; *P* = .01). The safety outcomes mortality, incidence of hypoglycemia during the first week, and incidence of weight deterioration during PICU stay were similar between the treatment groups.

**Conclusions and Relevance:**

In undernourished critically ill children, withholding PN for 1 week was clinically superior to early PN.

**Trial Registration:**

ClinicalTrials.gov Identifier: NCT01536275

## Key Points

**Question:**

What are the effects of withholding parenteral nutrition in undernourished critically ill children?

**Findings:**

In a randomized clinical trial, compared with well-nourished pediatric intensive care unit patients, being acutely undernourished on admission to the pediatric intensive care unit was associated with prolonged intensive care dependency. In this subanalysis of 289 undernourished critically ill children with insufficient enteral nutritional intake, withholding supplemental parenteral nutrition for 1 week (late parenteral nutrition) reduced new infections and shortened intensive care dependency, as compared with early (<24 hours) supplemental parenteral nutrition.

**Meaning:**

Withholding parenteral nutrition during the first week of pediatric critical illness is clinically superior to early initiation in undernourished critically ill children.

## Teaser

This subanalysis of the Pediatric Early vs Late Parenteral Nutrition in Intensive Care Unit (PEPaNIC) randomized clinical trial assesses the outcome effects of withholding parenteral nutrition for 1 week vs initiation within 24 hours in undernourished critically ill children.

## Introduction

The prevalence of undernourishment in children on admission to the pediatric intensive care unit (PICU) has been shown to be up to 24%.^1^ Undernourishment on admission to the PICU has been associated with increased mortality and morbidity such as infectious complications, longer need for mechanical ventilation, and prolonged hospital stay.^2,3,4^ Observational cohort studies have shown that higher nutritional intake is associated with an improvement of nutritional status,^5,6,7,8^ although the role of parenteral nutrition (PN) herein has not been investigated.^9^ Assumptions have been made that an earlier and increased nutrition delivery might prevent deterioration of nutritional status and subsequently improve clinical outcome.^10^ This strategy is promoted more vigorously in undernourished patients, in whom macronutrient deficiency is presumed to be more detrimental during acute illness.^11^

Recently, the Pediatric Early vs Late Parenteral Nutrition in Intensive Care Unit (PEPaNIC) randomized clinical trial (RCT), including 1440 critically ill children, showed that withholding PN for 1 week (late PN) resulted in fewer new infections and reduced the duration of PICU stay as compared with initiating PN at day 1 (early PN).^12^ These clinical benefits were even larger in children who were at high risk of developing undernutrition, reflected by a high score on the Screening Tool for Risk on Nutritional Status and Growth (STRONGkids).^13^ However, withholding PN for 1 week in undernourished critically ill children unable to advance past low volumes of enteral nutrition (EN) raised concerns among experts.^11,14,15^ Recently updated guidelines advise to start supplemental PN earlier in undernourished children than in well-nourished children if EN intake is insufficient.^11,16^ This subanalysis of the PEPaNIC RCT investigated the effects of withholding supplemental PN in a subgroup of critically ill children who were acutely undernourished on admission to the PICU.

## Methods

### Patients and Procedure

These analyses were performed for children in the 3 PICUs (Belgium, the Netherlands, and Canada) who participated in the PEPaNIC RCT (recruitment from June 18, 2012, to July 27, 2015). This study has followed the Consolidated Standards of Reporting Trials (CONSORT) reporting guideline. The full study protocol has been reported previously and is available in Supplement 1.^12,17^ Briefly, 1440 critically ill children (term newborn to age 17 years) with a score on the STRONGkids of 2 or higher were included. This score ranges from 0 to 5, with a higher score indicating a higher risk of developing undernutrition. The children were randomly assigned to late PN (withholding PN during the first week) or early PN (initiation of PN at day 1) if EN was less than 80% of the target and was expected to be insufficient for at least 24 hours. Children in the late PN group received a mixture of dextrose, 5%, and saline to match the amount of fluid administered to those in the early PN group. After the first week, PN was also started in the late PN group if EN was less than 80% of the caloric target. Initiation and incline of EN were similar between the treatment groups.^12,17^ Both groups received parenteral micronutrients (vitamins, minerals, and trace elements) from day 2 onward if EN was less than 80% of the target.^12,17^ Furthermore, blood glucose control with insulin according to local targets was identical in both groups.^12,17^ In Leuven, Belgium, target range for blood glucose concentrations was 50 to 80 mg/dL in infants younger than 1 year and 70 to 100 mg/dL in older children (to convert blood glucose to millimoles per liter, multiply by 0.0555). In Rotterdam, the Netherlands, target range for blood glucose concentration was 72 to 144 mg/dL, except for patients with traumatic brain injury in which a range of 108 to 144 mg/dL was targeted. In Edmonton, Canada, insulin was administered to target blood glucose concentration less than 180 mg/dL. After every change in macronutrient intake or amount of administered insulin, blood glucose concentration was checked hourly, either within routine laboratory check or by use of bedside glucose meters, until 3 consecutive measurements were within the targeted range. If a central venous line was not or no longer in place for clinical purposes, any required PN was delivered via a peripheral line. Outcome assessors and investigators were not directly involved in the PICU and were blinded to the treatment allocation.

The institutional ethical review boards of the participating centers in Leuven, Belgium; Rotterdam, the Netherlands; and Edmonton, Canada, approved the study, which was performed in accordance with the Declaration of Helsinki and its amendments. Written informed consent was obtained from the parents or legal guardians.

For the current subanalysis, a subgroup of acutely undernourished children on admission was identified. The broad age range of the patients in our study population did not allow us to use the same definition in all children. Therefore, acute undernutrition was defined as weight-for-age *z* score less than −2 in children younger than 1 year and body mass index–for-age *z* score less than −2 in children 1 year or older.^18,19^ Severe acute undernutrition was defined as weight-for-age *z* score less than −3 in children 1 year or younger and body mass index–for-age *z* score less than −3 in children 1 year or older.^18,19^

### Outcomes

Primary outcomes were the incidence of new infections during the PICU stay and length of the PICU stay accounting for mortality as a competing risk.^17^ Discharge from PICU was defined as ready for discharge from PICU (ie, no longer need for, or at risk of, vital organ support).^17^ Secondary outcomes were 7-day mortality (ie, during the intervention window), death during PICU stay, death during hospital stay and 90-day mortality, incidence of hypoglycemia (blood glucose level <40 mg/dL) during the first week, incidence of weight *z* score deterioration during PICU stay (defined as a negative change in weight *z* score from admission to PICU discharge), duration of mechanical ventilatory support, and length of hospital stay.

### Statistical Analysis

The analyses were done based on intention to treat. Variables are reported as proportions, mean (SD) if normally distributed, or median (interquartile range) if not normally distributed. Proportions were analyzed univariably using χ^2^ test, means with *t* test, and medians with Mann-Whitney *U *test. Pediatric intensive care unit stay, hospital stay, and duration of mechanical ventilation were investigated univariably as the crude number of days and multivariably as the likelihood of earlier live PICU discharge, likelihood of earlier live hospital discharge, and likelihood of earlier live weaning from mechanical ventilation. The results on time to live PICU discharge, time to live hospital discharge, and time to live weaning from mechanical ventilation can potentially be biased by the rate of mortality. Therefore, these multivariable time-to-event effect sizes were calculated with the use of Cox proportional hazards analysis, with data of survivors censored at 90 days, and data of nonsurvivors set beyond all survivors at 91 days to account for mortality as competing risk. The multivariable analyses of dichotomized outcomes were performed using logistic regression. Odds ratios or hazard ratios (HRs) with 95% confidence intervals were calculated. Multivariable analyses were adjusted for the baseline risk factors center, age, diagnosis group, STRONGkids category,^13^ Pediatric Logistic Organ Dysfunction score,^20^ and Pediatric Index of Mortality 2 score.^21^

*P* values .05 or less were considered statistically significant and all tests were 2-sided. All analyses were performed with IBM SPSS Statistics, version 21 (IBM Corp). The *z* scores were calculated with the use of Growth Analyser Research Calculation Tool, version 4.^22^

## Results

### Patients Undernourished on PICU Admission

In total, 289 of 1440 children (20.1%) were acutely undernourished on admission, among whom 150 of 717 patients (20.9%) were assigned to the late PN group and 139 of 723 patients (19.2%) were assigned to the early PN group (Figure). The incidence of undernourishment on admission was similar in all centers: 21.3% in Leuven, Belgium; 19.5% in Rotterdam, the Netherlands; and 21.9% in Edmonton, Canada (*P* = .70). In total, 18.5% of the children with a medium risk score on the STRONGkids tool were undernourished vs 38.9% of the children with a high risk score (*P* < .001). Baseline characteristics for the undernourished children were similar for the late PN group and the early PN group (Table 1). The mean (SD) weight *z* score on PICU admission was −3.33 (1.18) in the late PN group and −3.21 (1.09) in the early PN group (Table 1). Enteral energy and macronutrient doses were similar in both treatment groups, whereas parenteral energy and macronutrient doses differed between the treatment groups, which showed adherence to the protocol (eMethods and eTable 1 in Supplement 2). At the time PN was initiated in the early PN group, more than 95% of critically ill children received less than 50% of caloric targets enterally.^23,24^ During the intervention period, 55 children (36.7%) in the late PN group and 43 children (30.9%) in the early PN group did not receive any EN (*P* = .30).

**Figure.**
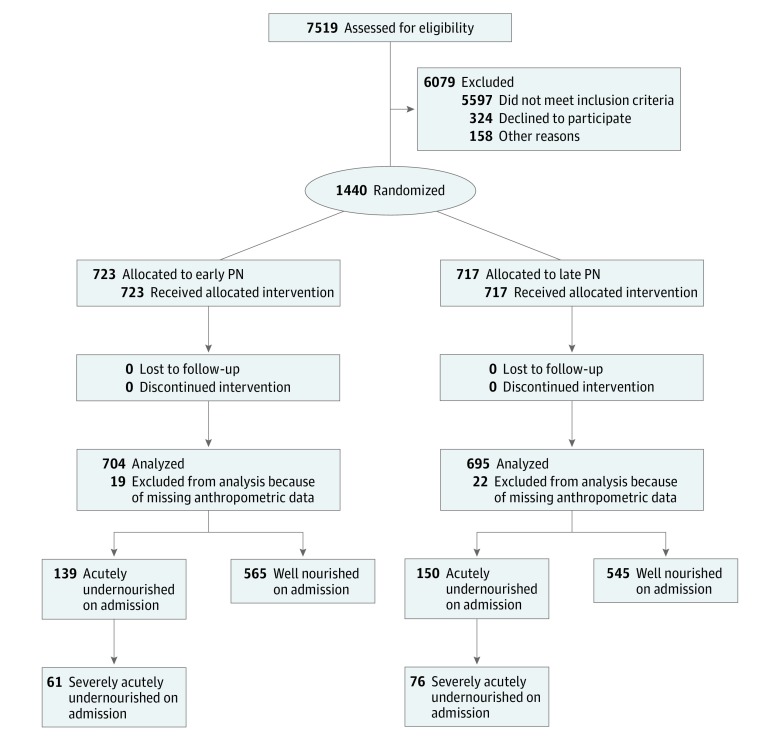
Flow Diagram of Children With and Without Undernourishment on Pediatric Intensive Care Unit Admission Acutely undernourished is defined as weight-for-age *z* score less than −2 (aged <1 year) or body mass index–for-age *z* score less than −2 (if aged ≥1).^18,19^ Severely acutely undernourished is defined as weight-for-age *z* score less than −3 (<1 year) or body mass index–for-age *z* score less than −3 (if aged ≥1 year).^18,19^ PN indicates parenteral nutrition.

**Table 1.** Baseline Characteristics of Children Undernourished on Admission

Characteristic	No. (%)		*P* Value
	Early PN (n = 139)	Late PN (n = 150)	
Male	88 (63.3)	85 (56.7)	.28
Age at randomization, median (IQR), y	0.43 (0.25-2.36)	0.46 (0.21-3.46)	.69
High STRONGkids category^a^	27 (19.4)	31 (20.7)	.88
Weight *z* score, mean (SD)^b^	−3.21 (1.09)	−3.33 (1.18)	.37
Severely undernourished on admission^c^	61 (43.9)	76 (50.7)	.25
PELOD score, median (IQR)^d^	21 (11-32)	21 (12-31)	.99
PIM2 score, mean (SD)^e^	−2.46 (1.52)	−2.47 (1.69)	.93
Risk of mortality, median (IQR), %^f^	6.3 (2.8-22.8)	6.7 (2.5-15.7)	.58
Diagnostic group			
Surgical			
Abdominal	7 (5.0)	10 (6.7)	.72
Burns	0	0	
Cardiac	58 (41.7)	66 (44.0)	
Neurologic	6 (4.3)	6 (4.0)	
Thoracic	3 (2.2)	2 (1.3)	
Transplant	0	2 (1.3)	
Trauma/orthopedic	8 (5.8)	9 (6.0)	
Other	5 (3.6)	1 (0.7)	
Medical			
Cardiac	6 (4.3)	6 (4.0)	
Gastrointestinal/hepatic	0	2 (1.3)	
Hematologic/oncologic	1 (0.7)	1 (0.7)	
Neurologic	11 (7.9)	9 (6.0)	
Renal	0	0	
Respiratory	29 (20.9)	28 (18.7)	
Other	5 (3.6)	8 (5.3)	
Syndrome or genetic abnormality			
No	96 (69.1)	94 (62.7)	.36
Yes	36 (25.9)	43 (28.7)	
Suspected	7 (5.0)	13 (8.7)	
Mechanical ventilatory support on PICU admission	124 (89.2)	127 (84.7)	.30
Inotrope or vasopressor medication on PICU admission	57 (41.0)	69 (46.0)	.41
Mechanical hemodynamic support on PICU admission	0	3 (2.0)	.25

Abbreviations: IQR, interquartile range; PN, parenteral nutrition; STRONGkids, Screening Tool for Risk on Nutritional Status and Growth; PELOD, Pediatric Logistic Organ Dysfunction; PICU, pediatric intensive care unit; PIM2, Pediatric Index of Mortality 2.

^a^ STRONGkids scores range from 0 to 5, with a score of 0 indicating low risk of malnutrition, 1 to 3 indicating medium risk, and 4 to 5 indicating high risk.^13^

^b^ Children younger than 1 year: weight-for-age *z* score; children 1 year or older: body mass index–for-age *z* score.^18,19^

^c^ Severe undernutrition defined as the following: for children younger than 1 year, weight-for-age *z* score less than −3; for children 1 year or older, body mass index–for-age z score less than −3.^18,19^

^d^ Scores range from 0 to 71, with higher scores indicate more severe illness.

^e^ Higher scores indicate a higher risk of mortality.

^f^ Based on PIM2 score = [exp^PIM2^/(1 + exp^PIM2^)] × 100.

### Undernourished vs Well-Nourished Children

Comparison of baseline characteristics between undernourished and well-nourished children showed that the group of undernourished children was younger, contained a higher proportion of respiratory diagnoses and lower proportion of neurosurgical diagnoses on PICU admission, and composed a lower proportion of children needing mechanical hemodynamic support (eTable 2 in Supplement 2). Being undernourished on admission was not associated with an increased risk of acquiring a new infection in the PICU, but was associated with both a prolonged duration of PICU stay and hospital stay with a median difference of 2 days and a lower likelihood of an earlier live PICU discharge (adjusted HR, 0.86; 95% CI, 0.75-0.99; *P* = .03), as well as a lower likelihood of an earlier live hospital discharge (adjusted HR, 0.83; 95% CI, 0.73-0.96; *P* = .01) (eTable 3 in Supplement 2). Undernourishment on admission was associated with a lower 7-day mortality, but a higher incidence of hypoglycemia during the first week as compared with well-nourished children. Death during PICU stay and hospital stay as well as 90-day mortality were not associated with undernourishment on admission (eTable 3 in Supplement 2). The baseline characteristics and outcomes of early PN vs late PN in well-nourished children are described in eTable 4 and eTable 5 in Supplement 2.

### Late PN vs Early PN in Children Undernourished on PICU Admission

In children who were undernourished on admission to the PICU, late PN reduced the risk of new infections by an absolute 11.0% (22.3% vs 11.3%; *P* = .02), with an adjusted odds ratio for new infections of 0.39 (95% CI, 0.19-0.78; *P* = .01). Late PN also shortened the duration of PICU dependency by a median of 2 days in undernourished children (6 vs 4 days; *P* = .01), with a higher likelihood of an earlier live PICU discharge (adjusted HR, 1.37; 95% CI, 1.06-1.75; *P* = .01) (Table 2). Safety outcomes mortality at all investigated time points and the incidence of hypoglycemia did not differ between late PN and early PN in undernourished children (Table 2).

**Table 2.** Outcomes of Late PN vs Early PN in Children Undernourished on Admission

Outcome	No. (%)		*P* Value	Adjusted OR or Adjusted HR (95% CI)^a^	*P* Value
	Early PN (n = 139)	Late PN (n = 150)			
Primary end points					
New infections	31 (22.3)	17 (11.3)	.02	0.39 (0.19-0.78)^b^	.01
Airway	16 (11.5)	8 (5.3)	.09		
Bloodstream	7 (5.0)	2 (1.3)	.09		
Urinary tract	1 (0.7)	0	.48		
Soft tissue	1 (0.7)	1 (0.7)	>.99		
No focus identified	4 (2.9)	4 (2.7)	>.99		
Other focus	2 (1.4)	2 (1.4)	.94		
Duration of PICU stay, median (IQR), d	6 (3-11)	4 (2-8)	.01	1.37 (1.06-1.75)^c^	.01
Secondary safety end points					
Death					
During first wk	1 (0.7)	1 (0.7)	>.99	0 (0 to >100)^b^	.35
During PICU stay	5 (3.6)	5 (3.3)	.90	0.70 (0.16-3.77)^b^	.75
During hospital stay	9 (6.5)	7 (4.7)	.50	0.58 (0.17-1.97)^b^	.39
90-d mortality	9 (6.5)	8 (5.3)	.80	0.74 (0.23-2.34)^b^	.60
Hypoglycemia (blood glucose <40 mg/dL) during first wk after randomization	12 (8.6)	20 (13.3)	.26	1.74 (0.75-4.06)^b^	.20
Deterioration of weight *z* score during PICU stay^e^	30 (57.7)	23 (47.9)	.33	0.60 (0.25-1.41)^b^	.24
Secondary efficacy end points, median (IQR), d					
Duration of mechanical ventilatory support	3 (2-7)	2.5 (1-5)	.10	1.39 (1.09-1.77)^c^	.01
Duration of hospital stay	14 (8-30)	10 (7-22)	.03	1.37 (1.07-1.75)^c^	.01

Abbreviations: HR, hazard ratio; IQR, interquartile range; OR, odds ratio; PICU, pediatric intensive care unit; PN, parenteral nutrition.

SI conversion factor: to convert blood glucose concentrations to millimoles per liter, multiply by 0.0555.

^a^ Adjusted for baseline risk factors center, age, diagnosis group, Pediatric Logistic Organ Dysfunction score, Pediatric Index of Mortality 2 score, and Screening Tool for Risk on Nutritional Status and Growth category.

^c^ Values are adjusted OR (95% CI).

^d^ Values are adjusted HR (95% CI).

^e^ Available in 100 children, 52 in the early PN group and 48 in the late PN group.

The duration of mechanical ventilatory support was shorter in the late PN group, with a higher likelihood of being weaned alive earlier from mechanical ventilation (adjusted HR, 1.39; 95% CI, 1.09-1.77; *P* = .01). Late PN also shortened the duration of hospital stay by a median of 4 days, with a higher likelihood of an earlier live discharge (adjusted HR, 1.37; 95% CI, 1.07-1.75; *P* = .01) (Table 2). In a subgroup of 100 undernourished critically ill children with weight *z* scores on admission and at discharge from the PICU available (48 in the late PN group and 52 in the early PN group), the incidence of weight *z* score deterioration was not different between the treatment groups (Table 2). A sensitivity analysis, assuming that all patients who died in the PICU had acquired a new infection during their PICU stay, supported our results; late PN reduced the risk of new infections by an absolute 9.7% (23.7% vs 14.0%; *P* = .03), with an adjusted odds ratio for new infections of 0.46 (95% CI, 0.24-0.91; *P* = .03).

### Late PN vs Early PN in Children Severely Undernourished on PICU Admission

In the late PN group, 76 of 717 children (10.6%) were severely undernourished; 61 of 723 children (8.4%) in the early PN group were severely undernourished (Figure). Among severely undernourished children, baseline characteristics were similar between the treatment groups (eTable 6 in Supplement 2). In severely undernourished children, late PN shortened the duration of PICU stay significantly with a median difference of 1 day, both in univariable and multivariable analyses corrected for baseline risk factors (Table 3). The percentage of severely undernourished children with a new infection was 10.5% in the group receiving late PN, as compared with 18.0% in the group receiving early PN, although this difference was not statistically significant. The safety outcomes were not significantly different between the treatment groups (Table 3).

**Table 3.** Outcomes of Late PN vs Early PN in Severely Undernourished Children^a^

Outcome	No. (%)		*P* Value	Adjusted OR or Adjusted HR (95% CI)^b^	*P* Value
	Early PN (n = 61)	Late PN (n = 76)			
Primary end points					
New infections	11 (18.0)	8 (10.5)	.21	0.33 (0.09-1.27)^c^	.11
Duration of PICU stay, median (IQR), d	5 (3-8)	4 (2-6)	.05	1.49 (1.04-2.13)^d^	.03
Secondary safety end points					
Death					
During first wk	0	1 (1.3)	.37	>100 (0.00-∞)^c^	>.99
During PICU stay	1 (1.6)	2 (2.6)	.69	0.05 (0->100)^c^	.60
During hospital stay	3 (4.9)	4 (5.3)	.93	0.40 (0.05-3.28)^c^	.39
90-d mortality	3 (4.9)	4 (5.3)	.93	0.25 (0.02-2.77)^c^	.26
Hypoglycemia (blood glucose <40 mg/dL) during first wk after randomization	6 (9.8)	9 (11.8)	.71	2.02 (0.39-10.41)^c^	.40
Weight *z* score deterioration	17 (63.0)	15 (55.6)	.58	0.69 (0.21-2.36)^c^	.56
Secondary efficacy end points, median (IQR), d					
Duration of mechanical ventilatory support	2 (2-7.5)	3 (1.25-5)	.30	1.43 (0.99-2.05)^d^	.06
Duration of hospital stay	15 (7.5-28)	10 (7-22)	.14	1.38 (0.96-2.00)^d^	.09

Abbreviations: HR, hazard ratio; IQR, interquartile range; OR, odds ratio; PICU, pediatric intensive care unit; PN, parenteral nutrition.

SI conversion factor: to convert blood glucose concentrations to millimoles per liter, multiply by 0.0555.

^a^ Severely undernourished was defined as weight-for-age *z *score less than −3 (if aged <1 year), or body mass index–for-age *z* score less than −3 (if aged ≥1 year).^18,19^

^b^ Adjusted for baseline risk factors center, age, diagnosis group, Pediatric Logistic Organ Dysfunction score, Pediatric Index of Mortality 2 score, and Screening Tool for Risk on Nutritional Status and Growth category.

^c^ Values are adjusted OR (95% CI).

^d^ Values are adjusted HR (95% CI).

## Discussion

Overall, we found that approximately 20% of the children in the PEPaNIC study were acutely undernourished on PICU admission and that these children performed worse with a lower likelihood of earlier live discharge from the PICU as well as from the hospital as compared with well-nourished children. The undernourished children benefited from withholding PN during the first week of critical illness as compared with initiating PN at the first day, as illustrated by a decreased risk of new infections, a shorter dependency on intensive care, and an accelerated live discharge from the hospital. The benefits of late PN were noticeable irrespective of center, age, disease severity, risk of mortality, diagnosis group, and STRONGkids score on admission. Late PN did not affect the safety outcomes mortality and incidence of hypoglycemia and was not associated with weight deterioration in the undernourished critically ill children.

The association between undernourishment and impaired clinical outcome, as in our study demonstrated by longer duration of PICU and hospital stay, has previously been described.^2,3,4^ However, baseline characteristics and diagnoses on admission in undernourished children differed from those in well-nourished children, which could have explained these differences in outcomes. Therefore, we cannot rule out that other factors played a role in the clinical outcome of children who are undernourished on admission.

The large proportion of undernourished children on admission to the PICU as well as the ongoing weight loss during PICU admission agree with previous studies.^5,25^ However, the beneficial effect of withholding PN during the first week of critical illness in these undernourished children contrasts with concerns raised by experts.^14,15,16^ The effect sizes of late PN vs early PN in the undernourished group were even higher than in the main trial cohort, which is in line with the larger effect size in critically ill children with a high STRONGkids score.^12^ In a small subgroup of severely undernourished children, late PN resulted in a significant higher likelihood of earlier live PICU discharge as compared with early PN. Although the proportions of new infections were in line with those found in the main trial cohort,^12^ the risk of acquiring a new infection was not statistically different between the randomization groups, probably owing to lack of power in this small subgroup. Although speculative, a possible explanation for these somewhat counterintuitive results of withholding PN in undernourished children, who are considered to be vulnerable for low nutritional intake, could be an attenuated immunosuppression. Undernourished children already have an altered immune system.^26^ Moreover, critical illness induces further immunosuppression,^27^ and early PN may potentially reduce immune function.^28,29,30^ An important function of the immune system is autophagy, an adaptive response to critical illness to control the cellular damage. In rabbits^31^ and critically ill adults,^32^ late PN enhanced autophagy as compared with early PN. Hence, possibly, undernourished critically ill children may have an immune response that differs from well-nourished critically ill children, making them even more susceptible for the benefits of withholding PN during the acute phase.

In contrast with the data from our randomized study, in nonrandomized observational cohort studies a lower nutritional intake, with or without PN, was associated with excessive weight deterioration.^5,6,7,8^ We cannot exclude that the different results between these observational studies and our study are related to the parenteral route of nutrition for which we randomized, although EN in our study was provided equally to both groups, in both timing of initiation as well as amounts. Nonetheless, we should consider the possibility that PN support during the acute phase of critical illness in children is not capable of influencing the children’s nutritional status assessed with anthropometric measurements. Hence, the deterioration of the nutritional status during acute critical illness appears primarily determined by the diagnosis and disease severity with which the child presents to the PICU and appears unaffected by PN support during the acute phase. The inflammatory response during critical illness possibly needs to be resolved before the child can transit into an anabolic state.^33^ Future research is warranted to determine when a patient transits from the acute phase to a stable or even recovery phase and whether and how in these phases PN support is able to improve the nutrititional status and (long-term) outcome of the patient.^34^

However, our findings are reassuring with respect to the concerns raised by experts about the consequence of late PN in undernourished critically ill children.^11,14,15^ Late PN was effective and did not negatively affect mortality, hypoglycemia, or change in weight *z* score as compared with early PN. Hence, there appears to be no support for early supplementation of PN during acute critical illness to improve outcome or to reverse or prevent weight deterioration in the PICU in undernourished critically ill children.

### Limitations

Our study had limitations. First, in children younger than 2 years with a history of being born prematurely, we were unable to use corrected ages to calculate weight-for-age and body mass index–for-age *z* scores. Consequently, the proportion of undernourished children may be overestimated, although such overestimation would be equal in both treatment groups owing to the randomized design. Second, weight measured in the PICU is highly influenced by factors such as fluid overload, tubes, and splints. Therefore, a change in weight during admission does not always reflect a change in lean body mass. Other measurements such as mid-upper arm circumference might be more reliable, as they are less affected by fluid change and extracorporeal items attached to the child. Despite these challenges to reliably measure the change in nutritional status, the inaccuracies in the anthropometric data will most likely be distributed equally in both treatment groups owing to the randomized design. Furthermore, the amount of administered fluid was similar in the 2 groups. Third, as longitudinal anthropometric measurements were available in only some of the undernourished children, there may be a selection bias. Fourth, we only presented short-term outcome measures. Follow-up of our patients, which is currently ongoing, has to point out the long-term effects of withholding PN.

## Conclusions

Critically ill children who are undernourished on PICU admission have a lower likelihood of an earlier live discharge from the PICU and the hospital as compared with well-nourished children. Withholding PN during the first week in these acutely undernourished critically ill children was clinically superior to supplementing PN early, with a lower risk of new infections and a higher likelihood of an earlier live discharge. Withholding PN during the first week was not associated with weight deterioration during PICU stay.

## Notes

Supplement 1. **Trial Protocol**

Supplement 2. **eMethods.** Protocol for Scoring of Infections **eTable 1.** Energy and Macronutrient Administration in Undernourished Children for the First Seven Days in PICU **eTable 2.** Baseline Characteristics of Undernourished vs Well-Nourished Children **eTable 3.** Main Outcomes of Undernourished vs Well-Nourished Children **eTable 4.** Baseline Characteristics of Children Well-Nourished on Admission in the Early-PN and Late-PN Group **eTable 5.** Outcomes of Late-PN vs Early-PN in Well-Nourished Children **eTable 6.** Baseline Characteristics of Children Severely Undernourished on Admission in the Early-PN and Late-PN Group **eReferences**
