# Supplementary material for: Outcomes of Delaying Parenteral Nutrition for 1 Week vs Initiation Within 24 Hours Among Undernourished Children in Pediatric Intensive Care: A Subanalysis of the PEPaNIC Randomized Clinical Trial
Source: JAMA Netw Open. 2018 Sep 14;1(5):e182668. doi: 10.1001/jamanetworkopen.2018.2668 (PMC6324499; doi:10.1001/jamanetworkopen.2018.2668)
Supplement: Supplement 1. — Trial Protocol [file jamanetwopen-1-e182668-s001.pdf]

This supplement contains the following items:

1. Original protocol, final protocol, summary of changes.

2. Final statistical analysis plan. *NB: only one version of the SAP was created and therefore no summary of changes has been included.*

Table of Contents

Original protocol PEPaNIC study…………………………………………………………….3

Final protocol PEPaNIC study……………………………………………………………….62

Summary of changes in protocol PEPaNIC study…………………………….................129

Final statistical analysis plan *NB: only one version of the SAP*

was created and therefore no summary of changes has been included……………….135

Original protocol PEPaNIC study

Paediatric Early versus late

Parenteral Nutrition In Critical illness

- PEPaNIC

(April 2012)

PROTOCOL TITLE '**Paediatric Early versus late Parenteral Nutrition In Critical illness**'

**Protocol ID**	**PEPaNIC - NL**
**Short title**	**PEPaNIC**
**Version**	**3**
**Date**	**22-04-2012**
**Coordinating investigator/project leader**	***Prof. Dr. G Van Den Berghe, Katholieke Universiteit, Leuven, Belgium***
**Principal investigator(s) (in Dutch: hoofdonderzoeker/uitvoerder)**<br>***Multicenter research: per site***	***Erasmus MC – Sophia Children's Hospital***<br>***Prof. Dr. D Tibboel***<br>***Dr. SCAT Verbruggen***<br>***Dr. KFM Joosten***<br>***Katholieke Universiteit, Leuven, Belgium***<br>***Prof. Dr. G Van Den Berghe***<br>***Prof. Dr. D. Mesotten***<br>***Prof. I Vanhorenbeek***<br>***Dr. L Desmet***<br>***Dr. T Fivez***
**Sponsor (in Dutch: verrichter/opdrachtgever)**	***Investigator Initiated***
**Independent physician(s)**	***Prof. Dr. IKM Reiss, Neonatology, Erasmus MC – Sophia Children's Hospital***
**Laboratory sites <*if applicable*>**	**Laboratory Paediatrics,**<br>**Erasmus MC –Sophia, Rotterdam**<br>**Laboratory Intensive Care Medicine, University Hospital Leuven, Belgium**
**Pharmacy <*if applicable*>**	***Erasmus MC – Sophia (Rotterdam site)***<br>***University Hospital Leuven (Leuven site)***

PROTOCOL SIGNATURE SHEET

Name	Signature	Date
For non-commercial research,<br><br>Head of Department:	Prof. Dr. D. Tibboel<br><br>Head of department, Pediatric ICU	
Coordinating Investigator/Project leader<br><br><br><br>Principal Investigator:	Dr. KFM Joosten<br><br><br>Dr. SCAT Verbruggen	

TABLE OF CONTENTS

1. INTRODUCTION AND RATIONALE 9

2. OBJECTIVES 11

3. STUDY DESIGN 13

4. STUDY POPULATION 16

4.1 Population Base 16

4.2 Inclusion criteria 16

4.3 Exclusion criteria 16

4.4 Sample size calculation 16

5. TREATMENT OF SUBJECTS 18

5.1 Investigational treatment 18

5.2 Use of co-intervention (if applicable) 23

5.3 Escape medication (if applicable) 23

6. INVESTIGATIONAL MEDICINAL PRODUCT 23

7. METHODS 24

7.1 Study parameters/endpoints 24

7.1.1 Main study parameter/endpoint 24

7.1.2 Secondary study parameters/endpoints 24

7.1.3 Other study parameters 29

7.2 Randomisation, blinding and treatment allocation 30

7.3 Study procedures30

7.4 Withdrawal of individual subjects 33

7.4.1 Specific criteria for withdrawal (if applicable) 33

7.5 Replacement of individual subjects after withdrawal 34

7.6 Follow-up of subjects withdrawn from treatment 34

7.7 Premature termination of the study 34

8. SAFETY REPORTING 35

8.1 Section 10 WMO event 37

8.2 Adverse and serious adverse events 37

8.2.1 Suspected unexpected serious adverse reactions (SUSAR) 38

8.2.2 Annual safety report 38

8.3 Follow-up of adverse events 39

8.4 Data Safety Monitoring Board (DSMB) 39

9. STATISTICAL ANALYSIS 40

9.1 Descriptive statistics 40

9.2 Univariate analysis 41

9.3 Multivariate analysis 41

9.4 Interim analysis 43

10. ETHICAL CONSIDERATIONS 44

10.1 Regulation statement 44

10.2 Recruitment and consent 44

10.3 Objection by minors or incapacitated subjects (if applicable) 44

10.4 Benefits and risks assessment, group relatedness 45

10.5 Compensation for injury 45

10.6 Incentives 45

11. ADMINISTRATIVE ASPECTS AND PUBLICATION 46

11.1 Handling and storage of data and documents 46

11.2 Amendments 46

11.3 Annual progress report 46

11.4 End of study report 46

11.5 Public disclosure and publication policy 47

12. REFERENCES

LIST OF ABBREVIATIONS AND RELEVANT DEFINITIONS

ABR	ABR form, General Assessment and Registration form, is the application form that is required for submission to the accredited Ethics Committee (In Dutch, ABR = Algemene Beoordeling en Registratie)
AE	Adverse Event
AR	Adverse Reaction
CA	Competent Authority
CCMO	Central Committee on Research Involving Human Subjects; in Dutch: Centrale Commissie Mensgebonden Onderzoek
CV	Curriculum Vitae
DSMB	Data Safety Monitoring Board
EU	European Union
EudraCT	European drug regulatory affairs Clinical Trials
GCP	Good Clinical Practice
IB	Investigator's Brochure
IC	Informed Consent
IMP	Investigational Medicinal Product
IMPD	Investigational Medicinal Product Dossier
METC	Medical research ethics committee (MREC); in Dutch: medisch ethische toetsing commissie (METC)
(S)AE	(Serious) Adverse Event
SPC	Summary of Product Characteristics (in Dutch: officiële productinfomatie IB1-tekst)
Sponsor	The sponsor is the party that commissions the organisation or performance of the research, for example a pharmaceutical<br><br>company, academic hospital, scientific organisation or investigator. A party that provides

funding for a study but does not commission it is not regarded as the sponsor, but referred to as a subsidising party.

SUSAR Suspected Unexpected Serious Adverse Reaction

Wbp Personal Data Protection Act (in Dutch: Wet Bescherming Persoonsgevens)

WMO Medical Research Involving Human Subjects Act (in Dutch: Wet Medisch-wetenschappelijk Onderzoek met Mensen

SUMMARY

Rationale: It has recently been shown in a large randomized study in adult ICU patients that the early PN caused an increase of morbidity (infections and increased length of stay on the ICU). Such a study is warranted also in critically ill children of different age groups, as PN has been associated with increased incidence of complications, such as (line) infections, hyperglycemia and hepatic steatosis. It has not been studied whether early PN, when enteral feeding is insufficient, influences the outcome of critically ill children.

Objective: The ultimate aim of this project is to answer the question whether insufficient EN should or should not be supplemented with PN early in the disease course of critical illness in children. The scientific objectives of the study are the following: 1) Performing the first well-designed and sufficiently powered, multicentre RCT to test the effects of early PN supplementation on the health of critically ill children. 2)) Performing an economic evaluation study, investigating the costs and cost-effectiveness, during hospitalisation of early versus late PN supplementation. 3) Starting to unravel potential mechanisms that underlie any difference in acute and long term outcome with early or late PN supplementation by studying metabolic, endocrine, inflammatory and (epi.)genetic markers.

Study design: This study is a large, two-centre, non-blinded, randomized, controlled study.

Consecutive patients are randomly assigned to one of the two treatment study groups using a digital system with central, computerised randomisation. Randomisation – in a one to one allocation ratio - is performed per centre and using permuted blocks of 10 per diagnostic stratum. The block size remains unknown to bed-side physicians and nurses, responsible for patient recruitment and therapy assignment.

Study population: Critically ill children (0 – 18 yrs), with nutritional risk score (STRONGkids® see appendix) 2 or more, who are not able to take oral nutrition and are expected to stay in PICU for more than 24h are eligible for inclusion. Exclusion criteria are a “do not resuscitate” code at the time of PICU admission, expected death within 12 hours, re-admission to the PICU after previously being randomised to the PEPaNIC trial, transfer from another PICU after a stay of more than seven days, ketoacidotic or hyperosmolar coma on admission or inborn metabolic diseases requiring specific diet, premature newborns (<37 weeks gestational age), patients on Total Parenteral Nutrition for >7 days prior to inclusion, short bowel syndrome or other conditions which required home-PN.

Intervention: The “early PN” strategy will be the nutritional management currently applied in the participating centres, acting as “control”. The “late PN” strategy comprises initiation of this strategy only after day 7 in PICU, patients will receive a mixture of Glucose 5% and NaCl 0.9% at, respectively, 60% and 40% of the total flow rate that is required to obtain standard optimal hydration taking into account the volume of EN that is being delivered. If enteral feeding of at least 80% of the calculated calories is not possible after 7 days in ICU, PN, as specified above, is initiated on day 8.

Main study parameters/endpoints: We will compare “early PN” with “late PN” in paediatric ICU patients at risk of developing malnutrition in the ICU. The primary focus of the PEPaNIC study is clinical outcome, more specifically the acquisition of new ICU infections, the dependency on intensive medical care and convalescence from critical illness.

Nature and extent of the burden and risks associated with participation, benefit and group relatedness: The burden is expected to be minimal as it will only entail additional blood draws, which will be taken from clinical lines or in addition to pricks for clinical purpose. The long-term follow up will be held as part of already organised follow-up outdoor clinics and which are developed to help children and their parents to recover physically, emotionally and socially after ICU admissions. For mechanistic and exploratory studies, muscle strength testing and an ultrasound evaluation of the skeletal muscle and adipose tissue compartments will be performed. Furthermore, as far as practically feasible, every patient is approached just prior to hospital discharge, to complete a validated, semi-structured child health questionnaire (Functional Independence Measure (WEEFIM), Health Utilities Index (HUI) and Child Health Questionnaire (CHQ)).

The risk in participating to the study and being randomized to the "late PN' group are negligible, and specifically compass an increased risk of developing hypoglycemia and/or underfeeding. However, safety measures will be taken to further decrease these risks.

GROUP relatedness: This study was already performed in adult ICU patients. However, the results of this study should NOT be translated one-on-one to the pediatric patient, as critically ill children of different age groups have different metabolic and nutritional issues. Therefore, this study deserves to be repeated in our population of critically ill children.

1. INTRODUCTION AND RATIONALE

It has recently been shown that withholding parenteral nutrition (PN.) during the first week of critical illness (Late PN) is beneficial in comparison with the early supplementation of insufficient enteral nutrition (EN) with PN (Early PN) in critically ill adults (1). The benefits of Late PN mainly encompassed a decrease in the incidence of new infections in the ICU, a shortening of the stay in the ICU and hospital, and a reduction in healthcare costs. These findings put pressure on the current guidelines by the European Society of Parenteral & Enteral Nutrition (ESPEN.), which recommend the practice of Early PN in critically adults (2). These current medical practice and expert panels advocate the early supplementation by PN. This is based on the premise that PN is probably beneficial, and if not, harmless. This contrasts strongly with the findings of the study in adult critically ill patients where it has been shown that this early supplementation by PN is not providing benefit and is actually causing harm to patients by increasing the morbidity and delaying recovery.

The detrimental impact of the practice of Early PN in critically ill adults has alerted the clinical community that also the guidelines for nutritional strategy in paediatric critically ill patients are merely based on expert opinion. Not only is Early PN current practice in most paediatric ICUs, it is also often more aggressively promoted (3, 4). This is further evidenced by the presence of national governmental healthcare programs that include the success rate for reaching nutritional goals as a quality benchmark (5). Therefore critically ill children may be exposed to risk by the aggressive feeding protocols, which were previously deemed innocent or even beneficial. We and others have shown recently that current aggressive feeding protocols with early high protein and lipid intakes, not only fail to achieve anabolism, but have potential negative side effects, such as increased risk of developing hyperglycemia, dyslipidemia and insulin resistance (6, 7). Furthermore, there is currently no evidence of beneficence of early supplementation of parenteral nutrition, although this is practiced in multiple PICU's according current guidelines. PN has been associated with increased incidence of complications, such as (line) infections, hyperglycemia and hepatic steatosis. Furthermore, it is well recognized that the quality of current PN solutions lack sufficient scientific basis and are potentially harmful (8). Therefore, it is unclear whether an early supplementary intervention of parenteral nutrition to try and decrease catabolism in critically ill children outweighs the potential harm of such therapy. Furthermore, recent studies have suggested a negative role of early aggressive nutrition in the interference of autophagy of cellular damage in critically ill patients, a process that may protect against infectious diseases and hyperinflammation (9-11). Children are in the early phase of their life when the fundaments of metabolism are developing. Therefore, it cannot be excluded that the aggressive nutritional support given to critically ill children has repercussions on the long run, until later in their adult life. And thus, such a study deserves to be performed in a population of critically ill children.

In our study we aim to evaluate whether the current practice of Early PN in critically ill children really provides clinical benefits over a strategy of withholding PN during the first 7 days in the paediatric ICU. The evaluation will be done in a multicentre randomized controlled trial performed in two large expert tertiary referral paediatric ICUs. The study will be statistically sufficiently powered to detect differences in clinically relevant outcome variables (the rate of new infections and the length of stay in the ICU). The trial will also be able to detect a doubling or halving

of the mortality rate with a respectable statistical power (details on statistical power are given in the description further on). The results of this randomized controlled trial will provide, for the first time, high quality evidence for practice guidelines for nutrition in critically ill children. It will answer the question whether Early PN is beneficial, harmful or neutral in comparison with the new therapy of withholding PN during the first week of critical illness. In weighing this evidence for current nutritional practice, this study will take into account 1) the short-term as well as the long-term health effects of the feeding protocols, 2) costs, and cost-effectiveness and 3) some insights into the underlying mechanisms of the observed clinical differences.

2. OBJECTIVES

The ultimate aim of this project is to answer the question whether insufficient EN should or should not be supplemented with PN early in the disease course of critical illness in children.

The scientific objectives of the study are divided into 3 main work-packages (WP 1-3) and are the following:

- WP1: Performing and completing the first well-designed and sufficiently powered, multicentre RCT to test the effects of early PN supplementation on the health of critically ill children. The short-term outcome analysis, as well as a substantial part of the long-term follow-up, will be completed within the time frame of the study.
- WP2: an economic evaluation, in which costs and cost-effectiveness will be investigated of early versus late PN supplementation during hospitalization.
- WP3: Starting to unravel potential mechanisms that underlie any difference in acute and long term outcome with early or late PN supplementation by studying metabolic, endocrine, inflammatory and (epi.)genetic markers in the blood of critically ill children included in the study.

Primary Objective:

The primary focus of the PEPaNIC study is clinical outcome, more specifically the acquisition of new ICU infections, the dependency on intensive medical care and convalescence from critical illness, this is part of WP1.

The primary efficacy endpoints for this RCT are the incidence of new infections and the time to discharge alive from ICU. We expect to see a decrease in secondary infections from 20% to 15%, which is based on a previous study in critically ill children in Leuven (12) and on retrospective data in critically ill children who were admitted for at least four days in the PICU, Rotterdam.

Number of patients with new infections and types of infection will be assessed by numbers and percentages, and the duration of any antibiotics therapy initiated after randomisation for those patients requiring antibiotics will be analysed by non-parametrical tests. As the time of ICU discharge to the regular ward may be affected by the availability of beds on the regular wards, which could induce bias, we a priori decided to analyse "time to discharge from ICU" as "time to ready for discharge from ICU". A patient is considered "ready for discharge" as soon as all clinical conditions for ICU discharge have been fulfilled (no longer in need for vital organ support). Time to discharge alive from ICU will be reported by Kaplan-Meier plots, with ICU non-survivors censored beyond the longest ICU stay of survivors and censoring time of patients still in the ICU at closing of the data file (90 days after last patient inclusion) over both treatment groups. The impact of "late PN" versus "early PN" will be analysed, with and without correction for age, nutritional status and risk categories and type and severity of illness, by Cox proportional hazard analysis. The distribution of the actual time to discharge from ICU will be

reported for ICU-survivors and ICU-non-survivors separately. In view of the time window of the randomised intervention in ICU, also the proportion of patients staying beyond 8 days in ICU will be reported. Analyses on blood and urine for the primary clinical analyses include routine chemistry, haematology, and markers of inflammation. Further epigenetic, metabolic, endocrine and inflammatory measurements on stored samples in the context of mechanistic analyses will be planned. All new infections of the lungs, the blood stream, the urinary tract and wounds are recorded by an infectious disease specialist. A nosocomial infection is defined as a localized or systemic condition 1) that results from adverse reaction to the presence of an infectious agent(s) or its toxin(s) and 2) that was not present or incubating at the time of admission to the PICU. The information used to determine the presence and classification of an infection should be a combination of clinical findings and results of laboratory and other tests. Definitions various types of proven and suspected infections will be used according to clinical, laboratory evidence and/or supportive data. WHO definitions for nosocomial infections will be used (13).

TABLE I. **Simplified criteria for surveillance of nosocomial infections**

Type of nosocomial infection	Simplified criteria
Surgical site infection	Any purulent discharge, abscess, or spreading cellulitis at the surgical site during the month after the operation
Urinary infection	Positive urine culture (1 or 2 species) with at least 10^5 bacteria/ml, with or without clinical symptoms
Respiratory infection	Respiratory symptoms with at least two of the following signs appearing during hospitalization:<br>— cough<br>— purulent sputum<br>— new infiltrate on chest radiograph consistent with infection
Vascular catheter infection	Inflammation, lymphangitis or purulent discharge at the insertion site of the catheter
Septicaemia	Fever or rigours and at least one positive blood culture

WHO definitions for nosocomial infections (13).

Clinical evidence is derived from direct observation of the infection site or review of other pertinent sources of data, such as the patient's chart. Laboratory evidence includes results of cultures, antigen or antibody detection tests, or microscopic visualization. Supportive data are derived from other diagnostic studies, such as x-ray,

ultrasound, computed tomography (CT) scan, magnetic resonance imaging (MRI), radiolabel scan, endoscopic procedure, biopsy, or needle aspiration. It will be recorded during the entire ICU period until discharge, death or until day 90 of admittance.

Bacteraemia is further classified by responsible pathogen and as catheter-related blood stream infection versus other bacteraemia.

Secondary Objectives:

WP2. Performing an economic evaluation study, investigating the effects of early versus late PN supplementation during hospitalization.

WP3. Starting to unravel potential mechanisms that underlie any difference in acute and long-term outcome with early or late PN supplementation by studying metabolic, endocrine, inflammatory and (epi.)genetic markers in the blood of critically ill children included in the study.

3. STUDY DESIGN

This study is a large, two-centre, non-blinded, randomized, controlled study. The study intervention will last for a maximum of 7 days per patient, and the entire study (final patient completed study) is anticipated to last 4 years, excluding the long-term follow-up, the health-economic analysis and the completion of the mechanistic studies. (See figure)

Consecutive patients are randomly assigned to one of the two treatment study groups using a digital system with central, computerised randomisation. Randomisation – in a one to one allocation ratio - is performed per centre and using permuted blocks of 10 per diagnostic stratum. The block size remains unknown to bed-side physicians and nurses, responsible for patient recruitment and therapy assignment.

The "early PN" strategy will be the nutritional management currently applied in the participating centres, acting as "control".

The "late PN" strategy comprises initiation of this strategy only after day 7 in PICU.

Timeline of the entire study

Work package 1 (WP1)

The first milestone (M1) of the study will be the start of the large RCT in the centre of the

coordinating investigator/project leader in Leuven, closely followed by our center at Erasmus MC-Sophia. The study will continue in parallel in both centers until patient number 720 will have left the ICU. At that time point, a safety interim analysis will be performed (milestone M2). Three possible scenarios are foreseen depending on the result of this analysis:

1. The interim analysis shows harm by "Late PN" and the clinical study will be prematurely stopped ("no go" decision).

2. The data safety monitoring board requests a new safety interim analysis because of reasonable doubt about the safety of "Late PN". In that case, the study will continue in both centers under very strict monitoring, and planning of additional safety interim analyses if necessary, until the end of the study (either when completed or when prematurely stopped).

3. The study will be continued in both centers as planned, without further interim analyses, until all 1440 patients have been included.

In all three scenarios the end of the study will be milestone 3 (M3). Together with the finalization and cross-check of the clinical database and the writing of the corresponding manuscript of the short-term clinical outcome, this will constitute deliverable 1 (D1). Hence, D1 is guaranteed irrespective of premature interruption of the study. In view of the outcome results of the adult EPaNIC study, such premature interruption will rather be unlikely. The applicants have extensive experience in the management of large RCTs, including in critically ill children, and handling and monitoring of the

corresponding large clinical databases. This invaluable experience will be key to successful accomplishment of this ambitious study.

Work package 2 (WP2)

The fourth milestone (M4) will be targeted for before the end of the clinical study and consists of the setup of appropriate template database structures for import of the data that will be needed for the health economy analysis. This will allow an efficient economic evaluation study shortly after completion of the clinical study and is planned within the 4 year time frame and of the study (Milestone M5 and deliverable D2).

In the unlikely event of premature interruption of the study, the health economic analysis will be performed after the safety interim analysis on which the decision to stop was based. Previous experience in detailed analyses of healthcare resource utilization in large randomized clinical studies, as well as close collaboration with the Finance & Accounting department and the pharmacies of both participating hospitals will guarantee successful and timely accomplishment of this deliverable.

Work package 3

The mechanistic studies will be coordinated by and performed in the Leuven Laboratory of Intensive Care Medicine and in the Erasmus MC. Part of the mechanistic analyses will be systematically performed throughout the study. More specifically, leukocyte function tests require the fresh isolation of leukocytes and hence will be performed in parallel with work package 1. Thus, these analyses will be completed at the end of the clinical study (milestone M6 and deliverable D3). The samples needed for the other mechanistic studies will be taken throughout the clinical study and as deliverable 4 (D4) will be available together with M3 and D1. The other mechanistic studies involving the investigation of autophagy, genetics and cytokines in relation to the inflammatory response, as well as of the neuroendocrine axes will be planned and started in the last phase after completion of the clinical study.

In (the unlikely) case the study would be terminated prematurely, the mechanistic studies will be even more important, as the clinical community will ask for due explanations for harm caused during the study. Therefore, in any case they will be started shortly after all clinical data have been collected.

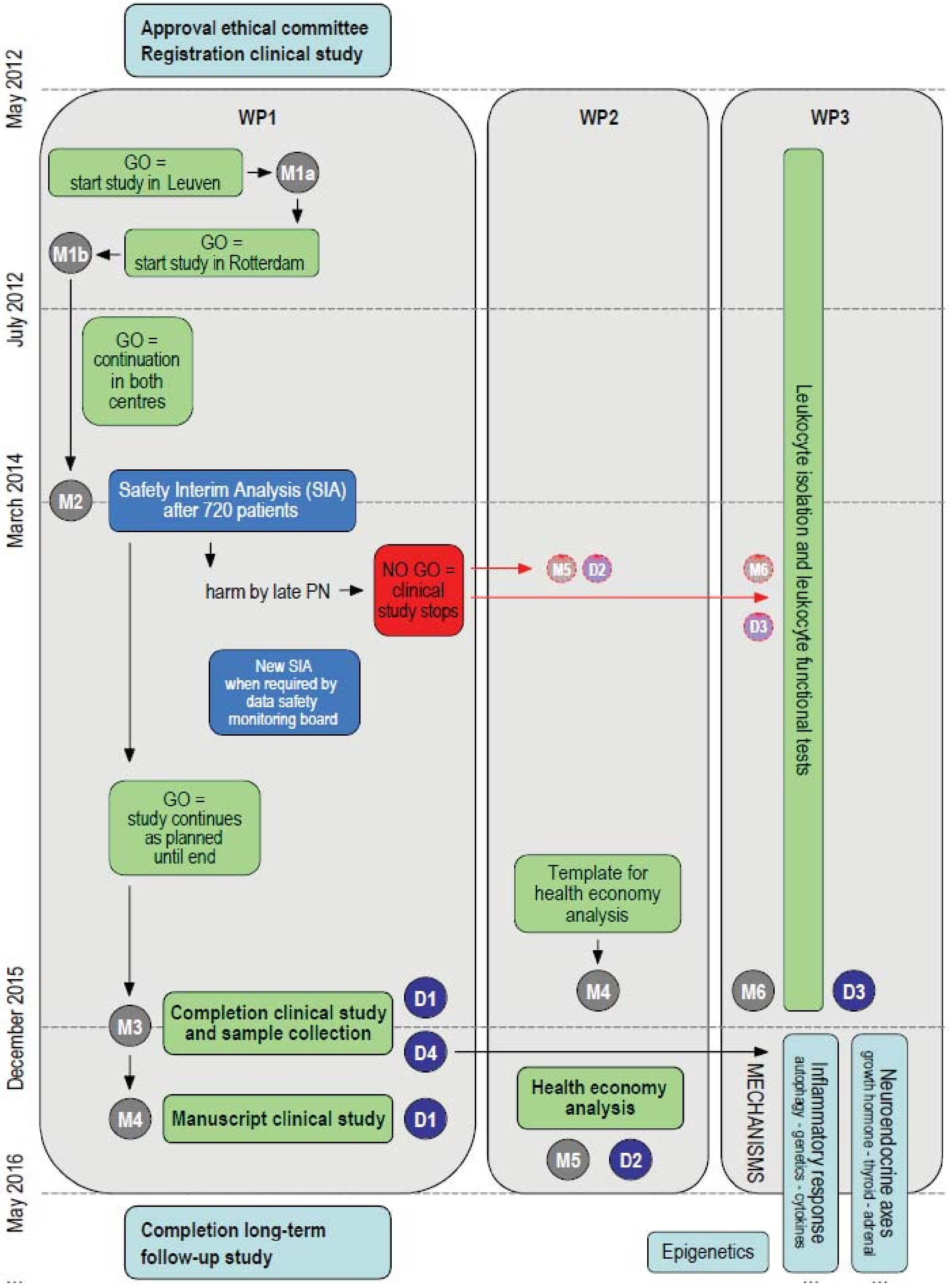

Approval ethical committee
Registration clinical study
May 2012
July 2012
March 2014
December 2015
May 2016
WP1
WP2
WP3
GO = start study in Leuven
M1a
GO = start study in Rotterdam
M1b
GO = continuation in both centres
M2
Safety Interim Analysis (SIA) after 720 patients
harm by late PN
NO GO = clinical study stops
M5
D2
M6
D3
New SIA when required by data safety monitoring board
GO = study continues as planned until end
Template for health economy analysis
M4
Leukocyte isolation and leukocyte functional tests
M6
D3
M3
Completion clinical study and sample collection
D1
D4
M4
Manuscript clinical study
D1
Health economy analysis
M5
D2
MECHANISMS
Inflammatory response
autophagy - genetics - cytokines
Neuroendocrine axes
growth hormone - thyroid - adrenal
Completion long-term follow-up study
Epigenetics

4. STUDY POPULATION

4.1 Population Base

The patient group of critically ill children (0 – 18 yrs), represents a significant proportion of the ICU population. In 2010, the 10-bed Leuven PICU admitted 527 critically ill children, the majority requiring artificial ventilation. 61% of the cases were admissions after cardiac surgery. Individual critically ill children had a median ICU stay of 3 days (inter-quartile range of 2-6 days) (mean 5.5 days). In the 35-bed PICU of the University of Rotterdam 1700 critically ill children were admitted in 2010 of which 600 (35.3%) were ventilated. Patients stayed in the PICU for a median of 2 days (mean 7.8 days). We anticipate an inclusion rate of 200-250 patients per centre per year.

4.2 Inclusion criteria

All critically ill children admitted to the participating PICUs are evaluated for nutritional risk and eligibility for inclusion in this study. All critically ill children, with nutritional risk score (STRONGkids® see appendix) 2 or more, who are not able to take oral nutrition and are expected to stay in PICU for more than 24h are eligible for inclusion.

4.3 Exclusion criteria

Exclusion criteria are a "do not resuscitate" code at the time of PICU admission, expected death within 12 hours, re-admission to the PICU after previously being participating in the PEPaNIC trial (except when < 48 hours after the initial discharge and still in the intervention window of the first 7 days), transfer from another PICU after a stay of more than seven days, ketoacidotic or hyperosmolar coma on admission or inborn metabolic diseases requiring specific diet, premature newborns (<37 weeks gestational age), patients on Total Parenteral Nutrition for >7 days prior to inclusion, short bowel syndrome or other conditions which required home-PN.

4.4 Sample size calculation

The sample size (N=1440, 720 per arm) is calculated in order to detect, with at least 80% power (one-tailed; the two-tailed power is 70%) and 95% certainty, a reduction in PICU infections from 20% to 15% and, with at least 90% power (two-tailed) and 95% certainty, a reduction in mean duration of stay in PICU of 1 day. With this sample size, and for safety reasons, also any substantial impact on mortality (increase or decrease with an absolute +/- 2%, although unexpected taken the adult data), can be excluded with a power of around 62-75% depending on the true mortality in the total population. We plan to calculate the true power of the study for detection of any eventual smaller differences in these outcomes.

5. TREATMENT OF SUBJECTS

5.1 Investigational treatment

The treatment, or intervention, in our study is best described as withholding PN in the early course of disease to reach currently recommended energy and protein intakes, when enteral delivery of these intakes is insufficient. In both allocation groups of the study, patients will be evaluated on a day to day basis to see which amount of enteral intake can be provided. The "early PN" group will receive current standard nutritional practice, which prescribes that PN should be added to EN as soon as possible in the course of disease, to achieve energy and protein goals. In the "late PN" group, we will wait to add PN, when EN is insufficient, until day 7.

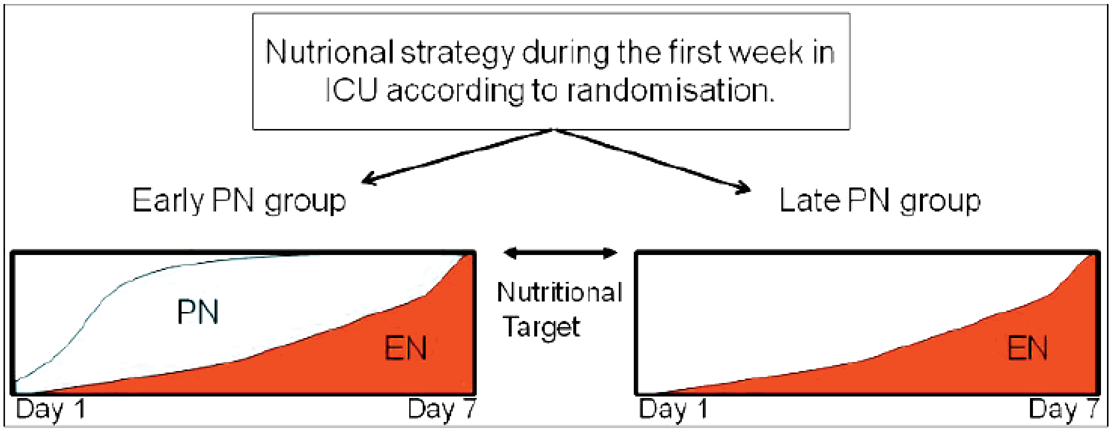

"Late PN", the so-called "intervention-group".

Both in Leuven and Rotterdam, patients randomised to the "late PN" group will receive a mixture of Glucose 5% and NaCl 0.9% at, respectively, 60% and 40% of the total flow rate that is required to obtain standard optimal hydration taking into account the volume of EN that is being delivered. If enteral feeding of at least 80% of the calculated calories is not possible after 7 days in ICU, PN, as specified below, is initiated on day 8.

The infusions will be supplemented with micronutrients and vitamins as they would have received when provided PN.

When the blood glucose levels fall spontaneously below 50 mg/dl, the standard glucose of 5% in the late PN group will be switched to 10% glucose until blood glucose level is above 80 mg/dl. At this point the infusion of glucose 10% will be stopped and switched again to glucose 5%.

Early PN", this nutritional regime is the standard therapy in the participating centres and therefore accounts as the "control-group".

Both centres will use their current pharmaceutical nutritional products for enteral as well as parenteral nutrition. The differences between the products provided by the different pharmaceutical companies are small clinically irrelevant differences in glucose, lipid and protein intakes.

Despite the different pharmaceutical companies, nutritional regimes from both centres will be aiming for the same energy and protein targets.

Fluid intake will be provided as follows; patients not requiring fluid restriction receive 100 ml/kg/day for the first 10 kg bodyweight, 50 ml/kg for the next 10 kg, and 20 ml/kg for the bodyweight over 20 kg, patients who require fluid restriction, total fluid intake is 50% on day 1 and 2, and 75% on day 3.

Nutritional target calculations

Energy intake

• < 10 kg bodyweight : 100 kcal/kg/day

• 10-20 kg bodyweight : 1000 kcal/day + (50 kcal/kg/day for weight over 10 kg)

• > 20 kg bodyweight : 1500 kcal/day + (20 kcal/kg/day for weight over 20 kg)

Protein target

• 0-10 kg bodyweight : 1.5-3 g/kg/day

• 10-20 kg bodyweight : 1-3 g/kg/day

• > 20 kg bodyweight : 1-2 g/kg/day

Day 1:

In the PICUs from both centers, patients randomised to the "early PN" group upon admission to PICU receive a glucose mixture [Leuven; Glucose15%/Vaminolact® (Fresenius), Rotterdam; Glucose 10–20% (Baxter)] to achieve glucose intake approximately double of the intake in the "late PN" group, with additional trace elements (Peditrace®) and minerals (Addamel®, Novum, Fresenius) and vitamins (Cernevit®, Soluvit®, Vitintra®; Baxter) to be administered in both centers.

Day 2-3:

For all patients on intravenous (IV) nutrition, lipids [Leuven; (20g/100ml) SMOFlipid® Fresenius, Sweden, Rotterdam; (20g/100ml) Intralipid® (Baxter), Oliclinomel® (Baxter)] are added from day 2-3 onward, and increased depending on the age and within the fluid limitation potentially required by the patient.

Additionally, protein intake [Leuven; Vaminolact® or Vamin18® (Fresenius, Sweden), Rotterdam; Primene® (Baxter), Oliclinomel® (Baxter)] will be increased to reach target goals.

On day 2-7, pharmacy-prepared PN preparations are prescribed to achieve protein and energy goals, as described above, unless adequate enteral nutritional intake is expected.

Any enterally delivered energy is taken into account twice daily to reduce the energy delivered by PN. When EN covers 80% of optimal calculated caloric needs, PN is stopped.

When the patient starts to take oral nutrition, the PN and/or EN is reduced and eventually stopped. Whenever enteral or oral intake falls below 50% of calculated caloric needs, the PN is restarted.

The volumes of PN and EN to be given according to the treatment group are calculated by the patient data management system (PDMS). These calculations are based on the nutritional intake during the previous day and the clinical evolution of the patient. The amount of protein and glucose administered during the previous day, as well as the target, will be displayed by the PDMS to further guide the prescription of macronutrients.

The additional trace elements (Peditrace®) and minerals (Addamel® Novum®, Fresenius) and vitamins (Cernevit®, Soluvit®, Vitintra®; Baxter) will be administered in both centers daily until until patients receive at least 80% of their caloric intake enterally.

Enteral nutrition and micronutrient administration

In all patients from both study arms, provided haemodynamically stable and without formal contraindication, EN is initiated on the afternoon following the ICU admission with the patients in semirecumbent position. Enteral feeding will start 6 hrs after admission in the PICU and will be done according to protocol (cfr appendix 1). Trace elements (Peditrace®) and minerals (Addamel® Novum®, Fresenius) and vitamins (Cernevit®, Soluvit®, Vitintra®; Baxter) will be administered daily IV to all patients from day 2 at 4:00 pm. IV micronutrient substitution will be stopped when patients receive at least 80% of their caloric needs via the enteral route.

In infants, breast milk, the patient's home milk formula or a protein-energy dense formula (Nutrilon®, Infatrini®; Nutricia Netherlands) is used. Older children receive standard commercially available enteral feeding (Nutrison®, Nutrini-formulas; Nutricia Netherlands) unless contra-indicated.

Enteral feeding is administered through a gastric tube in a continuous way and is gradually increased as dictated by tolerance. Switch to oral intake is made as soon as deemed safe. The increase of EN volume, the use of gastroprokinetics and duodenal feeding tubes are described in standing-orders for EN. Procedures for slow parenteral administration will be identical for all patients.

Blood glucose management

Blood glucose management will be performed as through standard practice in both participating centers. (for both protocols see the appendices)

In **Leuven**, patients in both study groups receive continuous insulin infusion to target blood glucose levels of 50-80 mg/dl when aged < 1y and 70-100 mg/dl when aged >= 1 year. Blood glucose and potassium are monitored systematically every 1 – 4 hours on the blood gas analyser (ABLRadiometer ®, Copenhagen, Denmark) using arterial blood samples.(14)

In **Rotterdam**, patients in all age groups receive continuous insulin infusion to target blood glucose levels of 72-145 mg/dl, except for patients with traumatic brain injury (108-145 mg/dl), using a step-wise nurse driven glucose control protocol. Blood glucose and potassium are monitored systematically every 1 – 3 hours on the blood gas analyser (ABL 625; Radiometer®, Copenhagen, Denmark) using arterial blood samples.(15)

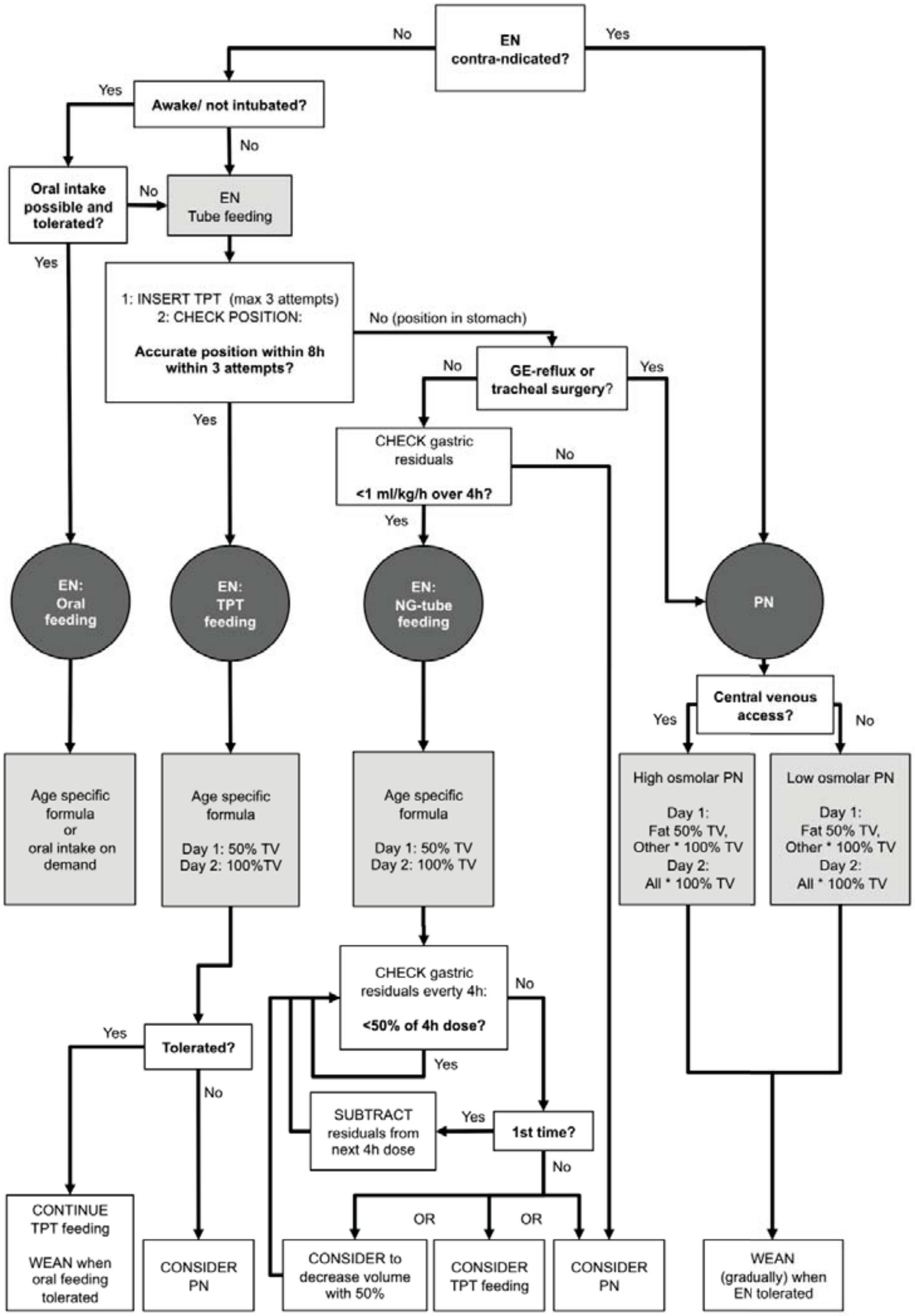

Nutritional protocol showing schematic decision tree

Handling of re-admissions to ICU

Patients who are re-admitted to ICU after a participation in PEPaNIC are not eligible for re-inclusion. Patients who are readmitted to the PICU within 48 hours of discharge and who are still within the 7 days time window of the initial randomization receive the nutrition-schedule they were assigned to during the initial ICU admission. Patients readmitted later will be fed at the discretion of the attending physician.

5.2 Use of co-intervention (if applicable)

Not applicable

5.3 Escape medication (if applicable)

Not applicable

6. INVESTIGATIONAL MEDICINAL PRODUCT

Not applicable.

7. METHODS

7.1 Study parameters/endpoints

7.1.1 Main study parameter/endpoint

The primary efficacy endpoints of for this RCT are included into **Work Package 1** and are the incidence of new infections and the time to discharge alive from ICU. Number of patients with new infections and types of infection will be assessed by numbers and percentages, and the duration of any antibiotics therapy initiated after randomization for those patients requiring antibiotics will be analyzed by non-parametrical tests. As the time of ICU discharge to the regular ward may be affected by the availability of beds on the regular wards, which could induce bias, we *a priori* decided to analyze “time to discharge from ICU” as “time to ready for discharge from ICU”. A patient is considered “ready for discharge” as soon as all clinical conditions for ICU discharge have been fulfilled (no longer in need for vital organ support). Time to discharge alive from ICU will be reported by Kaplan-Meier plots, with ICU non-survivors censored beyond the longest ICU stay of survivors and censoring time of patients still in the ICU at closing of the data file (90 days after last patient inclusion) over both treatment groups. The impact of “late PN” versus “early PN” will be analyzed, with and without correction for age, nutritional status and risk categories and type and severity of illness, by Cox proportional hazard analysis. The distribution of the actual time to discharge from ICU will be reported for ICU-survivors and ICU-non-survivors separately. In view of the time window of the randomized intervention in ICU, also the proportion of patients staying beyond 8 days in ICU will be reported.

7.1.2 Secondary study parameters/endpoints

Work Package 1

All analyses will be performed uncorrected as well as corrected for age, nutritional status and risk categories and type and severity of illness. Time-to-event analysis will be analyzed similarly as the primary endpoint. Proportion of patients requiring support of vital organ functions and distribution of duration of support will be analyzed by non-parametric or parametric testing depending on the normality of the distribution in the subgroup of patients for which support was needed. Proportions will be compared using chi-square testing. Results of repeated measurements will be analyzed using an appropriate model for longitudinal data.

a. Time to final (alive) weaning from mechanical respiratory support: patients still on mechanical respiratory support at closing of the data file (90 days after last patient inclusion) will be censored at that time point. ICU non-survivors will be censored beyond the longest duration of mechanical respiratory support of the survivors.

b. Kidney failure: Proportion of patients in need for renal replacement therapy (RRT) during ICU stay; distribution of duration of RRT (for those patients requiring RRT); proportion of patients with a post-randomization diagnosis of new kidney injury/failure

(defined by modified Risk, Injury, Failure, Loss, and End-stage Kidney (RIFLE) classification criteria as a plasma creatinine doubling or more during ICU stay) in both treatment groups. In addition, the duration of a score RIFLE≥2 will be used as a marker of time to recovery of kidney damage.

c. Need for pharmacological or mechanical haemodynamic support during ICU stay, and duration of such need.

In addition, time to final (alive) weaning from all pharmacological or mechanical haemodynamic support in ICU will be analyzed, with ICU non-survivors censored beyond the longest duration of pharmacological or mechanical haemodynamic support of the survivors and censoring time of patients still on such support at closing of the data file (90 days after last patient inclusion) over both treatment groups.

d. Number of readmissions to the PICU.

e. Liver dysfunction: Proportion of patients during the time window of the intervention and during the whole ICU stay presenting with cholestatic or cytolytic liver dysfunction will be compared.

f. Inflammation: Effect of the intervention on inflammation will be analyzed by comparing the distribution of the highest value reached during ICU stay and changes from baseline to the highest value and by comparing time profiles of daily C-Reactive Protein values.

g. Child health questionnaire scores (WEEFIM, HUI, CHQ) at hospital discharge in both treatment groups will be compared. And at follow-up at 3 and 12 months post-discharge.

Work Package 2: Health economy analysis

As PN is a major contributor to the healthcare costs of critically children and the clinical outcome is not yet known, an extensive health economic analysis will be included in the study planning. The economic analysis will be performed from a health care perspective and will be estimated for the period during hospitalization OR one year after admission?. The economic evaluation will be performed in accordance with the Dutch guidelines (27).

First, we will calculate and compare the direct medical costs of early versus late PN.

Real medical costs will be calculated by multiplying the volumes of health care use with the corresponding unit prices. *B*ased on the analysis of the patients' detailed invoices, we will allocate healthcare costs into 8 categories (per diem hospitalization costs, honoraria, pharmacy costs, clinical chemistry costs, radiology costs, blood products, graft products, miscellaneous), representing the different reimbursed services and products during ICU and hospital stay. These cost categories have been validated in the health economic analysis of the adult EPaNIC study.

*T*he drug costs will be analyzed using the first level of the World Health Organisation (9) Anatomical Therapeutic Chemical (ATC) classification. We hypothesize that antimicrobials (class J) are the determining driver for differences in pharmacy costs. This is based on the idea that late PN will reduce the incidence of new infections in the ICU.

A model will be constructed to estimate the hypothetical cost difference between early PN and late PN if all acquisition costs related to PN would have been chargeable. This modeling is important to make the results interpretable for readers in different healthcare systems. PN is in variable proportions charged to the patient directly, the hospital or the insurers. Therefore, the data from Rotterdam and Leuven will be converged to one hypothetical system of PN billing.

Costs for inpatient days in hospitals will be estimated as real, basic costs per day using detailed hospital administrative information. For the calculation of other medical costs, we will used charges as published in Dutch guidelines as a proxy of real costs (27).

All analyses will be performed on an intention-to-treat basis. The cost differences between early and late PN will be analyzed using the Mann–Whitney U test. Since cost data per patient (but not per day care) are typically highly skewed, we use nonparametric bootstrap techniques to derive a 95% confidence interval for the differences in distributions of the direct medical costs.

Furthermore, we will explore whether late PN is cost-effective compared to early PN. This will be assessed by calculating the incremental cost-effectiveness ratio (ICER), defined as the difference in costs of late versus early PN, divided by the average change in effectiveness. The primary effect measure is number of patients with a prevented new infection in ICU. Secondary outcome measure is quality of life as measured by validated questionnaires. Overall utility scores for population-based quality of life will be obtained and expressed as QALY's. QALY's will be calculated by multiplying the utility of a health state by the time spent in this health state.

*C*ost-effectiveness of late PN as compared to early PN will be analyzed as the difference in PN costs per patient with a prevented new infection in ICU. In the ideal scenario late PN will result in an improvement of the clinical outcome as well as

a reduction of the healthcare costs. If late PN conveys a clinical improvement at a higher cost, this cost effectiveness in two health care systems will be important to guide the respective governmental health care departments into the decisions of the reimbursement of PN.

For the health economic analysis we will collaborate with the finance & accounting and pharmacy departments of the two participating hospitals. Template database structures will be set-up in advance during the enrolment of the patients in the trial to allow an efficient health economic analysis in the framework of the study.

Work Package 3: Mechanistic analyses in relation to short- and long-term outcome

Impact of early versus late PN on infection and the inflammatory response

The incidence of new, ICU-acquired, infections is a primary efficacy endpoint in the presented study. Based on the results of the adult EPaNIC study (1), we anticipate a lower infection rate with late PN as compared with early PN. We will investigate whether differences in leukocyte function could explain in part such effect. Therefore, blood samples will be taken at pre-set time points during ICU stay for the isolation of monocytes and granulocytes to assess their chemotactic, phagocytosis and oxidative burst capacity (17, 18). Antimicrobial defense depends on, amongst others, efficient clearance of intracellular

pathogens in the autophagic pathway (xenophagy) (10). Indeed, inactivation of autophagy in macrophages and neutrophils has been shown to increase susceptibility to infection. It has previously been demonstrated an autophagy-deficiency phenotype in liver and skeletal muscle of fed prolonged critically ill patients (11). Importantly, a recent study in prolonged critically ill rabbits demonstrated a role for early PN in suppression of the autophagic pathway during critical illness (19). If autophagy would be similarly compromised in leukocytes by early PN, such effect may contribute to a reduced incidence of infections with late PN. We will address this question in a subset of well-matched patients. We will also measure the p62 protein levels of isolated white blood cells obtained upon admission and on day 3 and 7 in ICU, which is known to accumulate when autophagy is deficient/insufficient, as well as the LC3-II/LC3-I ratio as a marker of mature autophagosome formation (20-22). In addition, we will isolate leukocyte DNA from all patients to investigate whether genetic predisposition by single nucleotide polymorphisms (SNPs) in genes of the autophagic core machinery may play a role in susceptibility to infections and whether any such SNP would interact with the nutritional approaches of the study design. The autophagy pathway and/or proteins also appear to play a crucial role in the control of inflammatory signaling and regulation of inflammatory transcriptional responses (10). In this regard, it has been shown that increased levels of p62 activate the pro-inflammatory transcription factor NF-kB. We will study the impact of early versus late PN on pro- and anti-inflammatory cytokines (interleukin-1beta (IL-1beta), IL-2, IL-4, IL-5, IL-6, IL-8, IL-10, tumor-necrosis-factor-alpha, interferon-gamma).

Impact of early versus late PN on the neuroendocrine axes

Critical illness is hallmarked by striking alterations in the hypothalamic-pituitary-peripheral hormone axes (neuroendocrine axes), according to a biphasic pattern (23). The peripheral effector hormone levels are reduced in both phases of critical

illness, although the etiology is different with peripheral target organ resistance in the acute phase and relative hypopituitarism in the prolonged (chronic) phase.

The hypothalamic-pituitary-adrenal axis shows a biphasic, but somewhat different response as compared with the other axes, with cortisol as effector hormone being high in both phases. The responses of the neuroendocrine axes during prolonged critical illness overall have been linked to the development of a characteristic hypercatabolic state, resulting in feeding-resistant muscle wasting, and the severity of the disturbances has been associated with the high risk of morbidity and mortality of the patients. Not only excessive activation of the hypothalamic-pituitary-adrenal axis, but also adrenal insufficiency contributes to morbidity in critically ill children (24).

It was previously hypothesized that strict blood glucose control with intensive insulin therapy would mildly reactivate the somatotropic and thyrotropic axes and anabolism in critically ill children in view of a larger functional capacity of the hypothalamus and pituitary, and the higher amount of nutrition they receive as compared with adult critically ill patients. Unexpectedly and despite improved ICU outcome, however, this therapy further suppressed the somatotropic axis and increased the urea/creatinine ratio as a marker of catabolism (25). Also the low-T3 syndrome could not be reversed by this therapy (unpublished data).

Unpublished data obtained in a rabbit model of prolonged critical illness suggest that relative fasting during critical illness reduces (peripheral) activation of the somatotropic and thyrotropic axes with lower levels of insulin-like growth factor-I (IGF-I) and triiodothyronine (T3) levels, but also attenuates the rise in the stress hormone cortisol as compared with moderate dose intravenous feeding during critical illness. These findings urge a thorough analysis of the impact of early versus late PN on these neuroendocrine axes in the critically ill children included in our study. We hypothesize that late PN results in suppression of the neuroendocrine axes, in concert with the intensive insulin therapy. Unlike conventional belief, we also hypothesize that such suppressed neuroendocrine axes during prolonged critical illness could be associated with a beneficial acute and long-term clinical outcome.

We will evaluate the activation of the somatotropic axis in the acute and chronic phase of critical illness by measuring the levels of growth hormone (GH), acid-labile subunit (ALS), IGF-I and its binding proteins (IGFBP-1 and IGFBP-3), the activation of the thyrotropic axis by analysis of thyroxin (T4), T3, reverse T3 (rT3) and thyroid stimulating hormone (TSH), and the adrenal axis by adrenocorticotropic hormone (ACTH) and cortisol. The metabolism of cortisol will be studied further by the relative levels of its metabolites excreted in urine (26). The impact of early versus late PN on the neuroendocrine axes will be studied in relation to markers of catabolism, muscle volume and strength, as well as clinical outcome. The clinical outcome will be assessed during follow-up consultations that are held in the departments that have referred their patients to the ICU in Leuven and Rotterdam.

The epigenetics of early versus late PN

It has been known since a long time that dietary exposures can have health consequences years or decades later. E.g. the later health of babies who were in the womb during the Dutch famine in the winter of 1944 was greatly affected by the caloric restriction of their mothers. The children of pregnant women exposed to famine were shown to be more susceptible to chronic adult diseases such as diabetes, obesity and cardiovascular disease. Apparently, the short period of caloric

restriction can be “remembered” by the body further on in life. There is a growing body of evidence that epigenetic mechanisms mediate this memory. Epigenetic mechanisms alter the gene expression without changing the primary DNA sequence. Rather the epigenetic mechanisms work through DNA methylation, histone

modifications and non-coding microRNAs. During the clinical trial, biological material (spun-down cells from whole blood samples) will be collected from the study patients to compare these epigenetic changes between the early and late PN treatment groups. The mechanistic findings will be correlated with the acute and the long-term outcome of the critically ill study patients.

7.1.3 Other study parameters

Safety endpoints

Safety endpoints comprise vital status, hypoglycemia, SAEs and complications related to the mode of nutrition. Survival up to 90 days after randomization in both treatment groups will be compared by Kaplan Meier survival plots. The impact of “late PN” versus “early PN” will be analyzed, with and without correction for risk factors, by Cox proportional hazard analysis. In addition, we will record in-ICU and in-hospital mortality, and will analyze differences with Chi-square testing. As the randomized study intervention only takes place during a time window up to the 8th day in ICU, we also plan to analyze early lethality within this time window in the intention-to-treat population as part of the safety analysis.

As patients not receiving early PN may be considered at increased risk for hypoglycemia, we will report for both groups the number of patients experiencing hypoglycemia <40 mg/dl during the time window of the randomized intervention. Hypoglycemia resistant to parenteral glucose administration is considered as a SAE and the incidence during the time window of the randomized intervention will be reported for both groups. In addition, overall blood glucose control during the time window of the intervention will be compared using daily morning blood glucose as well as daily maximal and minimal blood glucose.

Safety issues also comprise the occurrence of feeding-mode-related complications during the time window of the intervention. Therefore, occurrence of these complications (digestive intolerance, complicated insertion of feeding tubes, pneumothorax, haemothorax and subclavian or carotid artery puncture, occlusion or displacement of central venous catheters or gastric feeding tubes) will be reported for both treatment groups.

7.2 Randomisation, blinding and treatment allocation

Randomization

The study will use a prospective, randomized, controlled, parallel-group design. On admission patients will be randomly assigned to receive EN combined with early PN or only EN. At ICU admission, consecutive patients will be randomly assigned to one of these two treatment groups using a centralized computer randomisation. Randomisation will be done in a 1:1 allocation ratio in permuted blocks of 10 (The block size remains unknown to bed-side physicians and nurses, responsible for patient recruitment and therapy assignment) and stratified according to primary diagnostic category on admission:

I Medical-ICU admissions (infectious or non-infectious): (a) respiratory (b) cardiac (c) renal (d) haematological/oncological (e) gastro-intestinal/hepatic (f) neurological (g) other.

II Surgical-ICU admissions (elective or urgent) according to referral discipline (a) cardiac surgery (b) solid organ transplants (c) pulmonary/oesophageal surgery (d) abdominal surgery (e) neurosurgery (f) trauma/orthopaedic surgery (g) burns (more than 20% BSA is burned and/or patient requires ventilation)

Blinding of treatment allocation

Treating physicians and patients cannot be blinded. All outcome assessors, which are investigators not directly involved in the patients care (such as statisticians, laboratory personnel, infectious disease specialists, pathologists and physiotherapists involved in the strength measurement) as well as physicians and nurses in the conventional wards are blinded to treatment allocation.

7.3 Study procedures

Data collection following recruitment

Baseline characteristics

At baseline, data on demographic and clinical characteristics of the patients are obtained. Disease specific risk scores (such as the Risk-Adjustment in Congenital Heart Surgery or RACHS score) are calculated, co-morbidities and known use of important medications prior to admission are noted: these comprise, among others, the presence of congenital disease or syndrome, gestational age at birth, gender, ethnicity, paediatric risk scores, presence / history of cancer, diabetes mellitus, kidney failure, liver failure, chronic heart failure and sepsis upon admission. In addition, we record the need for and the number of days of mechanical ventilatory support, of mechanical and pharmacological haemodynamic support, of renal replacement therapies, days on antibiotics and days requiring a central line.

Outcome characteristics

All medications received by the patients during ICU stay are registered. Every day the amount of kilocalories, lipids, proteins, carbohydrates delivered by either PN or EN are calculated from the PDMS in an automated manner and entered into the case record form (CRF). Interruptions of EN delivery and predefined digestive intolerance are registered daily. Mechanical complications such as displacement or obstruction of the enteral feeding tube or the central venous catheter, and clinical complications such as pneumothorax, hemothorax and subclavian or carotid artery puncture are recorded daily. All medications received by the patients during ICU stay are registered. Every day the amount of kilocalories, lipids, proteins, carbohydrates delivered by either PN or EN are calculated from the PDMS in an automated manner and entered into the case record form (CRF). Of the gastric residual volume discarded, half of the volume will be considered to be EN and half gastric secretions. The duration (in min) and cause of interruption of delivery of EN will be recorded. Digestive

intolerance will be registered as vomiting, tracheal aspiration of enteral feeding (defined 8), diarrhea, and gastric residue above 5ml/kg (see appendix for definitions). Tube displacement or obstruction will be labeled as mechanical complications. Occlusion and dislodging of central venous catheters will be recorded as mechanical complications. Pneumothorax, hemothorax and arterial puncture will be recorded as clinical complications. Number of ICU days with a central line in situ will be noted. The characteristics will be monitored until discharge from ICU, day 90 of hospital stay or death.

Blood samples

The maximum blood volume will be maximum 5 ml/kg for the entire study period. For a detailed description of the blood samples and tests see figure below. Blood samples are taken upon ICU admission and daily until discharge from ICU, or death. The blood samples will be taken from lines placed for clinical purposes or in combination with pricks requested for clinical purposes. A subset of the samples are immediately stored on ice for future endocrine measurements. Processed serum and plasma as well as the spun-down blood cells will be stored and frozen for mechanistic analyses.

All whole blood glucose levels are measured on arterial blood using a blood gas analyser on each ICU and are registered for later calculation of glucose metrics. Analyses on blood and urine for the primary clinical analyses include routine chemistry, haematology, and markers of inflammation. Further epigenetic, metabolic, endocrine and inflammatory measurements on stored samples in the context of mechanistic analyses will be stored until final analysis. Urine samples will be stored and frozen until final analysis.

Primary endpoint characteristics

All new infections of the lungs, the blood stream, the urinary tract and wounds are recorded by an infectious disease specialist. Bacteraemia is further classified by responsible pathogen and as catheter-related blood stream infection versus other bacteraemia.

Additional data collection

For mechanistic and exploratory studies, muscle strength testing (> 6 years of age) at discharge and an ultrasound evaluation during admission of the skeletal muscle, liver, and adipose tissue compartments will be performed.

Urine (nitrogen / bone metabolism / cortisol metabolites / biomarkers for renal failure; NGAL) and stool samples (biomarkers gut function; citrulline / calprotectin) will be collected on daily basis.

We will also perform standard anthropometric tests, upper-arm and lower-leg circumference, in addition to standard weight and height for age. These analyses will be repeated at the follow-up at 3 and 12 months post-discharge, on condition of obtaining adequate additional funding.

Questionnaires and long term follow-up

Furthermore, as far as practically feasible, every patient is approached just prior to hospital discharge, to complete a validated, semi-structured child health questionnaire (Functional Independence Measure (WEEFIM), Health Utilities Index (HUI) and Child Health Questionnaire (CHQ)).

We also will thoroughly assess long-term clinical outcome. This not only encompasses survival rate 3 years after inclusion in the study. It will also involve a detailed functional and neurocognitive examination, by means of questionnaires such as mentioned above. These analyses will be repeated at the follow-up at 3 and 12 months post-discharge, on condition of obtaining adequate additional funding. This will be organised in the framework of current follow-up consultations of these critically ill children.

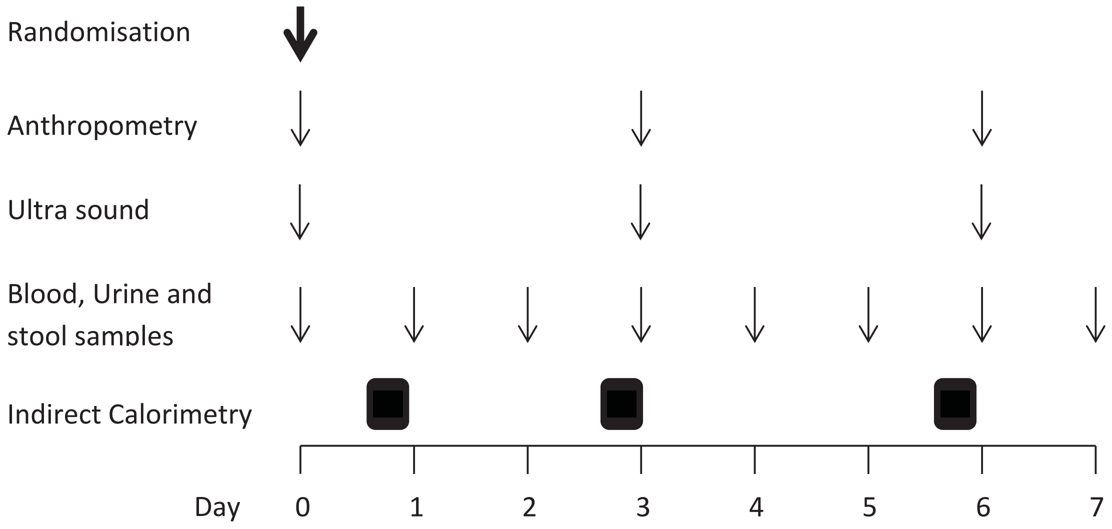

Figure Samples.

Blood samples are taken upon ICU admission and daily at 06:00h until discharge from ICU, death or end of study. Upon admission three blood samples will be taken; namely two yellow "STOL"-tubes each containing a maximum of 3.5 milliliter blood and 1 purple "EDTA"-tube of 1.8 milliliter blood.

During the following days until the patient has left the PICU we will daily take a yellow "STOL"-tube (containing max 3,5 milliliter blood) and a purple "EDTA"-tube (containing 1,8 milliliter blood) for further analyses.

A subset of the samples will be immediately stored on ice for future endocrine measurements. Processed serum and plasma as well as the spun-down blood cells will be frozen and stored.

Analyses on the morning sample will include routine clinical chemistry, hematology (Hgb, WBC, TC), and markers of inflammation (CRP), liver function ALT, AST, ALP, GGT, bilirubin total/direct. A number of metabolic, hormonal and inflammatory study analyses will be performed on selected days.

The latter comprise, among others, coagulation & fibrinolysis tests, cytokines and markers of oxidative stress.

Also on selected days stool (single sample) urine (5 tubes of 5 mL out of 24 hours' urine collection) will be stored for further evaluation.

All whole blood glucose levels are measured on arterial blood using a blood gas analyser on each ICU and are registered for later calculation of glucose metrics.

7.4 Withdrawal of individual subjects

Subjects can leave the study at any time for any reason if they wish to do so without any consequences. The investigator can decide to withdraw a subject from the study for urgent medical reasons.

7.4.1 Specific criteria for withdrawal (if applicable)

Not applicable

7.5 Replacement of individual subjects after withdrawal

Subjects that withdraw after randomization has been conducted will not be replaced, subjects that have not been allocated and withdraw after signing the consent form but before the start of randomization will be replaced.

7.6 Follow-up of subjects withdrawn from treatment

A register is kept of all patients evaluated for inclusion and of patients who withdraw from the study. The latter are clinically followed up without their data being analyzed in the study, according to intention-to-treat analysis.

7.7 Premature termination of the study

As the previous study in adults has shown that lethality was not different among the study groups and that morbidity was prevented by withholding feeding, and since the participating centers are currently providing early PN, repeated interim analyses for efficacy are not required. However, one formal "safety" interim analysis will be planned after patient number 720 has left the ICU, during which the independent DSMB will have access to un-blinded results on ICU mortality, hospital mortality and serious adverse events. The DSMB will then judge on whether or not to prematurely stop the clinical trial for safety reasons, or to perform more safety interim analyses. The DSMB will advise on eventual continuation of the study to completion, under monitoring of the serious adverse event. Because the primary efficacy

endpoint will not be analyzed, no correction of the significance level at the final analysis will be necessary. In (the unlikely) case the study would be terminated prematurely, the mechanistic studies will be even more important, as the clinical community will ask for due explanations for harm caused during the study. Therefore, in any case they will be started shortly after all clinical data have been collected.

8. SAFETY REPORTING

The risk in participating to the study and being randomized to the "late PN' group are negligible, and specifically compass an increased risk of developing hypoglycemia and/or underfeeding. However, safety measures will be taken to further decrease these risks. Our study may provide support for current practice, may give “neutral” results for which the cost-effectiveness study will provide necessary information for guiding therapy, or may challenge the presumed innocence of PN. Thus, there is no risk associated with the intrinsic value of these results.

The burden is expected to be minimal as it will only entail additional blood draws, which will be taken from clinical lines or in addition to pricks for clinical purpose. The long-term follow up will be held as part of already organised follow-up outdoor clinics and which are developed to help children and their parents to recover physically, emotionally and socially after ICU admissions. For mechanistic and exploratory studies, muscle strength testing and an ultrasound evaluation of the skeletal muscle and adipose tissue compartments will be performed. Furthermore, as far as practically feasible, every patient is approached just prior to hospital discharge, to complete a validated, semi-structured child health questionnaire (Functional Independence Measure (WEEFIM), Health Utilities Index (HUI) and Child Health Questionnaire (CHQ)).

In order to monitor the quality of the enteral and parenteral nutrition management during the study we will register all known complications possibly related to them. These complications should not be considered as adverse events since the study intervention is to withhold parenteral feeding during one week. These known complications of parenteral feeding will not be reported to the sponsor until the end of the trial.

***Complications possibly related to enteral feeding**

Digestive intolerance: either vomiting, tracheal aspiration of enteral feeding (defined in 7), diarrhea, or gastric residue above 5ml/kg, abdominal distention (see appendix for definitions).
Complicated insertion of feeding tubes: nasal bleeding
Mechanical complications Feeding tube displacement or obstruction

***Complications possibly related to parenteral feeding**

Mechanical complications Occlusion and dislodging of central venous catheters
Clinical complications: pneumothorax, hemothorax and arterial puncture, central line replacement due to suspicion of catheter-related blood stream infections

Safety endpoints

Safety endpoints comprise vital status, hypoglycaemia, SAEs and complications related to the mode of nutrition. Survival up to 90 days after randomisation in both treatment groups will be compared by Kaplan Meier survival plots. The impact of “late PN” versus “early PN” will be analysed, with and without correction for risk factors, by Cox proportional hazard analysis. In addition, we will record in-ICU and in-hospital mortality, and will analyse differences with Chi-square testing. As the

randomised study intervention only takes place during a time window up to the 8th day in ICU, we also plan to analyse early lethality within this time window in the intention-to-treat population as part of the safety analysis.

As patients not receiving early PN may be considered at increased risk for hypoglycaemia, we will report for both groups the number of patients experiencing hypoglycaemia <40 mg/dl during the time window of the randomised intervention. Hypoglycaemia resistant to parenteral glucose administration is considered as a SAE and the incidence during the time window of the randomised intervention will be reported for both groups. In addition, overall blood glucose control during the time window of the intervention will be compared using daily morning blood glucose as well as daily maximal and minimal blood glucose.

Safety issues also comprise the occurrence of feeding-mode-related complications during the time window of the intervention. Therefore, occurrence of these complications (digestive intolerance, complicated insertion of feeding tubes, pneumothorax, haemothorax and subclavian or carotid artery puncture, occlusion or displacement of central venous catheters or gastric feeding tubes) will be reported for both treatment groups

Interim analysis

As the previous study in adults has shown that lethality was not different among the study groups and that morbidity was prevented by withholding feeding, and since the participating centres are currently providing early PN, repeated interim analyses for efficacy are not required. However, one formal "safety" interim analysis will be planned after patient number 720 has left the ICU, during which the independent DSMB will have access to un-blinded results on ICU mortality, hospital mortality and serious adverse events. The DSMB will then judge on whether or not to prematurely stop the clinical trial for safety reasons, or to perform more safety interim analyses. The DSMB will advise on eventual continuation of the study to completion, under monitoring of the serious adverse event. Because the primary efficacy endpoint will not be analysed, no correction of the significance level at the final analysis will be necessary.

8.1 Section 10 WMO event

In accordance to section 10, subsection 1, of the WMO, the investigator will inform the subjects and the reviewing accredited METC if anything occurs, on the basis of which it appears that the disadvantages of participation may be significantly greater than was foreseen in the research proposal. The study will be suspended pending further review by the accredited METC, except insofar as suspension would jeopardise the subjects' health. The investigator will take care that all subjects are kept informed.

8.2 Adverse and serious adverse events

Adverse events are defined as any undesirable experience occurring to a subject during the study, whether or not considered related to the experimental treatment. All adverse events reported spontaneously by the subject or observed by the investigator or his staff will be recorded.

A serious adverse event is any untoward medical occurrence or effect that at any dose:

- results in death;
- is life threatening (at the time of the event);
- requires hospitalisation or prolongation of existing inpatients' hospitalisation;
- results in persistent or significant disability or incapacity;
- is a new event of the trial likely to affect the safety of the subjects, such as an unexpected outcome of an adverse reaction, lack of efficacy of an IMP used for the treatment of a life threatening disease, major safety finding from a newly completed animal study, etc.

All SAEs will be reported through the web portal *ToetsingOnline* to the accredited METC that approved the protocol, within 15 days after the sponsor has first knowledge of the serious adverse reactions. SAEs that result in death or are life threatening should be reported expedited. The expedited reporting will occur not later than 7 days after the responsible investigator has first knowledge of the adverse reaction. This is for a preliminary report with another 8 days for completion of the report.

Safety endpoints specifically for PEPaNIC

The clinical research team guarantees a daily follow-up of patient screening and inclusion, availability of requested clinical data in the clinical patient files and protocol compliance. Each non-compliance to the protocol and other questions or problems are reported to the study monitor and discussed with the principal investigators. Serious Adverse Events (SAE) are also reported to the study coordinating investigator/project leader (K.U.Leuven). The study monitor regularly provides the sponsor with reports on inclusions and SAE. Regular meetings are organized with principal investigators and clinical research team to discuss the daily progression of the research project.

As patients not receiving early PN may be considered at increased risk for hypoglycemia, we will report for both groups the number of patients experiencing hypoglycemia <40 mg/dl during the time window of the randomized intervention. Hypoglycemia resistant to parenteral glucose administration, or with clinical symptoms, is considered as a SAE and the incidence during the time window of the randomized intervention will be reported for both groups. In addition, overall blood glucose control during the time window of the intervention will be compared using daily morning blood glucose as well as daily maximal and minimal blood glucose.

Further safety endpoints comprise vital status, hypoglycemia, SAEs and complications related to the mode of nutrition. Survival up to 90 days after randomization in both treatment groups will be compared by Kaplan Meier survival plots. The impact of "late PN" versus "early PN" will be analyzed, with and without correction for risk factors, by Cox proportional hazard analysis. In addition, we will record in-ICU and in-hospital mortality, and will analyze differences with Chi-square testing. As the randomized study intervention only takes place during a time window up to the 8th day in ICU, we also plan to analyze early lethality within this time window in the intention-to-treat population as part of the safety analysis.

Safety issues also comprise the occurrence of feeding-mode-related complications during the time window of the intervention. Therefore, occurrence of these complications (digestive intolerance, complicated insertion of feeding tubes, pneumothorax, haemothorax and subclavian or carotid artery puncture, occlusion or displacement of central venous catheters or gastric feeding tubes) will be reported for both treatment groups.

Above mentioned SAE's and safety endpoints will be handled and reported within the standard timeframe as described above.

8.2.1 Suspected unexpected serious adverse reactions (SUSAR)

Not applicable

8.2.2 Annual safety report

Not applicable

8.3 Follow-up of adverse events

All adverse events will be followed until they have abated, or until a stable situation has been reached. Depending on the event, follow up may require additional tests or medical procedures as indicated, and/or referral to the general physician or a medical specialist.

8.4 Data Safety Monitoring Board (DSMB)

The coordinating investigator/project leader (K.U.Leuven) provides direct access to the CRF, the source data and the study master file for monitoring, Independent Ethics committee review and regulatory inspection. The coordinating investigator/project leader (K.U.Leuven) established an independent data safety monitoring board (DSMB) (Prof. dr. J Vranckx, Prof. dr. em. R Bouillon, Prof. dr. em. P Lauwers, Prof.dr. M Bruynooghe (statisticus)), which holds no conflicts of interest with the sponsor or coordinating investigator/project leader (K.U.Leuven). The coordinating investigator/project leader (K.U.Leuven) appoints one monitor. The monitor verifies that the trial is performed in accordance to the protocol as described in the European Medicine Agency's "Note for guidance on good clinical practice CPMP/ICH/135/95" as well as the Declaration of Helsinki. Monitoring will be performed and will be reported following the sponsor's standing operating procedures. No fault insurance is covered by Fortis Corporate Insurance NV (Leuven) and Akkermans Van Elten Assurantiën BV (Rotterdam).

One formal "safety" interim analysis will be planned after patient number 720 has left the ICU, during which the independent DSMB will have access to un-blinded results on ICU mortality, hospital mortality and serious adverse events. The DSMB will

then judge on whether or not to prematurely stop the clinical trial for safety reasons, or to perform more safety interim analyses. The DSMB will advise on eventual continuation of the study to completion, under monitoring of the serious adverse event. Because the primary efficacy

endpoint will not be analyzed, no correction of the significance level at the final analysis will be necessary.

The advice(s) of the DSMB will be notified upon receipt by the sponsor to the METC that approved the protocol. With this notification a statement will be included indicating whether the advice will be followed.

9. STATISTICAL ANALYSIS

9.1 Descriptive statistics

The current statistical analysis plan comprises the primary and secondary clinical endpoints of this RCT (work package 1). These include the acute clinical effects of the intervention during ICU stay and hospitalization, including survival up to 90 days after randomization.

The sample size (N=1440, 720 per arm) is calculated in order to detect, with at least 80% power (one- tailed; the two-tailed power is 70%) and 95% certainty, a reduction in PICU infections from 20% to 15% and, with at least 90% power (2-tailed) and 95% certainty, a reduction in mean duration of stay in PICU of 1 day. With this sample size, and for safety reasons, also any substantial impact on mortality (increase or decrease with an absolute +/- 2%, although unexpected taken the adult data), can be excluded with a power of around 62-75% depending on the true mortality in the total population. We plan to calculate the true power of the study for detection of any eventual smaller differences in these outcomes.

General rules of the statistical analysis

All analyses will be done on intention to treat basis. The analyses will be performed on the whole set of patients and by subgroups of patients based on the primary diagnostic categories used as prognostic factors for stratification and on septic/non-septic groups of patients.

A consort diagram will be reported. The data file will be finalised 90 days after inclusion of the last patient. To assess compliance with the study protocol, the amounts of PN and EN actually given in the two study groups during the intervention window of 7 days will be reported as absolute numbers and percentages of target calories. Discrete variables will be summarised by frequencies and percentages and analysed by (exact) Chi- square test or logistic regression analysis. Continuous variables will be summarised by use of either mean or standard deviations (SD) or median and interquartile range as appropriate and compared using Student's t-test or Mann-Whitney-U test, as appropriate. Time to event analysis will be performed by Cox proportional hazard analysis. All outcomes will be analysed in an uncorrected manner as well as (jointly) corrected for risk factors (type and severity of illness, age, on admission nutritional status and risk scores). *A priori* defined subgroup analyses will be performed for patients admitted to ICU after cardiac surgery as compared with all other patients; for patients with and without sepsis upon admission; for patients with contra-indications for EN on admission or not. For all endpoints, differences will be considered statistically significant whenever the p-value is lower than 0.05 without correction for multiple testing.

Safety endpoints

Safety endpoints comprise vital status, hypoglycaemia, serious adverse events and complications related to the mode of nutrition. Survival up to 90 days after randomization in both treatment groups will be compared by Kaplan Meier survival plots. The impact of "late PN" versus "early PN" will be analyzed, with and without correction for risk factors, by Cox

proportional hazard analysis. In addition, we will record vital status at ICU and hospital discharge, and will analyze differences with Chi-square testing. As the randomized study intervention only takes place during a time window up to the 8th day in ICU, we also plan to analyze early lethality within this time window in the intention to treat population as part of the safety analysis.

9.2 Univariate analysis

Discrete variables will be summarized by frequencies and percentages and analyzed by (exact) Chi-square test or logistic regression analysis. Continuous variables will be summarized by use of either mean or standard deviations (SD) or median and interquartile range as appropriate and compared using Student's t-test or (exact) Mann-Whitney-U test, as appropriate. Time to event analysis will be performed by Cox proportional hazard analysis. Time to discharge alive from ICU will be reported by Kaplan-Meier plots, with ICU non-survivors censored beyond the longest ICU stay of survivors and censoring time of patients still in the ICU at closing of the data file (90 days after last patient inclusion) over both treatment groups. The impact of "late PN" versus "early PN" will be analyzed, with and without correction for age, nutritional status and risk categories and type and severity of illness, by Cox proportional hazard analysis. The distribution of the actual time to discharge from ICU will be reported for ICU-survivors and ICU-non-survivors separately. In view of the time window of the randomized intervention in ICU, also the proportion of patients staying beyond 8 days in ICU will be reported.

9.3 Multivariate analysis

All outcomes will be analyzed in an uncorrected manner as well as (jointly) corrected for risk factors (type and severity of illness, age, on admission nutritional status and risk scores). Another *a priori* defined subgroup analysis will be performed for patients admitted to ICU after cardiac surgery as compared with all other patients. Also an *a priori* subgroup analysis is planned for patients with and without sepsis upon admission.

All analyses will be performed uncorrected as well as corrected for age, nutritional status and risk categories and type and severity of illness. Time-to-event analysis will be analyzed similarly as the primary endpoint. Proportion of patients requiring support of vital organ functions and distribution of duration of support will be analyzed by non-parametric or parametric testing depending on the normality of the distribution in the subgroup of patients for which support was needed. Proportions will be compared using chi-square testing. Results of repeated measurements will be analyzed using an appropriate model for longitudinal data.

a. Time to final (alive) weaning from mechanical respiratory support: patients still on mechanical respiratory support at closing of the data file (90 days after last patient inclusion) will be censored at that time point. ICU non-survivors will be censored beyond the longest duration of mechanical respiratory support of the survivors.

b. Kidney failure: Proportion of patients in need for renal replacement therapy (RRT) during ICU stay; distribution of duration of RRT (for those patients requiring RRT); proportion of patients with a post-randomization diagnosis of new kidney injury/failure (defined by modified Risk, Injury, Failure, Loss, and End-stage Kidney (RIFLE) classification criteria as a plasma creatinine doubling or more during ICU stay) in both treatment groups. In addition, the duration of a score RIFLE≥2 will be used as a marker of time to recovery of kidney damage.

c. Need for pharmacological or mechanical hemodynamic support during ICU stay, and duration of such need.

In addition, time to final (alive) weaning from all pharmacological or mechanical hemodynamic support in ICU will be analyzed, with ICU non-survivors censored beyond the longest duration of pharmacological or mechanical hemodynamic support of the survivors and censoring time of patients still on such support at closing of the data file (90 days after last patient inclusion) over both treatment groups.

d. Number of readmissions to the PICU.

e. Liver dysfunction: Proportion of patients during the time window of the intervention and during the whole ICU stay presenting with cholestatic or cytolytic liver dysfunction will be compared.

f. Inflammation: Effect of the intervention on inflammation will be analyzed by comparing the distribution of the highest value reached during ICU stay and changes from baseline to the highest value and by comparing time profiles of daily C-Reactive Protein values.

g. Child health questionnaire scores (WEEFIM, HUI, CHQ) at hospital discharge in both treatment groups will be compared.

9.4 Interim analysis

As the previous study in adults has shown that lethality was not different among the study groups and that morbidity was prevented by withholding feeding, and since the participating centers are currently providing early PN, repeated interim analyses for efficacy are not required. However, one formal "safety" interim analysis will be planned after patient number 720 has left the ICU, during which the independent DSMB will have access to un-blinded results on ICU mortality, hospital mortality and serious adverse events. The DSMB will then judge on whether or not to prematurely stop the clinical trial for safety reasons, or to perform more safety interim analyses. The DSMB will advise on eventual continuation of the study to completion, under monitoring of the serious adverse event. Because the primary efficacy

endpoint will not be analyzed, no correction of the significance level at the final analysis will be necessary.

The safety analyses will be done on the whole set of patients and by subgroups of patients based on the primary diagnostic categories used as prognostic factors for stratification and on septic/non-septic groups of patients.

10. ETHICAL CONSIDERATIONS

10.1 Regulation statement

The study will be conducted in accordance to the protocol as described in the European Medicine Agency's "Note for guidance on good clinical practice CPMP/ICH/135/95" as well as the Declaration of Helsinki (*59th WMA General Assembly, Seoul, Korea, October 2008*) and in accordance with the Medical Research Involving Human Subjects Act (WMO).

10.2 Recruitment and consent

The study protocol and consent forms will be sent for approval by the Institutional Review Board of the Katholieke Universiteit Leuven and the Erasmus MC (Lokale uitvoerbaarheid) and by the competent Belgian and Dutch authorities (CCMO). Written informed consent is obtained from the parents or the legal guardian by the investigator's team or one of the supervising doctors, who will inform the parents of the patients (and the patients themselves if the age is > 12 years) and ask for their consent. The parents / legal guardians and (if applicable) the patients will receive a patient information letter and an informed consent form.

Written informed consent will be obtained from the patient or the closest family member or legal guardian. For planned PICU admissions after elective procedures, informed consent will be asked beforehand, if possible.

For emergency PICU admissions, treatment allocation will be done after assessment of the patient for eligibility by the attending physician within the time frame of two hours. If eligible, the patient will be randomized into the study and the allocated glucose infusion (with micronutrients) will be started. Informed consent will be asked within the time frame of 24 hours (deferred informed consent) as a nutritional regimen has to be initiated already on admission. When consent is given, the allocated nutritional regimen will proceed (Early (with additional lipids and protein targeting nutritional goals < day3) vs Late (no PN)). When NO consent is given, the nutritional regimen and glucose infusion will be placed under responsibility of the supervising clinical team of doctors.

The parents or legal guardians can withdraw the patient from the study at any time, without penalty or impact on treatment.

10.3 Objection by minors or incapacitated subjects (if applicable)

The children can withdraw from the study at any time, without penalty or impact on treatment. (art 4 lid 2 WMO) Neither will the child be forced to undergo the additional tests, such as muscle strength testing, the ultrasound evaluation of the skeletal muscle and adipose tissue compartments, or the questionnaires and follow-up evaluations. In this matter, also non-verbal resistance will be taken into account by the investigator's team. The specific test will then not be performed, without taking the subject out of the study as a whole.

10.4 Benefits and risks assessment, group relatedness

During the informed consent process, it will be made extremely clear that participation in this study will provide no direct benefits to the patient and that refusal to participate will have zero impact on the care received by any of the nursing medical staff. The risks will be kept to a minimum. This study requires this specific study group of critically ill children of different age groups. Although the study has been performed in adults, these results should not be translated directly to children as both the metabolic as well as nutritional kinetics in children of different age groups vary significantly form adults.

10.5 Compensation for injury

The sponsor/investigator has a liability insurance which is in accordance with article 7, subsection 6 of the WMO.

No fault insurance is covered by Fortis Corporate Insurance NV (Leuven) and Akkermans Van Elten Assurantiën BV (Rotterdam).

The sponsor has an insurance which is in accordance with the legal requirements in the Netherlands (Article 7 WMO and the Measure regarding Compulsory Insurance for Clinical Research in Humans of 23th June 2003). This insurance provides cover for damage to research subjects through injury or death caused by the study.

1. € 450.000,-- (i.e. four hundred and fifty thousand Euro) for death or injury for each subject who participates in the Research;
2. € 3.500.000,-- (i.e. three million five hundred thousand Euro) for death or injury for all subjects who participate in the Research;
3. € 5.000.000,-- (i.e. five million Euro) for the total damage incurred by the organisation for all damage disclosed by scientific research for the Sponsor as 'verrichter' in the meaning of said Act in each year of insurance coverage.

The insurance applies to the damage that becomes apparent during the study or within 4 years after the end of the study.

10.6 Incentives

Not applicable

11. ADMINISTRATIVE ASPECTS AND PUBLICATION

11.1 Handling and storage of data and documents

Data are collected electronically in a pseudonymized CRF, unambiguously linked to the source file. The subject identification codes are safeguarded by the principle investigators. Data are manually transferred (and checked for accuracy) into the CRF by the clinical research assistance team on a daily basis from the ICU PDMS and the Leuven University Hospitals Clinical Working Station (KWS). Extensive range and consistency checks are performed by the study monitor. Vital status at 90 days will be recorded for all patients, by the National Death Registries when this information is not available in the hospital information system or the regional network of pediatricians.

The handling of personal data will comply with the Dutch Personal Data Protection Act (in Dutch: De Wet Bescherming Persoonsgegevens, WBP).

11.2 Amendments

Amendments are changes made to the research after a favourable opinion by the accredited METC has been given. All amendments will be notified to the METC that gave a favourable opinion.

All substantial amendments will be notified to the METC and to the competent authority.

Non-substantial amendments will not be notified to the accredited METC and the competent authority, but will be recorded and filed by the sponsor.

11.3 Annual progress report

The sponsor/investigator will submit a summary of the progress of the trial to the accredited METC once a year. Information will be provided on the date of inclusion of the first subject, numbers of subjects included and numbers of subjects that have completed the trial, serious adverse events/ serious adverse reactions, other problems, and amendments.

11.4 End of study report

The investigator will notify the accredited METC of the end of the study within a period of 8 weeks. The end of the study is defined the time-point 90 days after the inclusion of the last patient.

In case the study is ended prematurely, the investigator will notify the accredited METC, including the reasons for the premature termination.

Within one year after the end of the study, the investigator/sponsor will submit a final study report with the results of the study, including any publications/abstracts of the study, to the accredited METC.

11.5 Public disclosure and publication policy

This is an investigator initiated study. This study will be registered as a clinical trial in a public trial registry.

The investigators aim to publish all results obtained from the study unreservedly.

12. REFERENCES

1. Casaer MP, Mesotten D, Hermans G, Wouters PJ, Schetz M, Meyfroidt G, et al. Early versus late parenteral nutrition in critically ill adults. N Engl J Med 2011;365(6):506-17.

2. Singer P, Berger MM, Van den Berghe G, Biolo G, Calder P, Forbes A, et al. ESPEN Guidelines on Parenteral Nutrition: intensive care. Clin Nutr 2009;28(4):387-400.

3. Skillman HE, Wischmeyer PE. Nutrition therapy in critically ill infants and children. JPEN J Parenter Enteral Nutr 2008;32(5):520-34.

4. Zamberlan P, Delgado AF, Leone C, Feferbaum R, Okay TS. Nutrition therapy in a pediatric intensive care unit: indications, monitoring, and complications. JPEN J Parenter Enteral Nutr 2011;35(4):523-9.

5. Hulst JM, Zwart H, Hop WC, Joosten KF. Dutch national survey to test the STRONGkids nutritional risk screening tool in hospitalized children. Clin Nutr 2010;29(1):106-11.

6. Verbruggen SC, Coss-Bu J, Wu M, Schierbeek H, Joosten KF, Dhar A, et al. Current recommended parenteral protein intakes do not support protein synthesis in critically ill septic, insulin-resistant adolescents with tight glucose control. Crit Care Med 2011;39(11):2518-25.

7. Verbruggen SC, Schierbeek H, Coss-Bu J, Joosten KF, Castillo L, van Goudoever JB. Albumin synthesis rates in post-surgical infants and septic adolescents; influence of amino acids, energy, and insulin. Clin Nutr 2011;30(4):469-77.

8. Verbruggen S, Sy J, Arrivillaga A, Joosten K, van Goudoever J, Castillo L. Parenteral Amino Acid Intakes in Critically Ill Children: A Matter of Convenience. JPEN J Parenter Enteral Nutr 2010;34(3):329-40.

9. Derde S, Vanhorebeek I, Guiza F, Derese I, Gunst J, Fahrenkrog B, et al. Early Parenteral Nutrition Evokes a Phenotype of Autophagy Deficiency in Liver and Skeletal Muscle of Critically Ill Rabbits. Endocrinology 2012.

10. Levine B, Mizushima N, Virgin HW. Autophagy in immunity and inflammation. Nature 2011;469(7330):323-35.

11. Vanhorebeek I, Gunst J, Derde S, Derese I, Boussemaere M, Guiza F, et al. Insufficient activation of autophagy allows cellular damage to accumulate in critically ill patients. J Clin Endocrinol Metab 2011;96(4):E633-45.

12. Vlasselaers D, Milants I, Desmet L, Wouters PJ, Vanhorebeek I, van den Heuvel I, et al. Intensive insulin therapy for patients in paediatric intensive care: a prospective, randomised controlled study. Lancet 2009;373(9663):547-56.

13. WHO 'Prevention of hospital-acquired infections; a practical guide, 2nd edition' *www.who.int/csr/.../whocdscsreph200212.pdf*

14. Vlasselaers D, Mesotten D, Langouche L, Vanhorebeek I, van den Heuvel I, Milants I, et al. Tight glycemic control protects the myocardium and reduces inflammation in neonatal heart surgery. Ann Thorac Surg 2010;90(1):22-9.

15. Verhoeven JJ, Brand JB, van de Polder MM, Joosten KF. Management of hyperglycemia in the pediatric intensive care unit; implementation of a glucose control protocol. Pediatr Crit Care Med 2009;10(6):648-52.

16. WHO. Management of severe malnutrition: a manual for physicians and other senior health workers. Geneva: World Health Organization 1999.

17. Ellger B, Debaveye Y, Vanhorebeek I, Langouche L, Giulietti A, Van Etten E, et al. Survival benefits of intensive insulin therapy in critical illness: impact of maintaining normoglycemia versus glycemia-independent actions of insulin. Diabetes 2006;55(4):1096-105.

18. Henckaerts L, Nielsen KR, Steffensen R, Van Steen K, Mathieu C, Giulietti A, et al. Polymorphisms in innate immunity genes predispose to bacteremia and death in the medical intensive care unit. Crit Care Med 2009;37(1):192-201, e1-3.

19. Derde S, Vanhorebeek I, Ververs EJ, Vanhees I, Darras VM, Van Herck E, et al. Increasing intravenous glucose load in the presence of normoglycemia: effect on outcome and metabolism in critically ill rabbits. Crit Care Med 2012;38(2):602-11.

20. Bjorkoy G, Lamark T, Pankiv S, Overvatn A, Brech A, Johansen T. Monitoring autophagic degradation of p62/SQSTM1. Methods Enzymol 2009;452:181-97.

21. Komatsu M, Waguri S, Ueno T, Iwata J, Murata S, Tanida I, et al. Impairment of starvation-induced and constitutive autophagy in Atg7-deficient mice. J Cell Biol 2005;169(3):425-34.

22. Masiero E, Agatea L, Mammucari C, Blaauw B, Loro E, Komatsu M, et al. Autophagy is required to maintain muscle mass. Cell Metab 2009;10(6):507-15.

23. Van den Berghe GH. Acute and prolonged critical illness are two distinct neuroendocrine paradigms. Verh K Acad Geneeskd Belg 1998;60(6):487-518; discussion 518-20.

24. Langer M, Modi BP, Agus M. Adrenal insufficiency in the critically ill neonate and child. Curr Opin Pediatr 2006;18(4):448-53.

25. Gielen M, Mesotten D, Brugts M, Coopmans W, Van Herck E, Vanhorebeek I, et al. Effect of intensive insulin therapy on the somatotropic axis of critically ill children. J Clin Endocrinol Metab 2011;96(8):2558-66.

26. Cuzzola A, Petri A, Mazzini F, Salvadori P. Application of hyphenated mass spectrometry techniques for the analysis of urinary free glucocorticoids. Rapid Commun Mass Spectrom 2009;23(18):2975-82.

27. Hakkaart-van Roijen L et al. Handleiding voor kostenonderzoek, methoden en standaard kostprijzen voor economische evaluaties in de gezondheidszorg. CVZ. 2010

CCMO

CENTRALE
COMMISSIE
MENSGEBONDEN
ONDERZOEK

Erasmus MC –Sophia afdeling Intensive Care kindergeneeskunde
Tav Dr SCAT Verbruggen, kamer Sp 3140
Postbus 2060
3000 CB Rotterdam

29 mei 2012
uw kenmerk: n.v.t., ons kenmerk: CCMO12.0722/MK/cb/38772

Besluit NL38772.000.12

Geachte heer Verbruggen,

Hierbij zend ik u het besluit van de CCMO inzake het onderzoeksprotocol getiteld 'Paediatric Early versus late PArenteral Nutrition in Criti-cal illness (PEPaNIC)' (NL38772.000.12).

De CCMO verleent haar goedkeuring aan genoemd onderzoek. Voor de overwegingen bij het besluit verwijs ik u naar het bijgevoegde oordeel.
Vanaf 1 januari 2010 zijn indieners van investigator initiated onderzoek verplicht ernstige (bijwerkingen en) voorvallen (SAE's) te melden via ToetsingOnline (voor verdere details verwijs ik u naar de website van de CCMO).

De CCMO wijst u op de verplichtingen, die voortvloeien uit de WMO en de aanverwante regelgeving, waarvan een overzicht is opgenomen in de bijlage behorend bij dit besluit.

Ik hoop u hiermee naar behoren te hebben geïnformeerd.

Hoogachtend,

Namens de Centrale Commissie Mensgebonden Onderzoek

postadres:
Postbus 16302
2500 BH Den Haag

bezoekadres:
Parnassusplein 5
2511 VX Den Haag

tel: (070) 340 6700
fax: (070) 340 6737
ccmo@ccmo.nl
www.ccmo.nl

COMMISSIE MEDISCHE ETHIEK VAN DE UNIVERSITAIRE ZIEKENHUIZEN KULEUVEN
U.Z. GASTHUISBERG E330
HERESTRAAT 49
B-3000 LEUVEN (BELGIUM)

KATHOLIEKE
UNIVERSITEIT
LEUVEN

Aan Prof. G. Van den Berghe
Intensieve Geneeskunde, UZ Leuven

ONS KENMERK ML8052
LEUVEN, 30 maart 2012

Impact of early parenteral nutrition completing enteral nutrition in paediatric critically ill patients.PEPaNIC

EudraCT Nummer 2012-000811-10

S54127

DEFINITIEF GUNSTIG ADVIES

Geachte Collega,

De Commissie Medische Ethiek van de Universitaire Ziekenhuizen K.U.Leuven heeft vermeld protocol onderzocht en besproken op haar vergadering van 2 maart 2012.

Na inzage van de bijkomende informatie en/of aangepaste documenten met betrekking tot vermeld dossier is de Commissie van oordeel dat de voorgestelde studie, zoals beschreven in het protocol, wetenschappelijk relevant en ethisch verantwoord is. Ze verleent dan ook een gunstig advies over deze studie.

Bij het beoordelen van dit dossier werd rekening gehouden met de documenten en informatie gerelateerd aan deze studie, ingediend op 22 februari 2012 en 27 maart 2012.

Dit gunstig advies betreft:

- *Protocol & protocol gerelateerde documenten : versie 2 dd 27/03/2012*
- *Patiëntendocumenten versie 2 dd 27/03/2012*
- *IB SMOFlipid 200 mg/ml, emulsie voor infusie versie 03/2011 ; Vamin 18 gN Electrolyte Free, oplossing voor infusie versie 06/2008 ; Vaminolact, oplossing voor infusie versie 06/2008*

De Commissie bevestigt dat ze werkt in overeenstemming met de ICH-GCP principes (International Conference on Harmonization Guidelines on Good Clinical Practice) en met de van toepassing zijnde wetten en regelgeving.

De Commissie bevestigt dat in geval van belangenconflict, de betrokken leden niet deelnemen aan de besluitvorming omtrent de studie.

Een ledenlijst wordt bijgevoegd.

De opdrachtgever is verantwoordelijk voor de conformiteit van de anderstalige documenten met de Nederlandstalige documenten.

SECRETARIAAT: M. LEYS N. OPDEKAMP D. VAN MOLL M. VERBEECK H. HUYGHE
Tel +32 16 34 86 00 Fax +32 16 34 86 01 ec@uzleuven.be ec-submission@uzleuven.be www.uzleuven.be/ec

ONS KENMERK ML8052
UW KENMERK
LEUVEN , 30 maart 2012

Aandachtspunten:

Indien er een Clinical Trial Agreement is, kan de studie in ons centrum pas aangevat worden wanneer dit Clinical Trial Agreement goedgekeurd en ondertekend is door de gedelegeerd bestuurder van UZ Leuven.

Studies met geneesmiddelen en sommige studies met "medische hulpmiddelen" dienen door de opdrachtgever aangemeld te worden bij het FAGG.
Studies met geneesmiddelen mogen slechts aanvangen op voorwaarde dat de minister (FAGG) geen bezwaren heeft kenbaar gemaakt binnen de wettelijke termijnen zoals beschreven in art.13 van de Belgische wet van 7/5/2004 inzake experimenten op de menselijke persoon.
Voor bepaalde studies met medische hulpmiddelen gelden eveneens wettelijke termijnen (zie KB van 17/3/2009).
Voor meer informatie hieromtrent verwijzen we naar de website van het FAGG www.fagg-afmps.be

Gelieve ook rekening te houden met de regelgeving van het ziekenhuis betreffende weefselbeheer.

Dit gunstig advies van de Commissie houdt niet in dat zij de verantwoordelijkheid voor de geplande studie op zich neemt. U blijft hiervoor dus zelf verantwoordelijk. Bovendien dient U er over te waken dat uw mening als betrokken onderzoeker wordt weergegeven in publicaties, rapporten voor de overheid enz., die het resultaat zijn van dit onderzoek.

U wordt eraan herinnerd dat bij klinische studies iedere door U waargenomen ernstige verwikkeling onmiddellijk zowel aan de opdrachtgever (desgevallend de producent) als aan de commissie medische ethiek moet worden gemeld, ook al is het oorzakelijke verband met de studie onduidelijk.

Indien de studie niet binnen het jaar beëindigd is, vereist de ICH-GCP dat een jaarlijks vorderingsrapport aan de commissie wordt bezorgd.

Tenslotte verzoeken wij U ons mee te delen indien een studie niet wordt aangevat, of wanneer ze wordt afgesloten of vroegtijdig onderbroken (met opgave van eventuele reden). Gelieve het (al dan niet vroegtijdig) stopzetten van een studie binnen de door de wet vastgelegde termijnen mee te delen en een Clinical Study Report aan de Commissie te bezorgen.

Met de meeste hoogachting,

Prof. Dr. Walter VAN DEN BOGAERT
Voorzitter Commissie Medische Ethiek
UZ K.U.LEUVEN

Prof. Dr. W. Van den Bogaert
Voorzitter
Commissie Medische Ethiek van de UZ K.U.Leuven

Cc : **FAGG** (Federaal Agentschap voor Geneesmiddelen en Gezondheidsproducten)
Departement R&D Eurostation, blok 2
Victor Hortaplein 40, bus 40 B-1060 Brussel

Clinical Trial Center (CTC), UZ Leuven, Campus Gasthuisberg

SECRETARIAAT: **M. LEYS** **N. OPDEKAMP** **D. VAN MOLL** **M. VERBEECK** **H. HUYGHE**
Tel +32 16 34 86 00 Fax +32 16 34 86 01 ec@uzleuven.be ec-submission@uzleuven.be www.uzleuven.be/ec

COMMISSIE MEDISCHE ETHIEK VAN DE UNIVERSITAIRE ZIEKENHUIZEN K.U.LEUVEN
TOETSINGSCOMMISSIE
UZ GASTHUISBERG E330
HERESTRAAT 49
3000 LEUVEN BELGIUM

KATHOLIEKE
UNIVERSITEIT
LEUVEN

Ledenlijst Commissie Medische Ethiek/Toetsingscommissie (OG032)
vanaf 22 december 2011 tot op heden
List of Members Ethics Committee/IRB (OG032)
from December 22nd, 2011 until present

Prof. Walter Van den Bogaert, M.D.	Chairman (M)	Radiotherapy-Oncology
Dr. Johan Wildiers, M.D.	Vice-Chairman (M)	Medical Oncology
Dr. Sabine Graux, M.D.	Secretary (F)	Physician
Dr. Sonja Haesendonck, M.D.	Secretary (F)	Physician
Prof. Xavier Bossuyt, M.D.	Member (M)	Immunology
Prof. D. Bullens, M.D.	Member (F)	Paediatrics
Prof. Willem Daenen, M.D.	Member (M)	Cardiac Surgery
Dr. Lut De Groote, M.D.	External Member (F)	General Practitioner
Prof. Jan de Hoon, M.D.	Member (M)	Clinical Pharmacology
Prof. Ivo De Wever, M.D.	Member (M)	Oncology
De heer Filip Gybels	Member (M)	Head Nurse
Prof. Walter Heyns, M.D.	Member (M)	Experimental Medicine
Dr. José Thomas, M.D.	Member (M)	Medical Oncology
Dr. Ben Van Calster	Member (M)	Statistics
Prof. Jan Van Hemelrijck, M.D.	Member (M)	Anesthesiology
Prof. Raymond Verhaeghe, M.D.	Member (M)	Cardiology
Prof. Guido Verhoeven, M.D.	Member (M)	Experimental Medicine
Mrs. Christine Mathieu, Law	Member (F)	Medical Legislation

(M) = Male (F) = Female

De Commissie voor Medische Ethiek volgt de voorschriften van ICH Good Clinical Practice en de lokale wettelijke bepalingen terzake (wet van 7 mei 2004 inzake experimenten op de menselijke persoon en bijbehorende KB's en programmawet)

The Ethics Committee operates according to ICH Good Clinical Practice and local applicable regulations.

Approval Form
Date:April 23, 2013
Principal Investigator:Ari Joffe
Study ID:Pro00038098
Study Title:
Impact of Early Parenteral Nutrition Completing Enteral Nutrition in Paediatric Critically Ill Patients
Approval Expiry Date:April 22, 2014
Approved Consent Document:
Approval DateApproved Document
4/23/2013PEPaNIC Information and Consent Revised
Funding/Sponsor:IWT (Agency for Innovation by Science and Technology) TBM project 110685
Thank you for responding to all matters arising from your presentation of this study at the April 19, 2013 Health Research Ethics Board - Biomedical Panel meeting. There are no outstanding issues, and the study is approved. The Protocol (version number 3, 27-02-2013) and the consent (undated link above) are both approved.
The Health Research Ethics Board assessed all matters required by section 50(1)(a) of the Health Information Act. Subject consent for access to identifiable health information, will be obtained by someone involved in the clinical care of the participant, and appropriate procedures for such consent have been approved by the HREB - Biomedical Panel. In order to comply with the Health Information Act, a copy of the approval form is being sent to the Office of the Information and Privacy Commissioner.
A renewal report must be submitted next year prior to the expiry of this approval if your study still requires ethics approval. If you do not renew on or before the renewal expiry date (April 22, 2014), you will have to re-submit an ethics application.
The membership of the Health Research Ethics Board - Biomedical Panel complies with the membership requirements for research ethics boards as defined in Division 5 of the Food and Drug Regulations and the Tri-Council Policy Statement. The HREB - Biomedical Panel carries out its functions in a manner consistent with Good Clinical Practices.
Approval by the Health Research Ethics Board does not encompass authorization to access the patients, staff or resources of Alberta Health Services or other local health care institutions for the purposes of the research. Enquiries regarding Alberta Health Services administrative approval, and operational approval for areas impacted by the research, should be directed to the Alberta Health Services Research Administration office, #1800 College Plaza, phone (780) 407-6041.
Sincerely,
S.K.M. Kimber, MD, FRCPC
Chair, HREB Biomedical
Note: This correspondence includes an electronic signature (validation and approval via an online system).

ontvangen op 19/3/13

FACULTEIT GENEESKUNDE
COMMISSIE VOOR MEDISCHE ETHIEK/KLINISCH ONDERZOEK
UZ GASTHUISBERG E330
HERESTRAAT 49
3000 LEUVEN (BELGIUM)

KATHOLIEKE
UNIVERSITEIT
LEUVEN

Prof. G. Van Den Berghe
Intensieve Zorgen - UZ Leuven - Gasthuisberg
Fax: 44015

ONS KENMERK: ML8052 (BD-nr 1 dd. 7 februari 2013)
LEUVEN: 7 maart 2013

Impact of early parenteral nutrition completing enteral nutrition in paediatric critically ill patients.PEPaNIC

Eudract: 2012-000811-10
StudieReferentie: S54127

AMENDEMENT/BIJKOMENDE STUDIEDOCUMENTEN

DEFINITIEF GUNSTIG ADVIES

Geachte collega,

De Commissie Medische Ethiek van de Universitaire Ziekenhuizen KU Leuven heeft vermeld protocol initieel goedgekeurd op 30 maart 2012.

Met betrekking tot vermeld protocol werden bijkomende documenten ingediend bij de Commissie Medische Ethiek van de Universitaire Ziekenhuizen KU Leuven.

Bij het beoordelen van dit amendement werd rekening gehouden met alle aan dit amendement gerelateerde documenten die ingediend werden op 7 februari 2013.

Het amendement werd goedgekeurd op 27 februari 2013.

Dit gunstig advies betreft o.m.:

- Amendement op Protocol
  Protocol versie 3 dd. 05-Feb-2013
- Questionnaires
  - vragenlijst 0 - 3 jr.
  - vragenlijst 4 - 18 jr.
- Amendement op ICF
  - ICF versie 3 dd. 17-Dec-2012 (Nl)
  - ICF versie 1 dd. 05-Feb-2013 (E, Fr)

De Commissie bevestigt dat ze werkt in overeenstemming met de ICH-GCP principes (International Conference on Harmonization Guidelines on Good Clinical Practice) en met de geldende wetten en regelgeving.

SECRETARIAAT: Tel. +32-16 34 86 00 Fax. +32-16 34 86 01 ec@uzleuven.be www.uzleuven.be/ec

ONS KENMERK: ML8052
LEUVEN: 7 maart 2013

De Commissie bevestigt dat in geval van belangenconflict, de betrokken leden niet deelnemen aan de besluitvorming omtrent het amendement.

Een ledenlijst wordt bijgevoegd.

De opdrachtgever is verantwoordelijk voor de conformiteit van de anderstalige documenten met de Nederlandstalige documenten.

Aandachtspunten:

Ieder substantieel protocol amendement moet ook worden aangemeld bij het Clinical Trial Center, UZ Leuven (www.uzleuven.be/ctc). Zo het amendement een wijziging van het clinical trial agreement inhoudt, dan kan dit amendement pas geïmplementeerd worden na ondertekening van het clinical trial agreement door de gedelegeerd bestuurder van UZ Leuven.

Bepaalde amendementen betreffende studies met geneesmiddelen en sommige studies met 'medische hulpmiddelen' dienen door de opdrachtgever aangemeld worden bij FAGG. Het desbetreffende studie amendement kan slecht geïmplementeerd worden op voorwaarde dat de minister (FAGG) geen bezwaren heeft kenbaar gemaakt binnen de wettelijke termijnen zoals beschreven in art. 13 van de Belgische wet van 7/5/2004 inzake experimenten op de menselijke persoon.

Voor bepaalde studies met medische hulpmiddelen gelden eveneens wettelijke termijnen (zie KB van 17/3/2009). Voor meer informatie hieromtrent verwijzen we naar de website van FAGG www.fagg-afmps.be.

Met de meeste hoogachting,

Prof. Dr. Walter VAN DEN BOGAERT
Voorzitter Commissie Medische Ethiek
UZ K.U.LEUVEN

Prof. dr. W. Van den Bogaert
Voorzitter Commissie Medische Ethiek van de UZ KU Leuven

Cc. aan:

FAGG (Federaal Agentschap voor Geneesmiddelen en Gezondheidsproducten) - Departement R&D;
Victor Hortaplein 40, bus 40 1060 Brussel
UZ Leuven - Gasthuisberg - Clinical Trial Center

ONS KENMERK: ML8052
LEUVEN: 7 maart 2013

Ledenlijst Commissie Medische Ethiek UZ KU Leuven

Prof. dr. Walter Van den Bogaert (Chairman) Radiotherapy/Oncology

Dr. Johan Wildiers (Vice-Chairman) Medical Oncology

Dr. Sabine Graux (Secretary) Physician

Dr. Sonja Haesendonck (Secretary) Physician

Prof. dr. Xavier Bossuyt (Member) Immunology

Prof. dr. Dominique Bullens (Member) Paediatrics

Prof. dr. Willem Daenen (Member) Cardiac surgery

Dr. Lut De Groote (External Member) General Practitioner

Prof. dr. Jan de Hoon (Member) Clinical Pharmacology

Prof. dr. Ivo De Wever (Member) Surgical Oncology

De heer Filip Gybels (Member) Head Nurse

Mevr. Christine Mathieu (Member) Medical Legislation

Dr. José Thomas (Member) Medical Oncology

Prof. J.R. Thomas (Member) Clinical Pharmacology

Prof. Ben Van Calster (Member) Statistician

Prof. dr. Jan Van Hemelrijck (Member) Anesthesiology

Prof. dr. Raymond Verhaeghe (Member) Cardiology

Prof. dr. Guido Verhoeven (Member) Experimental Medicine

SECRETARIAAT: Tel. +32-16 34 86 00 Fax. +32-16 34 86 01 ec@uzleuven.be www.uzleuven.be/ec

Final protocol PEPaNIC study

Paediatric Early versus late

Parenteral Nutrition In Critical illness

- PEPaNIC

(December 2013)

PROTOCOL TITLE **'Paediatric Early versus late Parenteral Nutrition In Critical illness'**

**Protocol ID**	**PEPaNIC - NL**
**Short title**	**PEPaNIC**
**Version**	**5**
**Date**	**17-12-2013**
**Coordinating investigator/project leader**	***Prof. Dr. G Van Den Berghe, Katholieke Universiteit, Leuven, Belgium***
**Principal investigator(s) (in Dutch: hoofdonderzoeker/uitvoerder)**<br>***Multicenter research: per site***	***Erasmus MC – Sophia Children's Hospital***<br>***Prof. Dr. D Tibboel***<br>***Dr. SCAT Verbruggen***<br>***Dr. KFM Joosten***<br>***Drs. D. Kerklaan***<br>***Katholieke Universiteit, Leuven, Belgium***<br>***Prof. Dr. G Van Den Berghe***<br>***Prof. Dr. D. Mesotten***<br>***Prof. I Vanhorenbeek***<br>***Dr. L Desmet***<br>***Dr. T Fivez***<br>***Stollery Children's Hospital Edmonton, Canada***<br>***Dr. A. Joffe***<br>***Dr. G. G. Guerra***<br>***Dr. B. Larsen***
**Sponsor (in Dutch: verrichter/opdrachtgever)**	***Investigator Initiated***
**Independent physician(s)**	***Prof. Dr. IKM Reiss, Neonatology, Erasmus MC – Sophia Children's Hospital***
**Laboratory sites <*if applicable*>**	**Laboratory Paediatrics,**<br>**Erasmus MC –Sophia, Rotterdam**

	Laboratory Intensive Care Medicine, University Hospital Leuven, Belgium
Pharmacy <*if applicable*>	*Erasmus MC – Sophia (Rotterdam site)*<br>*University Hospital Leuven (Leuven site)*<br>*Stollery Children's Hospital Edmonton*

PROTOCOL SIGNATURE SHEET

Name	Signature	Date
For non-commercial research,<br>Head of Department:	Prof. Dr. D. Tibboel<br>Head of department, Pediatric ICU	
Coordinating Investigator/Project leader<br><br>Principal Investigator:	Dr. KFM Joosten<br><br>Dr. SCAT Verbruggen	

TABLE OF CONTENTS

1. INTRODUCTION AND RATIONALE 9
2. OBJECTIVES 11
3. STUDY DESIGN 13
4. STUDY POPULATION 16
4.1 Population Base 16
4.2 Inclusion criteria 16
4.3 Exclusion criteria 16
4.4 Sample size calculation 16
5. TREATMENT OF SUBJECTS 18
5.1 Investigational treatment 18
5.2 Use of co-intervention (if applicable) 23
5.3 Escape medication (if applicable) 23
6. INVESTIGATIONAL MEDICINAL PRODUCT 23
7. METHODS 24
7.1 Study parameters/endpoints 24
7.1.1 Main study parameter/endpoint 24
7.1.2 Secondary study parameters/endpoints 24
7.1.3 Other study parameters 29
7.2 Randomisation, blinding and treatment allocation 30
7.3 Study procedures30
7.4 Withdrawal of individual subjects 33
7.4.1 Specific criteria for withdrawal (if applicable) 33
7.5 Replacement of individual subjects after withdrawal 34
7.6 Follow-up of subjects withdrawn from treatment 34
7.7 Premature termination of the study 34
8. SAFETY REPORTING 35
8.1 Section 10 WMO event 37
8.2 Adverse and serious adverse events 37

8.2.1 Suspected unexpected serious adverse reactions (SUSAR) 38
8.2.2 Annual safety report 38
8.3 Follow-up of adverse events 39
8.4 Data Safety Monitoring Board (DSMB) 39
9. STATISTICAL ANALYSIS 40
9.1 Descriptive statistics 40
9.2 Univariate analysis 41
9.3 Multivariate analysis 41
9.4 Interim analysis 43
10. ETHICAL CONSIDERATIONS 44
10.1 Regulation statement 44
10.2 Recruitment and consent 44
10.3 Objection by minors or incapacitated subjects (if applicable) 44
10.4 Benefits and risks assessment, group relatedness 45
10.5 Compensation for injury 45
10.6 Incentives 45
11. ADMINISTRATIVE ASPECTS AND PUBLICATION 46
11.1 Handling and storage of data and documents 46
11.2 Amendments 46
11.3 Annual progress report 46
11.4 End of study report 46
11.5 Public disclosure and publication policy 47
12. REFERENCES 48

LIST OF ABBREVIATIONS AND RELEVANT DEFINITIONS

ABR	ABR form, General Assessment and Registration form, is the application form that is required for submission to the accredited Ethics Committee (In Dutch, ABR = Algemene Beoordeling en Registratie)
AE	Adverse Event
AR	Adverse Reaction
CA	Competent Authority
CCMO	Central Committee on Research Involving Human Subjects; in Dutch: Centrale Commissie Mensgebonden Onderzoek
CV	Curriculum Vitae
DSMB	Data Safety Monitoring Board
EU	European Union
EudraCT	European drug regulatory affairs Clinical Trials
GCP	Good Clinical Practice
IB	Investigator's Brochure
IC	Informed Consent
IMP	Investigational Medicinal Product
IMPD	Investigational Medicinal Product Dossier
METC	Medical research ethics committee (MREC); in Dutch: medisch ethische toetsing commissie (METC)
(S)AE	(Serious) Adverse Event
SPC	Summary of Product Characteristics (in Dutch: officiële productinfomatie IB1-tekst)
Sponsor	The sponsor is the party that commissions the organisation or performance of the research, for example a pharmaceutical<br><br>company, academic hospital, scientific organisation or investigator. A party that provides funding for a study but does not commission it is not regarded as the sponsor, but referred to as a subsidising party.
SUSAR	Suspected Unexpected Serious Adverse Reaction

Wbp Personal Data Protection Act (in Dutch: Wet Bescherming Persoonsgevens)

WMO Medical Research Involving Human Subjects Act (in Dutch: Wet Medisch-wetenschappelijk Onderzoek met Mensen

SUMMARY

Rationale: It has recently been shown in a large randomized study in adult ICU patients that the early PN caused an increase of morbidity (infections and increased length of stay on the ICU). Such a study is warranted also in critically ill children of different age groups, as PN has been associated with increased incidence of complications, such as (line) infections, hyperglycemia and hepatic steatosis. It has not been studied whether early PN, when enteral feeding is insufficient, influences the outcome of critically ill children.

Objective: The ultimate aim of this project is to answer the question whether insufficient EN should or should not be supplemented with PN early in the disease course of critical illness in children. The scientific objectives of the study are the following: 1) Performing the first well-designed and sufficiently powered, multicentre RCT to test the effects of early PN supplementation on the health of critically ill children. 2) Performing an economic evaluation study, investigating the costs and cost-effectiveness, during hospitalisation of early versus late PN supplementation. 3) Starting to unravel potential mechanisms that underlie any difference in acute and long term outcome with early or late PN supplementation by studying metabolic, endocrine, inflammatory and (epi.)genetic markers.

Study design: This study is a large, three-centre, non-blinded, randomized, controlled study.

Consecutive patients are randomly assigned to one of the two treatment study groups using a digital system with central, computerised randomisation. Randomisation – in a one to one allocation ratio - is performed per centre and using permuted blocks of 10 per diagnostic stratum. The block size remains unknown to bed-side physicians and nurses, responsible for patient recruitment and therapy assignment.

Study population: Critically ill children (0 – 18 yrs), with nutritional risk score (STRONGkids® see appendix) 2 or more, who are not able to take oral nutrition and are expected to stay in PICU for more than 24h are eligible for inclusion. Exclusion criteria are a “do not resuscitate” code at the time of PICU admission, expected death within 12 hours, re-admission to the PICU after previously being randomised to the PEPaNIC trial, transfer from another PICU after a stay of more than seven days, ketoacidotic or hyperosmolar coma on admission or inborn metabolic diseases requiring specific diet, premature newborns (<37 weeks gestational age), patients on Total Parenteral Nutrition for >7 days prior to inclusion, short bowel syndrome or other conditions which required home-PN.

Intervention: The “early PN” strategy will be the nutritional management currently applied in the participating centres, acting as “control”. The “late PN” strategy comprises initiation of this strategy only after day 7 in PICU, patients will receive a mixture of Glucose 5% and NaCl 0.9% at, respectively, 60% and 40% of the total flow rate that is required to obtain standard optimal hydration taking into account the volume of EN that is being delivered. If enteral feeding of at least 80% of the calculated calories is not possible after 7 days in ICU, PN, as specified above, is initiated on day 8.

Main study parameters/endpoints: We will compare “early PN” with “late PN” in paediatric ICU patients at risk of developing malnutrition in the ICU. The primary focus of the PEPaNIC study is clinical outcome, more specifically the acquisition of new ICU infections, the dependency on intensive medical care and convalescence from critical illness.

Nature and extent of the burden and risks associated with participation, benefit and group relatedness: The burden is expected to be minimal as it will only entail additional blood draws, which will be taken from clinical lines or in addition to pricks for clinical purpose. The long-term follow up will be held as part of already organised follow-up outdoor clinics and which are developed to help children and their parents to recover physically, emotionally and socially after ICU admissions. For mechanistic and exploratory studies, muscle strength testing and an ultrasound evaluation of the skeletal muscle and adipose tissue compartments will be performed. Furthermore, as far as practically feasible, every patient is approached just prior to hospital discharge, to complete a validated, semi-structured child health questionnaire (Functional Independence Measure (WEEFIM), Health Utilities Index (HUI) and Child Health Questionnaire (CHQ)).

The risk in participating to the study and being randomized to the "late PN' group are negligible, and specifically compass an increased risk of developing hypoglycemia and/or underfeeding. However, safety measures will be taken to further decrease these risks.

GROUP relatedness: This study was already performed in adult ICU patients. However, the results of this study should NOT be translated one-on-one to the pediatric patient, as critically ill children of different age groups have different metabolic and nutritional issues. Therefore, this study deserves to be repeated in our population of critically ill children.

13. INTRODUCTION AND RATIONALE

It has recently been shown that withholding parenteral nutrition (PN.) during the first week of critical illness (Late PN) is beneficial in comparison with the early supplementation of insufficient enteral nutrition (EN) with PN (Early PN) in critically ill adults (1). The benefits of Late PN mainly encompassed a decrease in the incidence of new infections in the ICU, a shortening of the stay in the ICU and hospital, and a reduction in healthcare costs. These findings put pressure on the current guidelines by the European Society of Parenteral & Enteral Nutrition (ESPEN), which recommend the practice of Early PN in critically adults (2). These current medical practice and expert panels advocate the early supplementation by PN. This is based on the premise that PN is probably beneficial, and if not, harmless. This contrasts strongly with the findings of the study in adult critically ill patients where it has been shown that this early supplementation by PN is not providing benefit and is actually causing harm to patients by increasing the morbidity and delaying recovery.

The detrimental impact of the practice of Early PN in critically ill adults has alerted the clinical community that also the guidelines for nutritional strategy in paediatric critically ill patients are merely based on expert opinion. Not only is Early PN current practice in most paediatric ICUs, it is also often more aggressively promoted (3, 4). This is further evidenced by the presence of national governmental healthcare programs that include the success rate for reaching nutritional goals as a quality benchmark (5). Therefore critically ill children may be exposed to risk by the aggressive feeding protocols, which were previously deemed innocent or even beneficial. We and others have shown recently that current aggressive feeding protocols with early high protein and lipid intakes, not only fail to achieve anabolism, but have potential negative side effects, such as increased risk of developing hyperglycemia, dyslipidemia and insulin resistance (6, 7). Furthermore, there is currently no evidence of beneficence of early supplementation of parenteral nutrition, although this is practiced in multiple PICU's according current guidelines. PN has been associated with increased incidence of complications, such as (line) infections, hyperglycemia and hepatic steatosis. Furthermore, it is well recognized that the quality of current PN solutions lack sufficient scientific basis and are potentially harmful (8). Therefore, it is unclear whether an early supplementary intervention of parenteral nutrition to try and decrease catabolism in critically ill children outweighs the potential harm of such therapy. Furthermore, recent studies have suggested a negative role of early aggressive nutrition in the interference of autophagy of cellular damage in critically ill patients, a process that may protect against infectious diseases and hyperinflammation (9-11). Children are in the early phase of their life when the fundaments of metabolism are developing. Therefore, it cannot be excluded that the aggressive nutritional support given to critically ill children has repercussions on the long run, until later in their adult life. And thus, such a study deserves to be performed in a population of critically ill children.

In our study we aim to evaluate whether the current practice of Early PN in critically ill children really provides clinical benefits over a strategy of withholding PN during the first 7 days in the paediatric ICU. The evaluation will be done in a multicentre randomized controlled trial performed in three large expert tertiary referral paediatric ICUs. The study will be statistically sufficiently powered to detect differences in clinically relevant outcome variables (the rate of new infections and the length of stay in the ICU). The trial will also be able to detect a doubling or halving of the mortality rate with a respectable statistical power (details on statistical power are given in the description further on). The results of this randomized controlled trial will provide, for the first time, high quality evidence for practice guidelines for nutrition in critically ill children. It will answer the question whether Early PN is beneficial, harmful or neutral in comparison with the new therapy

of withholding PN during the first week of critical illness. In weighing this evidence for current nutritional practice, this study will take into account 1) the short-term as well as the long-term health effects of the feeding protocols, 2) costs, and cost-effectiveness and 3) some insights into the underlying mechanisms of the observed clinical differences.

Also, one of the goals of the PEPaNIC study is to look into the tolerance for enteral nutrition (EN). In critically ill children EN is preferred because it is more physiological, presents fewer complications, leads to a better outcome and is less expensive than PN. However, EN often fails in this patient group due to impaired gastrointestinal (GI) motility, digestion and absorption following ischemia, inflammation or starvation leading to intolerance to EN. To improve future EN tolerance in critically ill children we have to gain insight in parameters of influence in this complex process.

One of the paramaters is mucosal inflammation, which can be related to malabsorption, as seen in patients with inflammatory bowel disease (IBD). Intestinal epithelial cells play a role in initiating and regulating mucosal immune responses through the secretion of chemokines. Studies in patients with IBD showed that epithelial cells derived from the intestine produce significant amounts of pro and anti-inflammatory chemokines (CXCL-8, CXCL-9 and CXCL-10). Chemokine expression can be measured in buccal epithelium which can be used as a reflection of intestinal inflammation.

14. OBJECTIVES

The ultimate aim of this project is to answer the question whether insufficient EN should or should not be supplemented with PN early in the disease course of critical illness in children.

The scientific objectives of the study are divided into 3 main work-packages (WP 1-3) and are the following:

- WP1: Performing and completing the first well-designed and sufficiently powered, multicentre RCT to test the effects of early PN supplementation on the health of critically ill children. The short-term outcome analysis, as well as a substantial part of the long-term follow-up, will be completed within the time frame of the study.
- WP2: an economic evaluation, in which costs and cost-effectiveness will be investigated of early versus late PN supplementation during hospitalization.
- WP3: Starting to unravel potential mechanisms that underlie any difference in acute and long term outcome with early or late PN supplementation by studying metabolic, endocrine, inflammatory and (epi.)genetic markers in the blood of critically ill children included in the study.

Primary Objective:

The primary focus of the PEPaNIC study is clinical outcome, more specifically the acquisition of new ICU infections, the dependency on intensive medical care and convalescence from critical illness, this is part of WP1.

The primary efficacy endpoints for this RCT are the incidence of new infections and the time to discharge alive from ICU. We expect to see a decrease in secondary infections from 20% to 15%, which is based on a previous study in critically ill children in Leuven (12) and on retrospective data in critically ill children who were admitted for at least four days in the PICU, Rotterdam.

Number of patients with new infections and types of infection will be assessed by numbers and percentages, and the duration of any antibiotics therapy initiated after randomisation for those patients requiring antibiotics will be analysed by non-parametrical tests. As the time of ICU discharge to the regular ward may be affected by the availability of beds on the regular wards, which could induce bias, we a priori decided to analyse "time to discharge from ICU" as "time to ready for discharge from ICU". A patient is considered "ready for discharge" as soon as all clinical conditions for ICU discharge have been fulfilled (no longer in need for vital organ support). Time to discharge alive from ICU will be reported by Kaplan-Meier plots, with ICU non-survivors censored beyond the longest ICU stay of survivors and censoring time of patients still in the ICU at closing of the data file (90 days after last patient inclusion) over both treatment groups. The impact of "late PN" versus "early PN" will be analysed, with and without correction for age, nutritional status and risk categories and type and severity of illness, by Cox proportional hazard analysis. The distribution of the actual time to discharge from ICU will be reported for ICU-survivors and ICU-non-survivors separately. In view of the time window of the randomised intervention in ICU, also the proportion of patients staying beyond 8 days in ICU will be reported. Analyses on blood and urine for the primary clinical analyses include routine chemistry, haematology, and markers of inflammation. Further epigenetic, metabolic, endocrine and inflammatory measurements on stored samples in the context of mechanistic analyses will be

planned. All new infections of the lungs, the blood stream, the urinary tract and wounds are recorded by an infectious disease specialist. A nosocomial infection is defined as a localized or systemic condition 1) that results from adverse reaction to the presence of an infectious agent(s) or its toxin(s) and 2) that was not present or incubating at the time of admission to the PICU. The information used to determine the presence and classification of an infection should be a combination of clinical findings and results of laboratory and other tests. Definitions various types of proven and suspected infections will be used according to clinical, laboratory evidence and/or supportive data. WHO definitions for nosocomial infections will be used (13).

TABLE I. **Simplified criteria for surveillance of nosocomial infections**

Type of nosocomial infection	Simplified criteria
Surgical site infection	Any purulent discharge, abscess, or spreading cellulitis at the surgical site during the month after the operation
Urinary infection	Positive urine culture (1 or 2 species) with at least 10^5 bacteria/ml, with or without clinical symptoms
Respiratory infection	Respiratory symptoms with at least two of the following signs appearing during hospitalization: — cough — purulent sputum — new infiltrate on chest radiograph consistent with infection
Vascular catheter infection	Inflammation, lymphangitis or purulent discharge at the insertion site of the catheter
Septicaemia	Fever or rigours and at least one positive blood culture

WHO definitions for nosocomial infections (13).

Clinical evidence is derived from direct observation of the infection site or review of other pertinent sources of data, such as the patient's chart. Laboratory evidence includes results of cultures, antigen or antibody detection tests, or microscopic visualization. Supportive data are derived from other diagnostic studies, such as x-ray, ultrasound, computed tomography (CT) scan, magnetic resonance imaging (MRI), radiolabel scan, endoscopic procedure, biopsy, or needle aspiration. It will be recorded during the entire ICU period until discharge, death or until day 90 of admittance.

Bacteraemia is further classified by responsible pathogen and as catheter-related blood stream infection versus other bacteraemia.

To study the underlying physiological mechanisms of enteral nutrition (EN) intolerance of critically ill children and to identify markers of tolerance for EN to use in future intervention studies clinical signs of intolerance (gastric residual volume, diarrhoea, vomiting, abdominal swelling) will be recorded. Buccal swabs will be collected at several moments and will be stored for analysis (epithelial cell expression of chemokines).

Secondary Objectives:

WP2. Performing an economic evaluation study, investigating the effects of early versus late PN supplementation during hospitalization.

WP3. Starting to unravel potential mechanisms that underlie any difference in acute and long-term outcome with early or late PN supplementation by studying metabolic, endocrine, inflammatory and (epi.)genetic markers in the blood of critically ill children included in the study.

STUDY DESIGN

This study is a large, three-centre, non-blinded, randomized, controlled study. The study intervention will last for a maximum of 7 days per patient, and the entire study (final patient completed study) is anticipated to last 4 years, excluding the long-term follow-up, the health-economic analysis and the completion of the mechanistic studies. (See figure)

Consecutive patients are randomly assigned to one of the two treatment study groups using a digital system with central, computerised randomisation. Randomisation – in a one to one allocation ratio - is performed per centre and using permuted blocks of 10 per diagnostic stratum. The block size remains unknown to bed-side physicians and nurses, responsible for patient recruitment and therapy assignment.

The “early PN” strategy will be the nutritional management currently applied in the participating centres, acting as “control”.

The “late PN” strategy comprises initiation of this strategy only after day 7 in PICU.

Timeline of the entire study

Work package 1 (WP1)

The first milestone (M1) of the study will be the start of the large RCT in the centre of the

coordinating investigator/project leader in Leuven, closely followed by our center at Erasmus MC-Sophia. The study will continue in parallel in all three centers until patient number 720 will have left the ICU. At that time point, a safety interim analysis will be performed (milestone M2). Three possible scenarios are foreseen depending on the result of this analysis:

1. The interim analysis shows harm by “Late PN” and the clinical study will be prematurely stopped (“no go” decision).

2. The data safety monitoring board requests a new safety interim analysis because of reasonable doubt about the safety of “Late PN”. In that case, the study will continue in all three centers under very strict monitoring, and planning of additional safety interim analyses if necessary, until the end of the study (either when completed or when prematurely stopped).

3. The study will be continued in all three centers as planned, without further interim analyses, until all 1440 patients have been included.

In all three scenarios the end of the study will be milestone 3 (M3). Together with the finalization and cross-check of the clinical database and the writing of the corresponding manuscript of the short-term clinical outcome, this will constitute deliverable 1 (D1). Hence, D1 is guaranteed irrespective of premature interruption of the study. In view of the outcome results of the adult EPaNIC study, such premature interruption will rather be unlikely. The applicants have extensive experience in the management of large RCTs, including in critically ill children, and handling and monitoring of the corresponding large clinical databases. This invaluable experience will be key to successful accomplishment of this ambitious study.

Work package 2 (WP2)

The fourth milestone (M4) will be targeted for before the end of the clinical study and consists of the setup of appropriate template database structures for import of the data that will be needed for the health economy analysis. This will allow an efficient economic evaluation study shortly after completion of the clinical study and is planned within the 4 year time frame and of the study (Milestone M5 and deliverable D2).

In the unlikely event of premature interruption of the study, the health economic analysis will be performed after the safety interim analysis on which the decision to stop was based. Previous experience in detailed analyses of healthcare resource utilization in large randomized clinical studies, as well as close collaboration with the Finance & Accounting department and the pharmacies of all participating hospitals will guarantee successful and timely accomplishment of this deliverable.

Work package 3

The mechanistic studies will be coordinated by and performed in the Leuven Laboratory of Intensive Care Medicine and in the Erasmus MC. Part of the mechanistic analyses will be systematically performed throughout the study. More specifically, leukocyte function tests require the fresh isolation of leukocytes and hence will be performed in parallel with work package 1. Thus, these analyses will be completed at the end of the clinical study (milestone M6 and deliverable D3). The samples needed for the other mechanistic studies will be taken throughout the clinical study and as deliverable 4 (D4) will be available together with M3 and D1. The other mechanistic studies involving the investigation of autophagy, genetics and cytokines in relation to the inflammatory response, as well as of the neuroendocrine axes will be planned and started in the last phase after completion of the clinical study.

In (the unlikely) case the study would be terminated prematurely, the mechanistic studies will be even more important, as the clinical community will ask for due explanations for harm caused during the study. Therefore, in any case they will be started shortly after all clinical data have been collected.

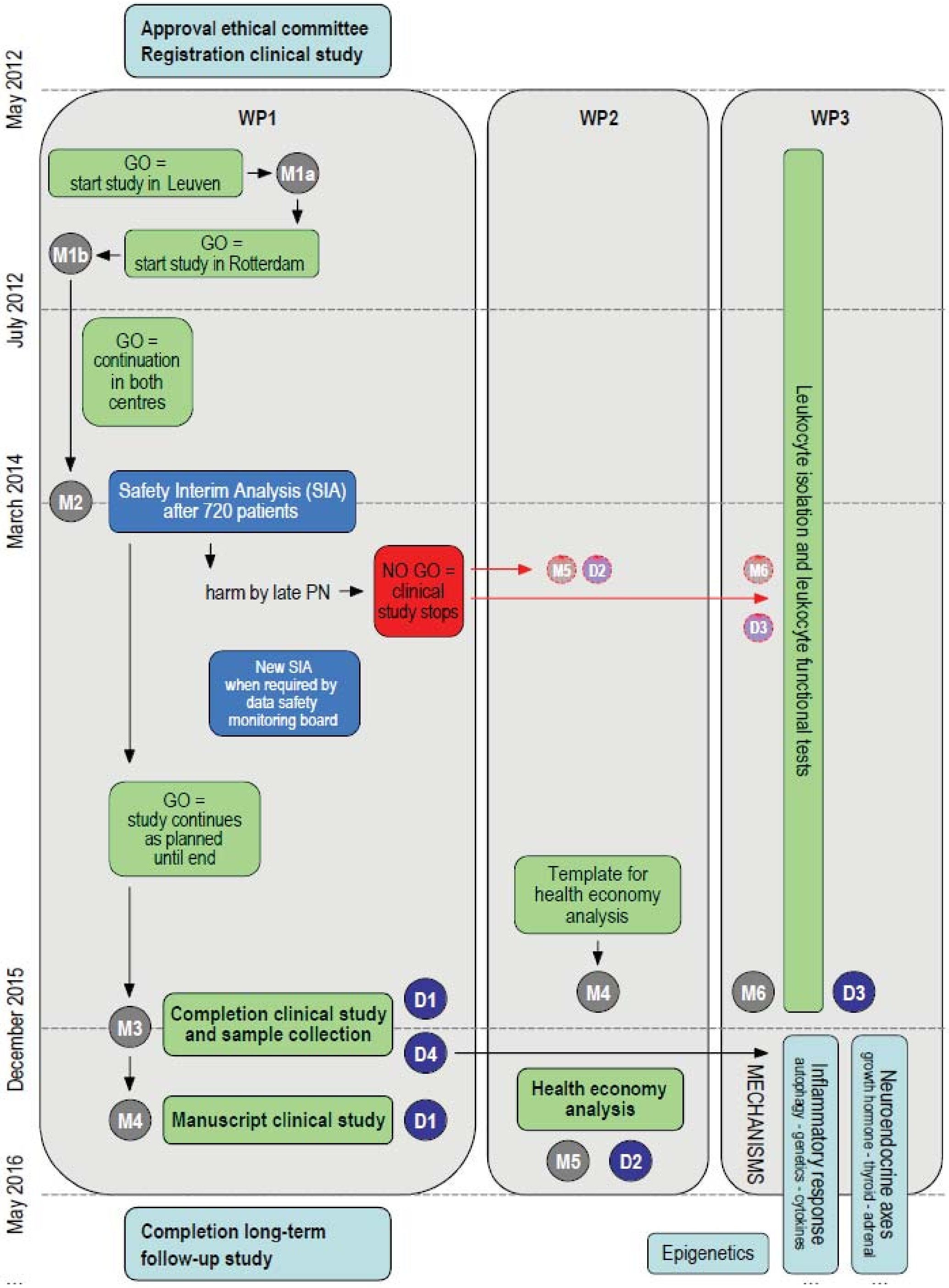

15. STUDY POPULATION

15.1 Population Base

The patient group of critically ill children (0 – 18 yrs), represents a significant proportion of the ICU population. In 2010, the 10-bed Leuven PICU admitted 527 critically ill children, the majority requiring artificial ventilation. 61% of the cases were admissions after cardiac surgery. Individual critically ill children had a median ICU stay of 3 days (inter-quartile range of 2-6 days) (mean 5.5 days). In the 35-bed PICU of the University of Rotterdam 1700 critically ill children were admitted in 2010 of which 600 (35.3%) were ventilated. Patients stayed in the PICU for a median of 2 days (mean 7.8 days). We anticipate an inclusion rate of 200-250 patients per centre per year.

15.2 Inclusion criteria

All critically ill children admitted to the participating PICUs are evaluated for nutritional risk and eligibility for inclusion in this study. All critically ill children, with nutritional risk score (STRONGkids® see appendix) 2 or more, who are not able to take oral nutrition and are expected to stay in PICU for more than 24h are eligible for inclusion.

15.3 Exclusion criteria

Exclusion criteria are a “do not resuscitate” code at the time of PICU admission, expected death within 12 hours, re-admission to the PICU after previously being participating in the PEPaNIC trial (except when < 48 hours after the initial discharge and still in the intervention window of the first 7 days), transfer from another PICU after a stay of more than seven days, ketoacidotic or hyperosmolar coma on admission or inborn metabolic diseases requiring specific diet, premature newborns (<37 weeks gestational age), patients on Total Parenteral Nutrition for >7 days prior to inclusion, short bowel syndrome or other conditions which required home-PN.

15.4 Sample size calculation

The sample size (N=1440, 720 per arm) is calculated in order to detect, with at least 80% power (one-tailed; the two-tailed power is 70%) and 95% certainty, a reduction in PICU infections from 20% to 15% and, with at least 90% power (two-tailed) and 95% certainty, a reduction in mean duration of stay in PICU of 1 day. With this sample size, and for safety reasons, also any substantial impact on mortality (increase or decrease with an absolute +/- 2%, although unexpected taken the adult data), can be excluded with a power of around 62-75% depending on the true mortality in the total population. We plan to calculate the true power of the study for detection of any eventual smaller differences in these outcomes.

16. TREATMENT OF SUBJECTS

16.1 Investigational treatment

The treatment, or intervention, in our study is best described as withholding PN in the early course of disease to reach currently recommended energy and protein intakes, when enteral delivery of these intakes is insufficient. In both allocation groups of the study, patients will be evaluated on a day to day basis to see which amount of enteral intake can be provided. The "early PN" group will receive current standard nutritional practice, which prescribes that PN should be added to EN as soon as possible in the course of disease, to achieve energy and protein goals. In the "late PN" group, we will wait to add PN, when EN is insufficient, until day 7.

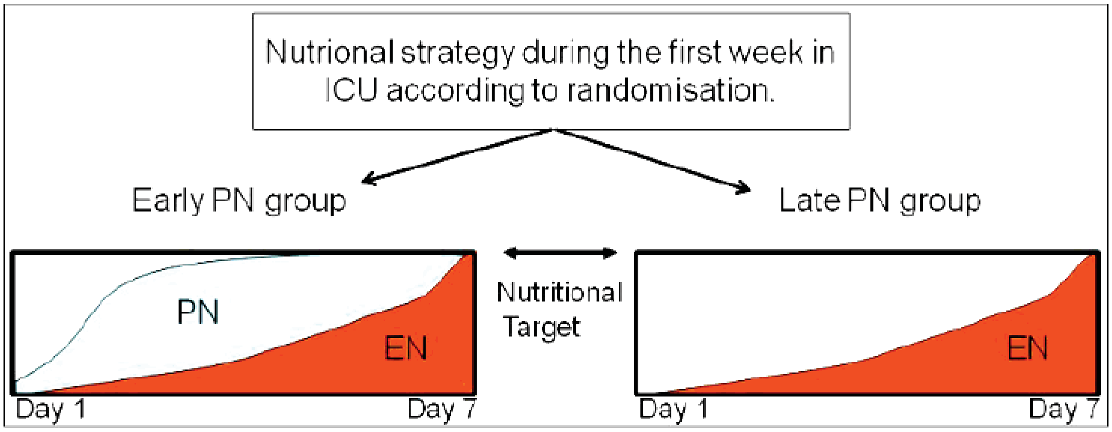

"Late PN", the so-called "intervention-group".

In Leuven, Rotterdam and Edmonton patients randomised to the "late PN" group will receive a mixture of Glucose 5% and NaCl 0.9% at, respectively, 60% and 40% of the total flow rate that is required to obtain standard optimal hydration taking into account the volume of EN that is being delivered. If enteral feeding of at least 80% of the calculated calories is not possible after 7 days in ICU, PN, as specified below, is initiated on day 8.

The infusions will be supplemented with micronutrients and vitamins as they would have received when provided PN.

When the blood glucose levels fall spontaneously below 50 mg/dl, the standard glucose of 5% in the late PN group will be switched to 10% glucose until blood glucose level is above 80 mg/dl. At this point the infusion of glucose 10% will be stopped and switched again to glucose 5%.

Early PN", this nutritional regime is the standard therapy in the participating centres and therefore accounts as the "control-group".

All centres will use their current pharmaceutical nutritional products for enteral as well as parenteral nutrition. The differences between the products provided by the different pharmaceutical companies are small clinically irrelevant differences in glucose, lipid and protein intakes.

Despite the different pharmaceutical companies, nutritional regimes from all participating centres will be aiming for the same energy and protein targets.

Fluid intake will be provided as follows; patients not requiring fluid restriction receive 100 ml/kg/day for the first 10 kg bodyweight, 50 ml/kg for the next 10 kg, and 20 ml/kg for the bodyweight over 20 kg, patients who require fluid restriction, total fluid intake is 50% on day 1 and 2, and 75% on day 3.

Nutritional target calculations

Energy intake

• < 10 kg bodyweight : 100 kcal/kg/day

• 10-20 kg bodyweight : 1000 kcal/day + (50 kcal/kg/day for weight over 10 kg)

• > 20 kg bodyweight : 1500 kcal/day + (20 kcal/kg/day for weight over 20 kg)

Protein target

• 0-10 kg bodyweight : 1.5-3 g/kg/day

• 10-20 kg bodyweight : 1-3 g/kg/day

• > 20 kg bodyweight : 1-2 g/kg/day

Day 1:

In the PICUs from all centers, patients randomised to the "early PN" group upon admission to PICU receive a glucose mixture [Leuven; Glucose15%/Vaminolact® (Fresenius), Rotterdam; Glucose 10–20% (Baxter)] to achieve glucose intake approximately double of the intake in the "late PN" group, with additional trace elements (Peditrace®) and minerals (Addamel®, Novum, Fresenius) and vitamins (Cernevit®, Soluvit®, Vitintra®; Baxter) to be administered in all centers.

Day 2-3:

For all patients on intravenous (IV) nutrition, lipids [Leuven; (20g/100ml) SMOFlipid® Fresenius, Sweden, Rotterdam; (20g/100ml) Intralipid® (Baxter), Oliclinomel® (Baxter)] are added from day 2-3 onward, and increased depending on the age and within the fluid limitation potentially required by the patient.

Additionally, protein intake [Leuven; Vaminolact® or Vamin18® (Fresenius, Sweden), Rotterdam; Primene® (Baxter), Oliclinomel® (Baxter)] will be increased to reach target goals.

On day 2-7, pharmacy-prepared PN preparations are prescribed to achieve protein and energy goals, as described above, unless adequate enteral nutritional intake is expected.

Any enterally delivered energy is taken into account twice daily to reduce the energy delivered by PN. When EN covers 80% of optimal calculated caloric needs, PN is stopped.

When the patient starts to take oral nutrition, the PN and/or EN is reduced and eventually stopped. Whenever enteral or oral intake falls below 50% of calculated caloric needs, the PN is restarted.

The volumes of PN and EN to be given according to the treatment group are calculated by the patient data management system (PDMS). These calculations are based on the nutritional intake during the previous day and the clinical evolution of the patient. The amount of protein and glucose administered during the previous day, as well as the target, will be displayed by the PDMS to further guide the prescription of macronutrients.

The additional trace elements (Peditrace®) and minerals (Addamel® Novum®, Fresenius) and vitamins (Cernevit®, Soluvit®, Vitintra®; Baxter) will be administered in all centers daily until until patients receive at least 80% of their caloric intake enterally.

Enteral nutrition and micronutrient administration

In all patients from both study arms, provided haemodynamically stable and without formal contraindication, EN is initiated on the afternoon following the ICU admission with the patients in semirecumbent position. Enteral feeding will start 6 hrs after admission in the PICU and will be done according to protocol (cfr appendix 1). Trace elements (Peditrace®) and minerals (Addamel® Novum®, Fresenius) and vitamins (Cernevit®, Soluvit®, Vitintra®; Baxter) will be administered daily IV to all patients from day 2 at 4:00 pm. IV micronutrient substitution will be stopped when patients receive at least 80% of their caloric needs via the enteral route.

In infants, breast milk, the patient's home milk formula or a protein-energy dense formula (Nutrilon®, Infatrini®; Nutricia Netherlands) is used. Older children receive standard commercially available enteral feeding (Nutrison®, Nutrini-formulas; Nutricia Netherlands) unless contra-indicated.

Enteral feeding is administered through a gastric tube in a continuous way and is gradually increased as dictated by tolerance. Switch to oral intake is made as soon as deemed safe. The increase of EN volume, the use of gastroprokinetics and duodenal feeding tubes are described in standing-orders for EN. Procedures for slow parenteral administration will be identical for all patients.

Blood glucose management

Blood glucose management will be performed as through standard practice in all participating centers. (for protocols see the appendices)

In **Leuven**, patients in both study groups receive continuous insulin infusion to target blood glucose levels of 50-80 mg/dl when aged < 1y and 70-100 mg/dl when aged >= 1 year. Blood glucose and potassium are monitored systematically every 1 – 4 hours on the blood gas analyser (ABLRadiometer ®, Copenhagen, Denmark) using arterial blood samples.(14)

In **Rotterdam**, patients in all age groups receive continuous insulin infusion to target blood glucose levels of 72-145 mg/dl, except for patients with traumatic brain injury (108-145 mg/dl), using a step-wise nurse driven glucose control protocol. Blood glucose and potassium are monitored systematically every 1 – 3 hours on the blood gas analyser (ABL 625; Radiometer®, Copenhagen, Denmark) using arterial blood samples.(15)

In **Edmonton** patients in all age groups receive continuous insulin infusion to target blood glucose levels <180 mg/dl

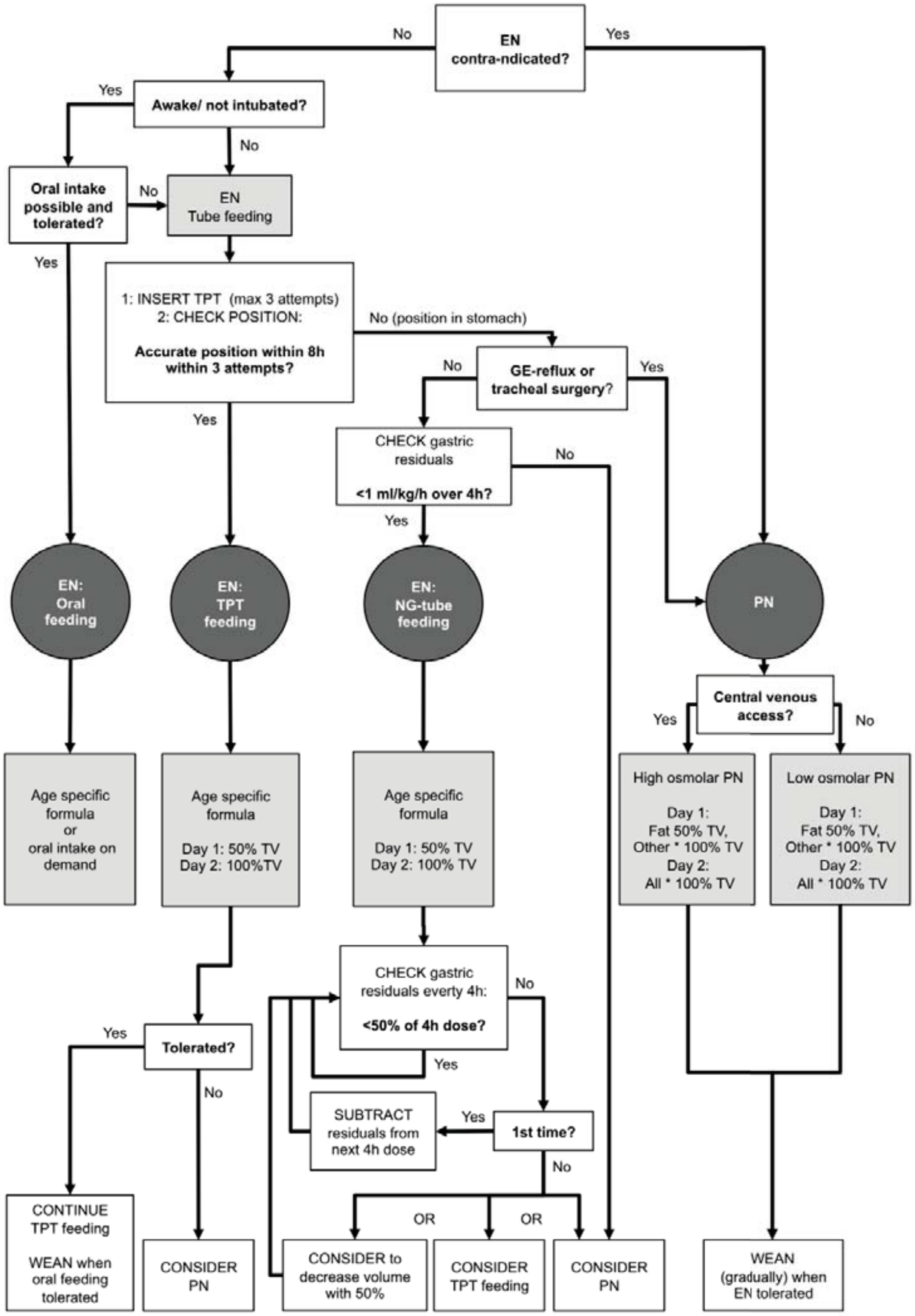

Nutritional protocol showing schematic decision tree

Handling of re-admissions to ICU

Patients who are re-admitted to ICU after a participation in PEPaNIC are not eligible for re-inclusion. Patients who are readmitted to the PICU within 48 hours of discharge and who are still within the 7 days time window of the initial randomization receive the nutrition-schedule they were assigned to during the initial ICU admission. Patients readmitted later will be fed at the discretion of the attending physician.

16.2 Use of co-intervention (if applicable)

Not applicable

16.3 Escape medication (if applicable)

Not applicable

17. INVESTIGATIONAL MEDICINAL PRODUCT

Not applicable.

18. METHODS

18.1 Study parameters/endpoints

18.1.1 Main study parameter/endpoint

The primary efficacy endpoints of for this RCT are included into **Work Package 1** and are the incidence of new infections and the time to discharge alive from ICU. Number of patients with new infections and types of infection will be assessed by numbers and percentages, and the duration of any antibiotics therapy initiated after randomization for those patients requiring antibiotics will be analyzed by non-parametrical tests. As the time of ICU discharge to the regular ward may be affected by the availability of beds on the regular wards, which could induce bias, we *a priori* decided to analyze “time to discharge from ICU” as “time to ready for discharge from ICU”. A patient is considered “ready for discharge” as soon as all clinical conditions for ICU discharge have been fulfilled (no longer in need for vital organ support). Time to discharge alive from ICU will be reported by Kaplan-Meier plots, with ICU non-survivors censored beyond the longest ICU stay of survivors and censoring time of patients still in the ICU at closing of the data file (90 days after last patient inclusion) over both treatment groups. The impact of “late PN” versus “early PN” will be analyzed, with and without correction for age, nutritional status and risk categories and type and severity of illness, by Cox proportional hazard analysis. The distribution of the actual time to discharge from ICU will be reported for ICU-survivors and ICU-non-survivors separately. In view of the time window of the randomized intervention in ICU, also the proportion of patients staying beyond 8 days in ICU will be reported.

18.1.2 Secondary study parameters/endpoints

Work Package 1

All analyses will be performed uncorrected as well as corrected for age, nutritional status and risk categories and type and severity of illness. Time-to-event analysis will be analyzed similarly as the primary endpoint. Proportion of patients requiring support of vital organ functions and distribution of duration of support will be analyzed by non-parametric or parametric testing depending on the normality of the distribution in the subgroup of patients for which support was needed. Proportions will be compared using chi-square testing. Results of repeated measurements will be analyzed using an appropriate model for longitudinal data.

a. Time to final (alive) weaning from mechanical respiratory support: patients still on mechanical respiratory support at closing of the data file (90 days after last patient inclusion) will be censored at that time point. ICU non-survivors will be censored beyond the longest duration of mechanical respiratory support of the survivors.

b. Kidney failure: Proportion of patients in need for renal replacement therapy (RRT) during ICU stay; distribution of duration of RRT (for those patients requiring RRT); proportion of patients with a post-randomization diagnosis of new kidney injury/failure

(defined by modified Risk, Injury, Failure, Loss, and End-stage Kidney (RIFLE) classification criteria as a plasma creatinine doubling or more during ICU stay) in both treatment groups. In addition, the duration of a score RIFLE≥2 will be used as a marker of time to recovery of kidney damage.

c. Need for pharmacological or mechanical haemodynamic support during ICU stay, and duration of such need.

In addition, time to final (alive) weaning from all pharmacological or mechanical haemodynamic support in ICU will be analyzed, with ICU non-survivors censored beyond the longest duration of pharmacological or mechanical haemodynamic support of the survivors and censoring time of patients still on such support at closing of the data file (90 days after last patient inclusion) over both treatment groups.

d. Number of readmissions to the PICU.

e. Liver dysfunction: Proportion of patients during the time window of the intervention and during the whole ICU stay presenting with cholestatic or cytolytic liver dysfunction will be compared.

f. Inflammation: Effect of the intervention on inflammation will be analyzed by comparing the distribution of the highest value reached during ICU stay and changes from baseline to the highest value and by comparing time profiles of daily C-Reactive Protein values.

g. Child health questionnaire scores in both treatment groups will be compared at 3 and 12 months post-discharge.

h. Enteral nutrition (EN) tolerance: proportion of patients with diarrhoea, high gastric residual volumes, vomiting and/or abdominal swelling and relation with buccal epithelial cell expression of chemokines (CXCL-8. CXCL-9 and CXCL-10)

Work Package 2: Health economy analysis

As PN is a major contributor to the healthcare costs of critically children and the clinical outcome is not yet known, an extensive health economic analysis will be included in the study planning. The economic analysis will be performed from a health care perspective and will be estimated for the period during hospitalization OR one year after admission?. The economic evaluation will be performed in accordance with the Dutch guidelines (27).

First, we will calculate and compare the direct medical costs of early versus late PN.

Real medical costs will be calculated by multiplying the volumes of health care use with the corresponding unit prices. *B*ased on the analysis of the patients' detailed invoices, we will allocate healthcare costs into 8 categories (per diem hospitalization costs, honoraria, pharmacy costs, clinical chemistry costs, radiology costs, blood products, graft products, miscellaneous), representing the different reimbursed services and products during ICU and hospital stay. These cost categories have been validated in the health economic analysis of the adult EPaNIC study.

*T*he drug costs will be analyzed using the first level of the World Health Organisation (9) Anatomical Therapeutic Chemical (ATC) classification. We hypothesize that antimicrobials (class J) are the determining driver for differences in pharmacy costs. This is based on the idea that late PN will reduce the incidence of new infections in the ICU.

A model will be constructed to estimate the hypothetical cost difference between early PN and late PN if all acquisition costs related to PN would have been chargeable. This modeling is important to make the results interpretable for readers in different healthcare systems. PN is in variable proportions charged to the patient directly, the hospital or the insurers. Therefore, the data from Rotterdam and Leuven will be converged to one hypothetical system of PN billing.

Costs for inpatient days in hospitals will be estimated as real, basic costs per day using detailed hospital administrative information. For the calculation of other medical costs, we will used charges as published in Dutch guidelines as a proxy of real costs (27).

All analyses will be performed on an intention-to-treat basis. The cost differences between early and late PN will be analyzed using the Mann–Whitney U test. Since cost data per patient (but not per day care) are typically highly skewed, we use nonparametric bootstrap techniques to derive a 95% confidence interval for the differences in distributions of the direct medical costs.

Furthermore, we will explore whether late PN is cost-effective compared to early PN. This will be assessed by calculating the incremental cost-effectiveness ratio (ICER), defined as the difference in costs of late versus early PN, divided by the average change in effectiveness. The primary effect measure is number of patients with a prevented new infection in ICU. Secondary outcome measure is quality of life as measured by validated questionnaires. Overall utility scores for population-based quality of life will be obtained and expressed as QALY's. QALY's will be calculated by multiplying the utility of a health state by the time spent in this health state.

*C*ost-effectiveness of late PN as compared to early PN will be analyzed as the difference in PN costs per patient with a prevented new infection in ICU. In the ideal scenario late PN will result in an improvement of the clinical outcome as well as

a reduction of the healthcare costs. If late PN conveys a clinical improvement at a higher cost, this cost effectiveness in three health care systems will be important to guide the respective governmental health care departments into the decisions of the reimbursement of PN.

For the health economic analysis we will collaborate with the finance & accounting and pharmacy departments of the three participating hospitals. Template database structures will be set-up in advance during the enrolment of the patients in the trial to allow an efficient health economic analysis in the framework of the study.

Work Package 3: Mechanistic analyses in relation to short- and long-term outcome

Impact of early versus late PN on infection and the inflammatory response

The incidence of new, ICU-acquired, infections is a primary efficacy endpoint in the presented study. Based on the results of the adult EPaNIC study (1), we anticipate a lower infection rate with late PN as compared with early PN. We will investigate whether differences in leukocyte function could explain in part such effect. Therefore, blood samples will be taken at pre-set time points during ICU stay for the isolation of monocytes and granulocytes to assess their chemotactic, phagocytosis and oxidative burst capacity (17, 18). Antimicrobial defense depends on, amongst others, efficient clearance of intracellular

pathogens in the autophagic pathway (xenophagy) (10). Indeed, inactivation of autophagy in macrophages and neutrophils has been shown to increase susceptibility to infection. It has previously been demonstrated an autophagy-deficiency phenotype in liver and skeletal muscle of fed prolonged critically ill patients (11). Importantly, a recent study in prolonged critically ill rabbits demonstrated a role for early PN in suppression of the autophagic pathway during critical illness (19). If autophagy would be similarly compromised in leukocytes by early PN, such effect may contribute to a reduced incidence of infections with late PN. We will address this question in a subset of well-matched patients. We will also measure the p62 protein levels of isolated white blood cells obtained upon admission and on day 3 and 7 in ICU, which is known to accumulate when autophagy is deficient/insufficient, as well as the LC3-II/LC3-I ratio as a marker of mature autophagosome formation (20-22). In addition, we will isolate leukocyte DNA from all patients to investigate whether genetic predisposition by single nucleotide polymorphisms (SNPs) in genes of the autophagic core machinery may play a role in susceptibility to infections and whether any such SNP would interact with the nutritional approaches of the study design. The autophagy pathway and/or proteins also appear to play a crucial role in the control of inflammatory signaling and regulation of inflammatory transcriptional responses (10). In this regard, it has been shown that increased levels of p62 activate the pro-inflammatory transcription factor NF-kB. We will study the impact of early versus late PN on pro- and anti-inflammatory cytokines (interleukin-1beta (IL-1beta), IL-2, IL-4, IL-5, IL-6, IL-8, IL-10, tumor-necrosis-factor-alpha, interferon-gamma).

Impact of early versus late PN on the neuroendocrine axes

Critical illness is hallmarked by striking alterations in the hypothalamic-pituitary-peripheral hormone axes (neuroendocrine axes), according to a biphasic pattern (23). The peripheral effector hormone levels are reduced in both phases of critical

illness, although the etiology is different with peripheral target organ resistance in the acute phase and relative hypopituitarism in the prolonged (chronic) phase.

The hypothalamic-pituitary-adrenal axis shows a biphasic, but somewhat different response as compared with the other axes, with cortisol as effector hormone being high in both phases. The responses of the neuroendocrine axes during prolonged critical illness overall have been linked to the development of a characteristic hypercatabolic state, resulting in feeding-resistant muscle wasting, and the severity of the disturbances has been associated with the high risk of morbidity and mortality of the patients. Not only excessive activation of the hypothalamic-pituitary-adrenal axis, but also adrenal insufficiency contributes to morbidity in critically ill children (24).

It was previously hypothesized that strict blood glucose control with intensive insulin therapy would mildly reactivate the somatotropic and thyrotropic axes and anabolism in critically ill children in view of a larger functional capacity of the hypothalamus and pituitary, and the higher amount of nutrition they receive as compared with adult critically ill patients. Unexpectedly and despite improved ICU outcome, however, this therapy further suppressed the somatotropic axis and increased the urea/creatinine ratio as a marker of catabolism (25). Also the low-T3 syndrome could not be reversed by this therapy (unpublished data).

Unpublished data obtained in a rabbit model of prolonged critical illness suggest that relative fasting during critical illness reduces (peripheral) activation of the somatotropic and thyrotropic axes with lower levels of insulin-like growth factor-I (IGF-I) and triiodothyronine (T3) levels, but also attenuates the rise in the stress hormone cortisol as compared with moderate dose intravenous feeding during critical illness. These findings urge a thorough analysis of the impact of early versus late PN on these neuroendocrine axes in the critically ill children included in our study. We hypothesize that late PN results in suppression of the neuroendocrine axes, in concert with the intensive insulin therapy. Unlike conventional belief, we also hypothesize that such suppressed neuroendocrine axes during prolonged critical illness could be associated with a beneficial acute and long-term clinical outcome.

We will evaluate the activation of the somatotropic axis in the acute and chronic phase of critical illness by measuring the levels of growth hormone (GH), acid-labile subunit (ALS), IGF-I and its binding proteins (IGFBP-1 and IGFBP-3), the activation of the thyrotropic axis by analysis of thyroxin (T4), T3, reverse T3 (rT3) and thyroid stimulating hormone (TSH), and the adrenal axis by adrenocorticotropic hormone (ACTH) and cortisol. The metabolism of cortisol will be studied further by the relative levels of its metabolites excreted in urine (26) and by collecting hair samples for the long term cortisol levels. The impact of early versus late PN on the neuroendocrine axes will be studied in relation to markers of catabolism, muscle volume and strength, as well as clinical outcome. The clinical outcome will be assessed during follow-up consultations that are held in the departments that have referred their patients to the ICU in Leuven and Rotterdam.

The epigenetics of early versus late PN

It has been known since a long time that dietary exposures can have health consequences years or decades later. E.g. the later health of babies who were in the womb during the Dutch famine in the winter of 1944 was greatly affected by the caloric restriction of their mothers. The children of pregnant women exposed to famine were shown to be more susceptible to chronic adult diseases such as diabetes, obesity and cardiovascular disease. Apparently, the short period of caloric

restriction can be "remembered" by the body further on in life. There is a growing body of evidence that epigenetic mechanisms mediate this memory. Epigenetic mechanisms alter the gene expression without changing the primary DNA sequence. Rather the epigenetic mechanisms work through DNA methylation, histone

modifications and non-coding microRNAs. During the clinical trial, biological material (spun-down cells from whole blood samples) will be collected from the study patients to compare these epigenetic changes between the early and late PN treatment groups. The mechanistic findings will be correlated with the acute and the long-term outcome of the critically ill study patients.

18.1.3 Other study parameters

Safety endpoints

Safety endpoints comprise vital status, hypoglycemia, SAEs and complications related to the mode of nutrition. Survival up to 90 days after randomization in both treatment groups will be compared by Kaplan Meier survival plots. The impact of "late PN" versus "early PN" will be analyzed, with and without correction for risk factors, by Cox proportional hazard analysis. In addition, we will record in-ICU and in-hospital mortality, and will analyze differences with Chi-square testing. As the randomized study intervention only takes place during a time window up to the 8th day in ICU, we also plan to analyze early lethality within this time window in the intention-to-treat population as part of the safety analysis.

As patients not receiving early PN may be considered at increased risk for hypoglycemia, we will report for both groups the number of patients experiencing hypoglycemia <40 mg/dl during the time window of the randomized intervention. Hypoglycemia resistant to parenteral glucose administration is considered as a SAE and the incidence during the time window of the randomized intervention will be reported for both groups. In addition, overall blood glucose control during the time window of the intervention will be compared using daily morning blood glucose as well as daily maximal and minimal blood glucose.

Safety issues also comprise the occurrence of feeding-mode-related complications during the time window of the intervention. Therefore, occurrence of these complications (digestive intolerance, complicated insertion of feeding tubes, pneumothorax, haemothorax and subclavian or carotid artery puncture, occlusion or displacement of central venous catheters or gastric feeding tubes) will be reported for both treatment groups.

18.2 Randomisation, blinding and treatment allocation

Randomization

The study will use a prospective, randomized, controlled, parallel-group design. On admission patients will be randomly assigned to receive EN combined with early PN or only EN. At ICU admission, consecutive patients will be randomly assigned to one of these two treatment groups using a centralized computer randomisation. Randomisation will be done in a 1:1 allocation ratio in permuted blocks of 10 (The block size remains unknown to bed-side physicians and nurses, responsible for patient recruitment and therapy assignment) and stratified according to primary diagnostic category on admission:

I Medical-ICU admissions (infectious or non-infectious): (a) respiratory (b) cardiac (c) renal (d) haematological/oncological (e) gastro-intestinal/hepatic (f) neurological (g) other.

II Surgical-ICU admissions (elective or urgent) according to referral discipline (a) cardiac surgery (b) solid organ transplants (c) pulmonary/oesophageal surgery (d) abdominal surgery (e) neurosurgery (f) trauma/orthopaedic surgery (g) burns (more than 20% BSA is burned and/or patient requires ventilation)

Blinding of treatment allocation

Treating physicians and patients cannot be blinded. All outcome assessors, which are investigators not directly involved in the patients care (such as statisticians, laboratory personnel, infectious disease specialists, pathologists and physiotherapists involved in the strength measurement) as well as physicians and nurses in the conventional wards are blinded to treatment allocation.

18.3 Study procedures

Data collection following recruitment

Baseline characteristics

At baseline, data on demographic and clinical characteristics of the patients are obtained. Disease specific risk scores (such as the Risk-Adjustment in Congenital Heart Surgery or RACHS score) are calculated, co-morbidities and known use of important medications prior to admission are noted: these comprise, among others, the presence of congenital disease or syndrome, gestational age at birth, gender, ethnicity, paediatric risk scores, presence / history of cancer, diabetes mellitus, kidney failure, liver failure, chronic heart failure and sepsis upon admission. In addition, we record the need for and the number of days of mechanical ventilatory support, of mechanical and pharmacological haemodynamic support, of renal replacement therapies, days on antibiotics and days requiring a central line.

Outcome characteristics

All medications received by the patients during ICU stay are registered. Every day the amount of kilocalories, lipids, proteins, carbohydrates delivered by either PN or EN are calculated from the PDMS in an automated manner and entered into the case record form (CRF). Interruptions of EN delivery and predefined digestive intolerance are registered daily. Mechanical complications such as displacement or obstruction of the enteral feeding tube or the central venous catheter, and clinical complications such as pneumothorax, hemothorax and subclavian or carotid artery puncture are recorded daily. All medications received by the patients during ICU stay are registered. Every day the amount of kilocalories, lipids, proteins, carbohydrates delivered by either PN or EN are calculated from the PDMS in an automated manner and entered into the case record form (CRF). Of the gastric residual volume discarded, half of the volume will be considered to be EN and half gastric secretions. The duration (in min) and cause of interruption of delivery of EN will be recorded. Digestive

intolerance will be registered as vomiting, tracheal aspiration of enteral feeding (defined 8), diarrhea, and gastric residue above 5ml/kg (see appendix for definitions). Tube displacement or obstruction will be labeled as mechanical complications. Occlusion and dislodging of central venous catheters will be recorded as mechanical complications. Pneumothorax, hemothorax and arterial puncture will be recorded as clinical complications. Number of ICU days with a central line in situ will be noted. The characteristics will be monitored until discharge from ICU, day 90 of hospital stay or death.

Blood samples

The maximum blood volume will be maximum 5 ml/kg for the entire study period. For a detailed description of the blood samples and tests see figure below. Blood samples are taken upon ICU admission and daily until discharge from ICU, or death. The blood samples will be taken from lines placed for clinical purposes or in combination with pricks requested for clinical purposes. A subset of the samples are immediately stored on ice for future endocrine measurements. Processed serum and plasma as well as the spun-down blood cells will be stored and frozen for mechanistic analyses.

All whole blood glucose levels are measured on arterial blood using a blood gas analyser on each ICU and are registered for later calculation of glucose metrics. Analyses on blood and urine for the primary clinical analyses include routine chemistry, haematology, and markers of inflammation. Further epigenetic, metabolic, endocrine and inflammatory measurements on stored samples in the context of mechanistic analyses will be stored until final analysis. Urine samples will be stored and frozen until final analysis.

Primary endpoint characteristics

All new infections of the lungs, the blood stream, the urinary tract and wounds are recorded by an infectious disease specialist. Bacteraemia is further classified by responsible pathogen and as catheter-related blood stream infection versus other bacteraemia.

Additional data collection

For mechanistic and exploratory studies, muscle strength testing (> 6 years of age) at several moment during admission will be performed.

Urine (nitrogen / bone metabolism / cortisol metabolites / biomarkers for renal failure; NGAL) and stool samples (biomarkers gut function; citrulline / calprotectin) will be collected on daily basis.

We will also perform standard anthropometric tests, upper-arm and lower-leg circumference, in addition to standard weight and height for age. These analyses will be repeated at the follow-up at 3 and 12 months post-discharge, on condition of obtaining adequate additional funding.

Buccal swabs will be collected at day of admission (day 1), day 2, 4 and 6, and possibly on day of discharge and will be immersed in RNA-later and stored at -80C. Buccal epithelial cell expression of chemokines (CXCL-8. CXCL-9 and CXCL-10) will be measured in by Taqman analysis.

Around 100 strands of hair from the posterior vertex of the scalp will be cut off as soon as possible after admission and this will be repeated after 3 weeks- 2 months if possible.

<u>Questionnaires and long term follow-up</u>

Furthermore, as far as practically feasible, every patient is approached just prior to hospital discharge, to complete a validated, semi-structured child health questionnaire (Functional Independence Measure (WEEFIM), Health Utilities Index (HUI) and Child Health Questionnaire (CHQ)).

We also will thoroughly assess long-term clinical outcome. This not only encompasses survival rate 3 years after inclusion in the study. It will also involve a detailed functional and neurocognitive examination, by means of questionnaires such as mentioned above. These analyses will be repeated at the follow-up at 3 and 12 months post-discharge, on condition of obtaining adequate additional funding. This will be organised in the framework of current follow-up consultations of these critically ill children.

Figure Samples.

Blood samples are taken upon ICU admission and daily at 06:00h until discharge from ICU, death or end of study. Upon admission three blood samples will be taken; namely two yellow "STOL"-tubes each containing a maximum of 3.5 milliliter blood and 1 purple "EDTA"-tube of 1.8 milliliter blood.

During the following days until the patient has left the PICU we will daily take a yellow "STOL"-tube (containing max 3,5 milliliter blood) and a purple "EDTA"-tube (containing 1,8 milliliter blood) for further analyses. On day 3,5 and 7 a 'PAX-tube' (containing 2 milliliter blood) will be collected. In children > 10 kg 1,8 milliliter extra blood (EDTA and heparine) will be collected on 4 selected days.

A subset of the samples will be immediately stored on ice for future endocrine measurements. Processed serum and plasma as well as the spun-down blood cells will be frozen and stored.

Analyses on the morning sample will include routine clinical chemistry, hematology (Hgb, WBC, TC), and markers of inflammation (CRP), liver function ALT, AST, ALP, GGT, bilirubin total/direct. A number of metabolic, hormonal and inflammatory study analyses will be performed on selected days.

The latter comprise, among others, coagulation & fibrinolysis tests, cytokines and markers of oxidative stress.

Also on selected days stool (single sample,) urine (5 tubes of 5 mL out of 24 hours' urine collection), 100 strands of hair and buccal swabs will be stored for further evaluation.

All whole blood glucose levels are measured on arterial blood using a blood gas analyser on each ICU and are registered for later calculation of glucose metrics.

18.4 Withdrawal of individual subjects

Subjects can leave the study at any time for any reason if they wish to do so without any consequences. The investigator can decide to withdraw a subject from the study for urgent medical reasons.

18.4.1 Specific criteria for withdrawal (if applicable)

Not applicable

18.5 Replacement of individual subjects after withdrawal

Subjects that withdraw after randomization has been conducted will not be replaced, subjects that have not been allocated and withdraw after signing the consent form but before the start of randomization will be replaced.

18.6 Follow-up of subjects withdrawn from treatment

A register is kept of all patients evaluated for inclusion and of patients who withdraw from the study. The latter are clinically followed up without their data being analyzed in the study, according to intention-to-treat analysis.

18.7 Premature termination of the study

As the previous study in adults has shown that lethality was not different among the study groups and that morbidity was prevented by withholding feeding, and since the participating centers are currently providing early PN, repeated interim analyses for efficacy are not required. However, one formal “safety” interim analysis will be planned after patient number 720 has left the ICU, during which the independent DSMB will have access to un-blinded results on ICU mortality, hospital mortality and serious adverse events. The DSMB will then judge on whether or not to prematurely stop the clinical trial for safety reasons, or to perform more safety interim analyses. The DSMB will advise on eventual continuation of the study to completion, under monitoring of the serious adverse event. Because the primary efficacy

endpoint will not be analyzed, no correction of the significance level at the final analysis will be necessary. In (the unlikely) case the study would be terminated prematurely, the mechanistic studies will be even more important, as the clinical community will ask for due explanations for harm caused during the study. Therefore, in any case they will be started shortly after all clinical data have been collected.

19. SAFETY REPORTING

The risk in participating to the study and being randomized to the "late PN' group are negligible, and specifically compass an increased risk of developing hypoglycemia and/or underfeeding. However, safety measures will be taken to further decrease these risks. Our study may provide support for current practice, may give “neutral” results for which the cost-effectiveness study will provide necessary information for guiding therapy, or may challenge the presumed innocence of PN. Thus, there is no risk associated with the intrinsic value of these results.

The burden is expected to be minimal as it will only entail additional blood draws, which will be taken from clinical lines or in addition to pricks for clinical purpose. The long-term follow up will be held as part of already organised follow-up outdoor clinics and which are developed to help children and their parents to recover physically, emotionally and socially after ICU admissions. For mechanistic and exploratory studies, muscle strength testing and an ultrasound evaluation of the skeletal muscle and adipose tissue compartments will be performed. Furthermore, as far as practically feasible, every patient is approached just prior to hospital discharge, to complete a validated, semi-structured child health questionnaire (Functional Independence Measure (WEEFIM), Health Utilities Index (HUI) and Child Health Questionnaire (CHQ)).

In order to monitor the quality of the enteral and parenteral nutrition management during the study we will register all known complications possibly related to them. These complications should not be considered as adverse events since the study intervention is to withhold parenteral feeding during one week. These known complications of parenteral feeding will not be reported to the sponsor until the end of the trial.

***Complications possibly related to enteral feeding**

Digestive intolerance: either vomiting, tracheal aspiration of enteral feeding (defined in 7), diarrhea, or gastric residue above 5ml/kg, abdominal distention (see appendix for definitions).

Complicated insertion of feeding tubes: nasal bleeding

Mechanical complications Feeding tube displacement or obstruction

***Complications possibly related to parenteral feeding**

Mechanical complications Occlusion and dislodging of central venous catheters

Clinical complications: pneumothorax, hemothorax and arterial puncture, central line replacement due to suspicion of catheter-related blood stream infections

Safety endpoints

Safety endpoints comprise vital status, hypoglycaemia, SAEs and complications related to the mode of nutrition. Survival up to 90 days after randomisation in both treatment groups will be compared by Kaplan Meier survival plots. The impact of “late PN” versus “early PN” will be analysed, with and without correction for risk factors, by Cox proportional hazard analysis. In addition, we will record in-ICU and in-hospital mortality, and will analyse differences with Chi-square testing. As the

randomised study intervention only takes place during a time window up to the 8th day in ICU, we also plan to analyse early lethality within this time window in the intention-to-treat population as part of the safety analysis.

As patients not receiving early PN may be considered at increased risk for hypoglycaemia, we will report for both groups the number of patients experiencing hypoglycaemia <40 mg/dl during the time window of the randomised intervention. Hypoglycaemia resistant to parenteral glucose administration is considered as a SAE and the incidence during the time window of the randomised intervention will be reported for both groups. In addition, overall blood glucose control during the time window of the intervention will be compared using daily morning blood glucose as well as daily maximal and minimal blood glucose.

Safety issues also comprise the occurrence of feeding-mode-related complications during the time window of the intervention. Therefore, occurrence of these complications (digestive intolerance, complicated insertion of feeding tubes, pneumothorax, haemothorax and subclavian or carotid artery puncture, occlusion or displacement of central venous catheters or gastric feeding tubes) will be reported for both treatment groups

Interim analysis

As the previous study in adults has shown that lethality was not different among the study groups and that morbidity was prevented by withholding feeding, and since the participating centres are currently providing early PN, repeated interim analyses for efficacy are not required. However, one formal "safety" interim analysis will be planned after patient number 720 has left the ICU, during which the independent DSMB will have access to un-blinded results on ICU mortality, hospital mortality and serious adverse events. The DSMB will then judge on whether or not to prematurely stop the clinical trial for safety reasons, or to perform more safety interim analyses. The DSMB will advise on eventual continuation of the study to completion, under monitoring of the serious adverse event. Because the primary efficacy endpoint will not be analysed, no correction of the significance level at the final analysis will be necessary.

19.1 Section 10 WMO event

In accordance to section 10, subsection 1, of the WMO, the investigator will inform the subjects and the reviewing accredited METC if anything occurs, on the basis of which it appears that the disadvantages of participation may be significantly greater than was foreseen in the research proposal. The study will be suspended pending further review by the accredited METC, except insofar as suspension would jeopardise the subjects' health. The investigator will take care that all subjects are kept informed.

19.2 Adverse and serious adverse events

Adverse events are defined as any undesirable experience occurring to a subject during the study, whether or not considered related to the experimental treatment. All adverse events reported spontaneously by the subject or observed by the investigator or his staff will be recorded.

A serious adverse event is any untoward medical occurrence or effect that at any dose:

- results in death;
- is life threatening (at the time of the event);
- requires hospitalisation or prolongation of existing inpatients' hospitalisation;
- results in persistent or significant disability or incapacity;
- is a new event of the trial likely to affect the safety of the subjects, such as an unexpected outcome of an adverse reaction, lack of efficacy of an IMP used for the treatment of a life threatening disease, major safety finding from a newly completed animal study, etc.

All SAEs will be reported through the web portal *ToetsingOnline* to the accredited METC that approved the protocol, within 15 days after the sponsor has first knowledge of the serious adverse reactions. SAEs that result in death or are life threatening should be reported expedited. The expedited reporting will occur not later than 7 days after the responsible investigator has first knowledge of the adverse reaction. This is for a preliminary report with another 8 days for completion of the report.

Safety endpoints specifically for PEPaNIC

The clinical research team guarantees a daily follow-up of patient screening and inclusion, availability of requested clinical data in the clinical patient files and protocol compliance. Each non-compliance to the protocol and other questions or problems are reported to the study monitor and discussed with the principal investigators. Serious Adverse Events (SAE) are also reported to the study coordinating investigator/project leader (K.U.Leuven). The study monitor regularly provides the sponsor with reports on inclusions and SAE. Regular meetings are organized with principal investigators and clinical research team to discuss the daily progression of the research project.

As patients not receiving early PN may be considered at increased risk for hypoglycemia, we will report for both groups the number of patients experiencing hypoglycemia <40 mg/dl during the time window of the randomized intervention. Hypoglycemia resistant to parenteral glucose administration, or with clinical symptoms, is considered as a SAE and the incidence during the time window of the randomized intervention will be reported for both groups. In addition, overall blood glucose control during the time window of the intervention will be compared using daily morning blood glucose as well as daily maximal and minimal blood glucose.

Further safety endpoints comprise vital status, hypoglycemia, SAEs and complications related to the mode of nutrition. Survival up to 90 days after randomization in both treatment groups will be compared by Kaplan Meier survival plots. The impact of "late PN" versus "early PN" will be analyzed, with and without correction for risk factors, by Cox proportional hazard analysis. In addition, we will record in-ICU and in-hospital mortality, and will analyze differences with Chi-square testing. As the randomized study intervention only takes place during a time window up to the 8th day in ICU, we also plan to analyze early lethality within this time window in the intention-to-treat population as part of the safety analysis.

Safety issues also comprise the occurrence of feeding-mode-related complications during the time window of the intervention. Therefore, occurrence of these complications (digestive intolerance, complicated insertion of feeding tubes, pneumothorax, haemothorax and subclavian or carotid artery puncture, occlusion or displacement of central venous catheters or gastric feeding tubes) will be reported for both treatment groups.

Above mentioned SAE's and safety endpoints will be handled and reported within the standard timeframe as described above.

19.2.1 Suspected unexpected serious adverse reactions (SUSAR)

Not applicable

19.2.2 Annual safety report

Not applicable

19.3 Follow-up of adverse events

All adverse events will be followed until they have abated, or until a stable situation has been reached. Depending on the event, follow up may require additional tests or medical procedures as indicated, and/or referral to the general physician or a medical specialist.

19.4 Data Safety Monitoring Board (DSMB)

The coordinating investigator/project leader (K.U.Leuven) provides direct access to the CRF, the source data and the study master file for monitoring, Independent Ethics committee review and regulatory inspection. The coordinating investigator/project leader (K.U.Leuven) established an independent data safety monitoring board (DSMB) (Prof. dr. J Vranckx, Prof. dr. em. R Bouillon, Prof. dr. em. P Lauwers, Prof.dr. M Bruynooghe (statisticus)), which holds no conflicts of interest with the sponsor or coordinating investigator/project leader (K.U.Leuven). The coordinating investigator/project leader (K.U.Leuven) appoints one monitor. The monitor verifies that the trial is performed in accordance to the protocol as described in the European Medicine Agency's "Note for guidance on good clinical practice CPMP/ICH/135/95" as well as the Declaration of Helsinki. Monitoring will be performed and will be reported following the sponsor's standing operating procedures. No fault insurance is covered by Fortis Corporate Insurance NV (Leuven) and Akkermans Van Elten Assurantiën BV (Rotterdam).

One formal "safety" interim analysis will be planned after patient number 720 has left the ICU, during which the independent DSMB will have access to un-blinded results on ICU mortality, hospital mortality and serious adverse events. The DSMB will

then judge on whether or not to prematurely stop the clinical trial for safety reasons, or to perform more safety interim analyses. The DSMB will advise on eventual continuation of the study to completion, under monitoring of the serious adverse event. Because the primary efficacy

endpoint will not be analyzed, no correction of the significance level at the final analysis will be necessary.

The advice(s) of the DSMB will be notified upon receipt by the sponsor to the METC that approved the protocol. With this notification a statement will be included indicating whether the advice will be followed.

20. STATISTICAL ANALYSIS

20.1 Descriptive statistics

The current statistical analysis plan comprises the primary and secondary clinical endpoints of this RCT (work package 1). These include the acute clinical effects of the intervention during ICU stay and hospitalization, including survival up to 90 days after randomization.

The sample size (N=1440, 720 per arm) is calculated in order to detect, with at least 80% power (one- tailed; the two-tailed power is 70%) and 95% certainty, a reduction in PICU infections from 20% to 15% and, with at least 90% power (2-tailed) and 95% certainty, a reduction in mean duration of stay in PICU of 1 day. With this sample size, and for safety reasons, also any substantial impact on mortality (increase or decrease with an absolute +/- 2%, although unexpected taken the adult data), can be excluded with a power of around 62-75% depending on the true mortality in the total population. We plan to calculate the true power of the study for detection of any eventual smaller differences in these outcomes.

General rules of the statistical analysis

All analyses will be done on intention to treat basis. The analyses will be performed on the whole set of patients and by subgroups of patients based on the primary diagnostic categories used as prognostic factors for stratification and on septic/non-septic groups of patients.

A consort diagram will be reported. The data file will be finalised 90 days after inclusion of the last patient. To assess compliance with the study protocol, the amounts of PN and EN actually given in the two study groups during the intervention window of 7 days will be reported as absolute numbers and percentages of target calories. Discrete variables will be summarised by frequencies and percentages and analysed by (exact) Chi- square test or logistic regression analysis. Continuous variables will be summarised by use of either mean or standard deviations (SD) or median and interquartile range as appropriate and compared using Student's t-test or Mann-Whitney-U test, as appropriate. Time to event analysis will be performed by Cox proportional hazard analysis. All outcomes will be analysed in an uncorrected manner as well as (jointly) corrected for risk factors (type and severity of illness, age, on admission nutritional status and risk scores). *A priori* defined subgroup analyses will be performed for patients admitted to ICU after cardiac surgery as compared with all other patients; for patients with and without sepsis upon admission; for patients with contra-indications for EN on admission or not. For all endpoints, differences will be considered statistically significant whenever the p-value is lower than 0.05 without correction for multiple testing.

Safety endpoints

Safety endpoints comprise vital status, hypoglycaemia, serious adverse events and complications related to the mode of nutrition. Survival up to 90 days after randomization in both treatment groups will be compared by Kaplan Meier survival plots. The impact of "late PN" versus "early PN" will be analyzed, with and without correction for risk factors, by Cox

proportional hazard analysis. In addition, we will record vital status at ICU and hospital discharge, and will analyze differences with Chi-square testing. As the randomized study intervention only takes place during a time window up to the 8th day in ICU, we also plan to analyze early lethality within this time window in the intention to treat population as part of the safety analysis.

20.2 Univariate analysis

Discrete variables will be summarized by frequencies and percentages and analyzed by (exact) Chi-square test or logistic regression analysis. Continuous variables will be summarized by use of either mean or standard deviations (SD) or median and interquartile range as appropriate and compared using Student's t-test or (exact) Mann-Whitney-U test, as appropriate. Time to event analysis will be performed by Cox proportional hazard analysis. Time to discharge alive from ICU will be reported by Kaplan-Meier plots, with ICU non-survivors censored beyond the longest ICU stay of survivors and censoring time of patients still in the ICU at closing of the data file (90 days after last patient inclusion) over both treatment groups. The impact of "late PN" versus "early PN" will be analyzed, with and without correction for age, nutritional status and risk categories and type and severity of illness, by Cox proportional hazard analysis. The distribution of the actual time to discharge from ICU will be reported for ICU-survivors and ICU-non-survivors separately. In view of the time window of the randomized intervention in ICU, also the proportion of patients staying beyond 8 days in ICU will be reported.

20.3 Multivariate analysis

All outcomes will be analyzed in an uncorrected manner as well as (jointly) corrected for risk factors (type and severity of illness, age, on admission nutritional status and risk scores). Another *a priori* defined subgroup analysis will be performed for patients admitted to ICU after cardiac surgery as compared with all other patients. Also an *a priori* subgroup analysis is planned for patients with and without sepsis upon admission.

All analyses will be performed uncorrected as well as corrected for age, nutritional status and risk categories and type and severity of illness. Time-to-event analysis will be analyzed similarly as the primary endpoint. Proportion of patients requiring support of vital organ functions and distribution of duration of support will be analyzed by non-parametric or parametric testing depending on the normality of the distribution in the subgroup of patients for which support was needed. Proportions will be compared using chi-square testing. Results of repeated measurements will be analyzed using an appropriate model for longitudinal data.

a. Time to final (alive) weaning from mechanical respiratory support: patients still on mechanical respiratory support at closing of the data file (90 days after last patient inclusion) will be censored at that time point. ICU non-survivors will be censored beyond the longest duration of mechanical respiratory support of the survivors.

b. Kidney failure: Proportion of patients in need for renal replacement therapy (RRT) during ICU stay; distribution of duration of RRT (for those patients requiring RRT); proportion of patients with a post-randomization diagnosis of new kidney injury/failure (defined by modified Risk, Injury, Failure, Loss, and End-stage Kidney (RIFLE) classification criteria as a plasma creatinine doubling or more during ICU stay) in both treatment groups. In addition, the duration of a score RIFLE≥2 will be used as a marker of time to recovery of kidney damage.

c. Need for pharmacological or mechanical hemodynamic support during ICU stay, and duration of such need.

In addition, time to final (alive) weaning from all pharmacological or mechanical hemodynamic support in ICU will be analyzed, with ICU non-survivors censored beyond the longest duration of pharmacological or mechanical hemodynamic support of the survivors and censoring time of patients still on such support at closing of the data file (90 days after last patient inclusion) over both treatment groups.

d. Number of readmissions to the PICU.

e. Liver dysfunction: Proportion of patients during the time window of the intervention and during the whole ICU stay presenting with cholestatic or cytolytic liver dysfunction will be compared.

f. Inflammation: Effect of the intervention on inflammation will be analyzed by comparing the distribution of the highest value reached during ICU stay and changes from baseline to the highest value and by comparing time profiles of daily C-Reactive Protein values.

g. Child health questionnaire scores (WEEFIM, HUI, CHQ) at hospital discharge in both treatment groups will be compared.

20.4 Interim analysis

As the previous study in adults has shown that lethality was not different among the study groups and that morbidity was prevented by withholding feeding, and since the participating centers are currently providing early PN, repeated interim analyses for efficacy are not required. However, one formal "safety" interim analysis will be planned after patient number 720 has left the ICU, during which the independent DSMB will have access to un-blinded results on ICU mortality, hospital mortality and serious adverse events. The DSMB will then judge on whether or not to prematurely stop the clinical trial for safety reasons, or to perform more safety interim analyses. The DSMB will advise on eventual continuation of the study to completion, under monitoring of the serious adverse event. Because the primary efficacy

endpoint will not be analyzed, no correction of the significance level at the final analysis will be necessary.

The safety analyses will be done on the whole set of patients and by subgroups of patients based on the primary diagnostic categories used as prognostic factors for stratification and on septic/non-septic groups of patients.

21. ETHICAL CONSIDERATIONS

21.1 Regulation statement

The study will be conducted in accordance to the protocol as described in the European Medicine Agency's "Note for guidance on good clinical practice CPMP/ICH/135/95" as well as the Declaration of Helsinki (*59th WMA General Assembly, Seoul, Korea, October 2008*) and in accordance with the Medical Research Involving Human Subjects Act (WMO).

21.2 Recruitment and consent

The study protocol and consent forms will be sent for approval by the Institutional Review Board of the Katholieke Universiteit Leuven and the Erasmus MC (Lokale uitvoerbaarheid) and by the competent Belgian and Dutch authorities (CCMO). Written informed consent is obtained from the parents or the legal guardian by the investigator's team or one of the supervising doctors, who will inform the parents of the patients (and the patients themselves if the age is > 12 years) and ask for their consent. The parents / legal guardians and (if applicable) the patients will receive a patient information letter and an informed consent form.

Written informed consent will be obtained from the patient or the closest family member or legal guardian. For planned PICU admissions after elective procedures, informed consent will be asked beforehand, if possible.

For emergency PICU admissions, treatment allocation will be done after assessment of the patient for eligibility by the attending physician within the time frame of two hours. If eligible, the patient will be randomized into the study and the allocated glucose infusion (with micronutrients) will be started. Informed consent will be asked within the time frame of 24 hours (deferred informed consent) as a nutritional regimen has to be initiated already on admission. When consent is given, the allocated nutritional regimen will proceed (Early (with additional lipids and protein targeting nutritional goals < day3) vs Late (no PN)). When NO consent is given, the nutritional regimen and glucose infusion will be placed under responsibility of the supervising clinical team of doctors.

The parents or legal guardians can withdraw the patient from the study at any time, without penalty or impact on treatment.

21.3 Objection by minors or incapacitated subjects (if applicable)

The children can withdraw from the study at any time, without penalty or impact on treatment. (art 4 lid 2 WMO) Neither will the child be forced to undergo the additional tests, such as muscle strength testing, the ultrasound evaluation of the skeletal muscle and adipose tissue compartments, or the questionnaires and follow-up evaluations. In this matter, also non-verbal resistance will be taken into account by the investigator's team. The specific test will then not be performed, without taking the subject out of the study as a whole.

21.4 Benefits and risks assessment, group relatedness

During the informed consent process, it will be made extremely clear that participation in this study will provide no direct benefits to the patient and that refusal to participate will have zero impact on the care received by any of the nursing medical staff. The risks will be kept to a minimum. This study requires this specific study group of critically ill children of different age groups. Although the study has been performed in adults, these results should not be translated directly to children as both the metabolic as well as nutritional kinetics in children of different age groups vary significantly form adults.

21.5 Compensation for injury

The sponsor/investigator has a liability insurance which is in accordance with article 7, subsection 6 of the WMO.

No fault insurance is covered by Fortis Corporate Insurance NV (Leuven) and Akkermans Van Elten Assurantiën BV (Rotterdam).

The sponsor has an insurance which is in accordance with the legal requirements in the Netherlands (Article 7 WMO and the Measure regarding Compulsory Insurance for Clinical Research in Humans of 23th June 2003). This insurance provides cover for damage to research subjects through injury or death caused by the study.

4. € 450.000,-- (i.e. four hundred and fifty thousand Euro) for death or injury for each subject who participates in the Research;
5. € 3.500.000,-- (i.e. three million five hundred thousand Euro) for death or injury for all subjects who participate in the Research;
6. € 5.000.000,-- (i.e. five million Euro) for the total damage incurred by the organisation for all damage disclosed by scientific research for the Sponsor as 'verrichter' in the meaning of said Act in each year of insurance coverage.

The insurance applies to the damage that becomes apparent during the study or within 4 years after the end of the study.

21.6 Incentives

Not applicable

22. ADMINISTRATIVE ASPECTS AND PUBLICATION

22.1 Handling and storage of data and documents

Data are collected electronically in a pseudonymized CRF, unambiguously linked to the source file. The subject identification codes are safeguarded by the principle investigators. Data are manually transferred (and checked for accuracy) into the CRF by the clinical research assistance team on a daily basis from the ICU PDMS and the Leuven University Hospitals Clinical Working Station (KWS). Extensive range and consistency checks are performed by the study monitor. Vital status at 90 days will be recorded for all patients, by the National Death Registries when this information is not available in the hospital information system or the regional network of pediatricians.

The handling of personal data will comply with the Dutch Personal Data Protection Act (in Dutch: De Wet Bescherming Persoonsgegevens, WBP).

22.2 Amendments

Amendments are changes made to the research after a favourable opinion by the accredited METC has been given. All amendments will be notified to the METC that gave a favourable opinion.

All substantial amendments will be notified to the METC and to the competent authority.

Non-substantial amendments will not be notified to the accredited METC and the competent authority, but will be recorded and filed by the sponsor.

22.3 Annual progress report

The sponsor/investigator will submit a summary of the progress of the trial to the accredited METC once a year. Information will be provided on the date of inclusion of the first subject, numbers of subjects included and numbers of subjects that have completed the trial, serious adverse events/ serious adverse reactions, other problems, and amendments.

22.4 End of study report

The investigator will notify the accredited METC of the end of the study within a period of 8 weeks. The end of the study is defined the time-point 90 days after the inclusion of the last patient.

In case the study is ended prematurely, the investigator will notify the accredited METC, including the reasons for the premature termination.

Within one year after the end of the study, the investigator/sponsor will submit a final study report with the results of the study, including any publications/abstracts of the study, to the accredited METC.

22.5 Public disclosure and publication policy

This is an investigator initiated study. This study will be registered as a clinical trial in a public trial registry.

The investigators aim to publish all results obtained from the study unreservedly.

23. REFERENCES

1. Casaer MP, Mesotten D, Hermans G, Wouters PJ, Schetz M, Meyfroidt G, et al. Early versus late parenteral nutrition in critically ill adults. N Engl J Med 2011;365(6):506-17.

2. Singer P, Berger MM, Van den Berghe G, Biolo G, Calder P, Forbes A, et al. ESPEN Guidelines on Parenteral Nutrition: intensive care. Clin Nutr 2009;28(4):387-400.

3. Skillman HE, Wischmeyer PE. Nutrition therapy in critically ill infants and children. JPEN J Parenter Enteral Nutr 2008;32(5):520-34.

4. Zamberlan P, Delgado AF, Leone C, Feferbaum R, Okay TS. Nutrition therapy in a pediatric intensive care unit: indications, monitoring, and complications. JPEN J Parenter Enteral Nutr 2011;35(4):523-9.

5. Hulst JM, Zwart H, Hop WC, Joosten KF. Dutch national survey to test the STRONGkids nutritional risk screening tool in hospitalized children. Clin Nutr 2010;29(1):106-11.

6. Verbruggen SC, Coss-Bu J, Wu M, Schierbeek H, Joosten KF, Dhar A, et al. Current recommended parenteral protein intakes do not support protein synthesis in critically ill septic, insulin-resistant adolescents with tight glucose control. Crit Care Med 2011;39(11):2518-25.

7. Verbruggen SC, Schierbeek H, Coss-Bu J, Joosten KF, Castillo L, van Goudoever JB. Albumin synthesis rates in post-surgical infants and septic adolescents; influence of amino acids, energy, and insulin. Clin Nutr 2011;30(4):469-77.

8. Verbruggen S, Sy J, Arrivillaga A, Joosten K, van Goudoever J, Castillo L. Parenteral Amino Acid Intakes in Critically Ill Children: A Matter of Convenience. JPEN J Parenter Enteral Nutr 2010;34(3):329-40.

9. Derde S, Vanhorebeek I, Guiza F, Derese I, Gunst J, Fahrenkrog B, et al. Early Parenteral Nutrition Evokes a Phenotype of Autophagy Deficiency in Liver and Skeletal Muscle of Critically Ill Rabbits. Endocrinology 2012.

10. Levine B, Mizushima N, Virgin HW. Autophagy in immunity and inflammation. Nature 2011;469(7330):323-35.

11. Vanhorebeek I, Gunst J, Derde S, Derese I, Boussemaere M, Guiza F, et al. Insufficient activation of autophagy allows cellular damage to accumulate in critically ill patients. J Clin Endocrinol Metab 2011;96(4):E633-45.

12. Vlasselaers D, Milants I, Desmet L, Wouters PJ, Vanhorebeek I, van den Heuvel I, et al. Intensive insulin therapy for patients in paediatric intensive care: a prospective, randomised controlled study. Lancet 2009;373(9663):547-56.

13. WHO ‘Prevention of hospital-acquired infections; a practical guide, 2nd edition’ *www.who.int/csr/.../whocdscsreph200212.pdf*

14. Vlasselaers D, Mesotten D, Langouche L, Vanhorebeek I, van den Heuvel I, Milants I, et al. Tight glycemic control protects the myocardium and reduces inflammation in neonatal heart surgery. Ann Thorac Surg 2010;90(1):22-9.

15. Verhoeven JJ, Brand JB, van de Polder MM, Joosten KF. Management of hyperglycemia in the pediatric intensive care unit; implementation of a glucose control protocol. Pediatr Crit Care Med 2009;10(6):648-52.

16. WHO. Management of severe malnutrition: a manual for physicians and other senior health workers. Geneva: World Health Organization 1999.

17. Ellger B, Debaveye Y, Vanhorebeek I, Langouche L, Giulietti A, Van Etten E, et al. Survival benefits of intensive insulin therapy in critical illness: impact of maintaining normoglycemia versus glycemia-independent actions of insulin. Diabetes 2006;55(4):1096-105.

18. Henckaerts L, Nielsen KR, Steffensen R, Van Steen K, Mathieu C, Giulietti A, et al. Polymorphisms in innate immunity genes predispose to bacteremia and death in the medical intensive care unit. Crit Care Med 2009;37(1):192-201, e1-3.

19. Derde S, Vanhorebeek I, Ververs EJ, Vanhees I, Darras VM, Van Herck E, et al. Increasing intravenous glucose load in the presence of normoglycemia: effect on outcome and metabolism in critically ill rabbits. Crit Care Med 2012;38(2):602-11.

20. Bjorkoy G, Lamark T, Pankiv S, Overvatn A, Brech A, Johansen T. Monitoring autophagic degradation of p62/SQSTM1. Methods Enzymol 2009;452:181-97.

21. Komatsu M, Waguri S, Ueno T, Iwata J, Murata S, Tanida I, et al. Impairment of starvation-induced and constitutive autophagy in Atg7-deficient mice. J Cell Biol 2005;169(3):425-34.

22. Masiero E, Agatea L, Mammucari C, Blaauw B, Loro E, Komatsu M, et al. Autophagy is required to maintain muscle mass. Cell Metab 2009;10(6):507-15.

23. Van den Berghe GH. Acute and prolonged critical illness are two distinct neuroendocrine paradigms. Verh K Acad Geneeskd Belg 1998;60(6):487-518; discussion 518-20.

24. Langer M, Modi BP, Agus M. Adrenal insufficiency in the critically ill neonate and child. Curr Opin Pediatr 2006;18(4):448-53.

25. Gielen M, Mesotten D, Brugts M, Coopmans W, Van Herck E, Vanhorebeek I, et al. Effect of intensive insulin therapy on the somatotropic axis of critically ill children. J Clin Endocrinol Metab 2011;96(8):2558-66.

26. Cuzzola A, Petri A, Mazzini F, Salvadori P. Application of hyphenated mass spectrometry techniques for the analysis of urinary free glucocorticoids. Rapid Commun Mass Spectrom 2009;23(18):2975-82.

27. Hakkaart-van Roijen L et al. Handleiding voor kostenonderzoek, methoden en standaard kostprijzen voor economische evaluaties in de gezondheidszorg. CVZ. 2010

Informatie over het onderzoek:

"Vroege versus Late Infuusvoeding bij zieke kinderen"

Beste jongere,

Korte samenvatting van deze informatiebrief

Deze brief is gemaakt om je te vragen mee te doen aan een onderzoek. Eerste willen je kort wat informatie geven. Van dit onderzoek word jij zelf niet meteen beter, maar in de toekomst hopen we wel zieke kinderen zoals jij te helpen met betere voeding. Het belangrijkste van het onderzoek houdt in dat we jou, als je gedurende een week zelf niet voldoende eet, wel of niet een voedingsinfuus geven. Hoewel je hierdoor misschien meer gewicht verliest omdat je ziek bent, denken wij door de uitslagen van andere onderzoeken die gedaan zijn, dat het geven van een voedingsinfuus ook veel nadelen zou kunnen hebben. Hierdoor is het mogelijk dat je juist door het uitstellen van dit voedingsinfuus sneller beter wordt, maar dit weten we niet zeker, want dit is alleen nog maar bij zieke volwassen patiënten gezien. Als je gewoon kunt eten, of in ieder geval eten via een sonde in je maag kunt krijgen dan krijg je dit wel. Alleen extra voeden via een infuus wordt dan misschien niet gegeven. Of je dit wel of niet krijgt, wordt geloot door een computer.

Wij zullen voor het onderzoek extra bloed, urine, poep, haar en wangslijmvlies verzamelen, en daarnaast wat extra metingen van je lengte en gewicht doen. Als je weer beter bent en van onze afdeling weg mag, vragen we je om thuis nog een vragenlijst in te vullen.

Het is belangrijk om te weten dat jij zelf beslist of je mee wilt doen met het onderzoek. Dit doe je met het opschrijven van je naam en als je die hebt een handtekening. Ook mag je op elk moment voor maar ook tijdens het onderzoek zeggen dat je met het onderzoek wilt stoppen.

Hier onder staat alles nog wat uitgebreider opgeschreven als je dit wilt lezen. Daarnaast willen jouw dokters of de mensen van het onderzoek het je ook uit leggen door met je te praten en vragen te beantwoorden.

Inleiding

Je bent in het Sophia Kinderziekenhuis omdat je behandeling nodig hebt op onze intensive care afdeling. Wij begrijpen dat de opname de intensive care erg rot is voor jou.

Toch willen we je vriendelijk vragen om mee te doen aan een onderzoek (zie titel). Jij en je ouders beslisen zelf of jij mee wilt doen. Voordat je de beslissing neemt, is het belangrijk om meer te weten over het onderzoek. De informatie die je ontvangt bestaat uit twee delen, de algemene brochure 'Medisch wetenschappelijk onderzoek' en onze informatie brief. Lees allebei rustig door. Je kunt het bespreken met je ouders.

Heb je na het lezen van de informatie nog vragen? Dan kunnen jij en je ouders altijd terecht bij de onderzoekers. Op bladzijde 5 (paragraaf 18) staan de contactgegevens.

Als je mee wilt doen aan het onderzoek vragen wij of je handtekening of je naam op het toestemmingsformulier wilt schrijven. Je krijgt van dit formulier dan ook een kopie.

Het onderzoek wordt gedaan op de afdeling Intensive Care van het Sophia Kinderziekenhuis -Erasmus MC. Het onderzoek wordt ook uitgevoerd in het Universitair Ziekenhuis te Leuven, België en het Kinderziekenhuis in Edmonton, Canada.

1. Wat is het doel van het onderzoek?

Je bent en voelt je nu erg ziek en daarvoor krijg je verschillende vormen van behandeling. Een onderdeel van die behandeling die je krijgt is voeding met suikers, vetten en eiwitten. Suikers, vetten en eiwitten heb je nodig om beter te worden en om te groeien. Maar omdat je ziek bent kun je niet voldoende zelf eten. Om dit te verbeteren kunnen we in een ziekenhuis de voeding ook geven via een sonde of eventueel zelfs via een infuus als dat nodig is.

Het geven van voeding via een infuus omdat het via je darmen niet gaat heeft ook nadelen. Zoals het risico's op infecties, ontstekingen, maar het is ook minder gezond voor je lever. Daarnaast kunnen we nog steeds niet alle voedingsstoffen in de goede hoeveelheid via het infuus geven. Er is twijfel ontstaan (door studies die al klaar zijn) of je bij iemand die ziek is wel meteen met voeding via het infuus moet starten, of dat er even afgewacht moet worden tot de voeding via de darmen weer beter gaat. Hoewel je hierdoor misschien meer gewicht zult verliezen in de eerste dagen van ziek zijn, zouden de nadelen van een voedingsinfuus hier mogelijk tegen op wegen. Dit willen wij onderzoeken in een studie die maximaal 7 dagen duurt. De gehele studieperiode waarin we naast jou nog andere kinderen op onze afdeling zullen volgen zal in totaal ongeveer 3 jaar duren. Deze studie is al eerder gedaan bij volwassenen, waaruit bleek dat de groep volwassenen waarbij er werd gewacht met het voedingsinfuus het beter deed (minder infecties, lagen korter op de afdeling en in het ziekenhuis).

Een ander doel van het onderzoek is om te kijken naar het verdragen van voeding via de darmen, waarom lukt dat wel of niet. Dit doen we door naar verschillende factoren te kijken die van invloed zijn op het verdragen van voeding door de darm, namelijk darmhormonen, darmschade en ontsteking in de darm.

2. Welke behandeling wordt onderzocht?

Wij kijken of het afwachten (van maximaal 7 dagen) van voeding via het infuus beter is dan het zo snel mogelijk starten van dit infuus.

3. Hoe wordt het onderzoek uitgevoerd?

In 4 jaar tijd zullen 1440 kinderen meedoen aan dit onderzoek. Er worden 2 groepen van 720 kinderen onderzocht. Het onderzoek duurt voor jou maximaal 7 dagen nadat jij en je ouders toestemming hebben gegeven. Jouw gegevens zullen tijdens de hele opname op de afdeling verzameld worden.

Als je besluit om mee te doen aan deze voedingsstudie dan wordt er geloot of je voeding via het infuus krijgt, of dat we afwachten tot je darmen het weer zelf kunnen.

Welke behandeling je ook loot, jouw gewone behandeling gaat gewoon door en ook zullen we telkens kijken of je zelf kunt eten, of dat het met de sonde gaat. Het verschil in de behandeling is dat als je zelf binnen 7 dagen niet kunt eten, je dan wel of niet een voedingsinfuus krijgt. En het gaat dan alleen om eiwitten en vetten. De suikers, vitamines en zouten krijg je wel via het infuus, ongeacht wat je loot.

Als je binnen 7 dagen beter bent en van onze afdeling af kunt dan stopt de studie. Als je na 7 dagen nog steeds te ziek bent om zelf te eten of voeding via de darmen te krijgen, dan zal er toch worden gestart met voeding via het infuus. Dit doen we omdat we denken dat nog langer wachten een risico op ondervoeding zou geven.

4. Wat merk jij van het onderzoek?

- Wij zullen de infusen en de bloedafnames doen met de infusen die je nu al hebt, dus je hoeft hiervoor niet extra geprikt te worden. De bloedafnames zijn 1 x per dag, in principe als je van je eigen dokter toch al een bloedafnames moest krijgen.
- We zullen op een aantal dagen een kleine hoeveelheid wangslijmvlies bij je afnemen om te kijken naar cellen uit het afweersysteem. Het afnemen van het wangslijmvlies doen we door met een borsteltje langs de binnenkant van je wangen te wrijven.
- We zullen een plukje haar (ongeveer 100 haren) van je achterhoofd knippen om te kijken naar het niveau van de stresshormonen. Dit stukje haar zal zo dicht mogelijk bij je hoofdhuid afgeknipt worden. Het plukje haar dat weggeknipt wordt zal zo geknipt worden dat de rest van je haar hier weer overheen valt, waardoor het niet te zien is.
- We zullen je vragen om met een test mee te doen waarmee we je spierkracht meten. Dit doet geen pijn.
- Jij en je ouders zullen gevraagd worden om een vragenlijst in te vullen. Deze vragen gaan over hoe je je voelt en wat je na het ziek zijn inmiddels weer kunt. Deze vragenlijst sturen wij 3 en 12 maanden na jouw opname op de Intensive Care afdeling naar jullie op.
- Ook zullen we je, nadat je van onze afdeling ontslagen bent, vragen om op de polikliniek langs te komen om te meten en te wegen.

5. Wat is meer of anders dan jouw normale behandeling(en)?

Als je loot voor het “late” voedingsinfusen, dan krijg je geen extra infuus met eiwitten en vetten tot maximaal 7 dagen.

6. Wat zijn de andere mogelijke behandelingen?

Er zijn geen andere mogelijke behandelingen, wanneer je niet aan het onderzoek deelneemt, zul je de standaard behandeling krijgen. Dat is momenteel nog het zo snel mogelijk starten met voeding via het infuus.

7. Welke bijwerkingen kunt u verwachten?

Wij verwachten dat dit onderzoek geen bijwerkingen zal geven, maar er kan een risico bestaan op groter gewichtsverlies, of ondervoeding. Wij zullen gedurende het onderzoek door middel van metingen goed in de gaten houden of hoe goed je groeit. Hoewel deze studie is opgezet met het idee dat een mogelijk gewichtsverlies in de eerste dagen van ziek zijn niet nadelig is en opweegt tegen de nadelen van overvoeding weten we dit op dit moment nog niet zeker.

8. Wat zijn mogelijke voor- en nadelen van deelname aan dit onderzoek?

- De mogelijke bijwerkingen hebben we bij punt 7. genoemd.
- We zullen extra bloed afnemen, maar daarvoor gebruiken we je eigen infusen en wordt je niet extra geprikt.
- We zullen wangslijmvlies afnemen door met een borsteltje langs de binnenkant van je wangen te wrijven. Je kunt hierdoor kortdurend een raar gevoel hebben. Dit doet geen pijn.
- Er wordt een plukje haar (ongeveer 100 haren) van je achterhoofd weggeknipt. Het plukje haar dat weggeknipt wordt zal zo geknipt worden dat de rest van het haar hier weer overheen valt, waardoor het niet te zien is. Er zijn geen risico’s of bijwerkingen bekend van deze handeling.
- We zullen je vragen om vragenlijsten in te vullen over jouw situatie en jouw herstel. Tevens vragen we je om in een later vervolg nog eens op de polikliniek te komen voor deze vragen en te wegen en meten.
- Je hebt zelf geen direct voordeel als je aan het onderzoek meedoet.
  Als je meedoet help je de kennis over hoe we zieke kinderen beter en veiliger kunnen voeden.

9. Wat gebeurt er als je je verzet bij het onderzoek?

Het behandelteam van de afdeling Intensive Care begeleiden jou en je ouders tijdens het onderzoek zo goed mogelijk. Dit team bestaat onder meer uit gespecialiseerde artsen, onderzoekers en verpleegkundigen. Als de belasting voor jouw groter is dan verwacht, zullen we stoppen met het onderzoek. Dit doen we volgens landelijke afspraken zoals die door de Nederlandse Vereniging voor Kindergeneeskunde(NVK) zijn vastgelegd ter bescherming van minderjarige onderzoeksdeelnemers. Voor meer informatie hierover verwijzen wij jou en je ouders naar de volgende website: www.ccmo.nl (wet- en regelgeving/gedragscode verzet: minderjarigen).

10. Wat gebeurt er wanneer je besluit niet mee te doen aan dit onderzoek?

Jij beslist zelf met je ouders of je wilt meedoen. Het heeft geen enkel gevolg voor jouw behandeling op de afdeling. Het is alleen maar bedoeld voor de vooruitgang van de geneeskunde. Als je niet wilt meedoen met het onderzoek hoef je niets te tekenen, zonder uitleg of het geven van een reden.

Voor meer informatie verwijzen we u naar pagina 8 t/m 12 van de Algemene brochure.

11. Wat gebeurt er als het onderzoek is afgelopen?

- Er zitten geen risico's aan het stoppen van het onderzoek, ook niet als het nog niet is afgelopen.
- Het onderzoek stopt op het moment dat de studie volgens het protocol is afgelopen, of op het moment dat jij of je ouders het onderzoek willen stoppen. Ook kan het zijn dat de onderzoeker of het behandelteam het onderzoek stopt.

12. Ben je verzekerd wanneer je aan het onderzoek meedoet?

Voor iedereen die meedoet aan dit onderzoek is een verzekering afgesloten, deze verzekering is wettelijk verplicht. Meer informatie vindt u in de Algemene brochure op pagina 14.

In bijlage 2 vind je de verzekerde bedragen, de uitzonderingen en de adresgegevens van de verzekeraar.

13. Word je geïnformeerd als er tussentijds voor jou relevante informatie over de studie bekend wordt?

Het onderzoek zal zo nauwkeurig mogelijk volgens plan verlopen. Maar de situatie kan veranderen. Bijvoorbeeld door de reactie van jouw lichaam, of door nieuwe informatie. Als dat zo is, bespreken we dat meteen met jou. Je beslist dan zelf of je met het onderzoek wilt stoppen of doorgaan. Als jouw veiligheid in gevaar is, stoppen wij meteen met het onderzoek.

14. Wat gebeurt er met jouw gegevens?

De gegevens blijven geheim, krijgen een code en jouw naam wordt weggelaten.

De gegevens worden bewaard gedurende 15 jaar. Dit mag alleen wanneer jij daar toestemming voor geeft.

Lichaamsmateriaal, dit is bloed en urine, wordt gedurende het onderzoek met een code bewaard en na afloop van het onderzoek vernietigd.

De mensen die jouw gegevens kunnen zien zijn het onderzoeksteam, de toetsingscommissie, de veiligheidscommissie en de inspectie van de gezondheidszorg.

Voor meer informatie verwijzen we je naar pagina 12, 14 en 15 van de Algemene brochure.

15. Wordt jouw huisarts geïnformeerd bij deelname?

Wij kunnen de huisarts (schriftelijk) laten weten dat je meedoet aan het onderzoek. Dit is voor je eigen veiligheid. Je geeft hiervoor toestemming op het toestemmingsformulier.

16. Zijn er extra kosten/ is er een vergoeding wanneer je besluit uw kind aan dit onderzoek mee te laten doen?

Meedoen aan het onderzoek kost jou of je ouders niets. Je krijgt ook geen vergoeding

(bijvoorbeeld geld of kado) als je meedoet aan het onderzoek.

17. Welke medisch-ethische toetsingscommissie heeft dit onderzoek goedgekeurd?

De Centrale Commissie Mensgebonden Onderzoek (CCMO) heeft dit onderzoek goed gekeurd. Meer informatie over de goedkeuring en controle van het onderzoek vind je in de Algemene brochure op pagina 13.

18. Wil je verder nog iets weten?

Voor het bedenken of je mee wilt doen krijg je bedenktijd. Je kunt altijd iets vragen aan je eigen dokter of aan het het team van onderzoekers.

De onderzoekers

Als je tijdens of na het onderzoek nog vragen of opmerkingen heeft, kun je dat doen aan een van de onderzoekers. Je kunt hen tijdens kantooruren bereiken via het centrale telefoonnummer **010-7040704**, je kunt daarna vragen naar de desbetreffende onderzoeker.

- Dorian Kerklaan, arts-onderzoeker Intensive Care, tel. 06-83214901
- Dr. S. Verbruggen, Kinderarts Intensive Care, Tel. 06-24792312
- Dr. Koen Joosten, Kinderarts Intensive Care, zoemer 36918

Onafhankelijke arts
Indien je twijfelt over deelname of meer informatie wenst over het onderzoek kun je een onafhankelijke arts raadplegen. Deze arts is niet zelf bij het onderzoek betrokken, maar wel deskundig op het gebied van onderzoek. De onafhankelijk arts voor dit onderzoek is:

Prof. Dr. I Reiss, kinderarts in het Sophia Kinderziekenhuis / Erasmus MC

Telefoonnummer: 010-7040704, zoemer 36026

Klachten

Als je niet tevreden bent over de gang van zaken tijdens het onderzoek, dan kunnen jij en je ouders terecht bij de onderzoekers, de behandelend kinderarts of de onafhankelijke klachtencommissie van het ziekenhuis: tel. 010-7033198.

19. Bijlagen

1. Algemene brochure medisch-wetenschappelijk onderzoek met mensen.
2. Verzekering

Bijlage 2. Verzekering

Voor de deelnemers aan dit onderzoek is een verzekering afgesloten. Deze verzekering dekt schade door letsel of dood die het gevolg is van deelname aan het onderzoek, en die zich gedurende de deelname aan het onderzoek openbaart, of binnen vier jaar na beëindiging van de deelname aan het onderzoek. De schade wordt geacht zich te hebben geopenbaard wanneer deze bij de verzekeraar is gemeld.

In geval van schade kunt u zich direct wenden tot de verzekeraar.

De verzekeraar van het onderzoek is:

Naam: Meeus Assurantiën BV

Adres: Postbus 3234, 4800 DE Breda

Telefoonnummer: 076-5313480

Contactpersoon: Mw. Mr. F.A.C. Bergervoet (gezondheidsjurist, afdeling Vrije Beroepen)

De verzekering biedt een maximum dekking van € 450.000 per proefpersoon en een maximumbedrag van € 3.500.000 voor het gehele onderzoek. Indien de opdrachtgever van dit onderzoek meerdere onderzoeken heeft lopen, geldt een maximumbedrag van

€ 5.000.000 euro per verzekeringsjaar voor álle onderzoeken.

De dekking van specifieke schades en kosten is verder tot bepaalde bedragen beperkt. Dit is opgenomen in het Besluit verplichte verzekering bij medisch-wetenschappelijk onderzoek met mensen. Informatie hierover kunt u vinden op de website van de Centrale Commissie Mensgebonden Onderzoek: www.ccmo.nl.

Voor deze verzekering gelden een aantal uitsluitingen. De verzekering dekt niet:

- schade waarvan op grond van de aard van het onderzoek zeker of nagenoeg zeker was dat deze zich zou voordoen;
- schade aan de gezondheid die ook zou zijn ontstaan indien u niet aan het onderzoek had deelgenomen;
- schade die het gevolg is van het niet of niet volledig nakomen van aanwijzingen of instructies;
- schade aan nakomelingen, als gevolg van een nadelige inwerking van het onderzoek op u of uw nakomeling;
- bij onderzoek naar bestaande behandelmethoden: schade die het gevolg is van één van deze behandelmethoden;
- bij onderzoek naar de behandeling van specifieke gezondheidsproblemen: schade die het gevolg is van het niet verbeteren of van het verslechteren van deze gezondheidsproblemen.

Toestemmingsformulier behorende bij het onderzoek:

“Vroege versus Late Infuusvoeding bij zieke kinderen”

- Ik ben gevraagd om mee te doen aan dit medisch wetenschappelijke onderzoek:

Mijn Naam ______________________________ Geboortedatum __ / __ / __

dag maand jaar

- Ik heb de informatiebrief gelezen. Ik begrijp de informatie. Ik kon aanvullende vragen stellen. De vragen zijn naar tevredenheid beantwoord. Ik heb voldoende tijd gehad om te beslissen of ik mee wil doen.

- Ik weet dat meedoen helemaal vrijwillig is. Ik weet dat ik op ieder moment kan beslissen om te stoppen. Daarvoor hoef ik geen reden te geven.

- Ik geef toestemming om mijn dokter te vertellen dat ik meedoe aan dit onderzoek.

- Ik weet dat de mensen van het onderzoeksteam, de toestingscommissie, veiligheidscommissie en de inspectie van de gezondheidszorg mijn gegevens kunnen bekijken.

- Ik geef toestemming om de gegevens te gebruiken, voor de doelen die in de informatiebrief staan, en om deze voor 15 jaar te bewaren.

- Ik wil meedoen aan dit onderzoek

- Ik geef wel/geen toestemming om mij na afloop van het onderzoek te benaderen voor vervolgonderzoek

- Ik geef wel/geen toestemming om mijn hoofdhaar af te knippen ten behoeve van wetenschappelijk onderzoek.

__________________________________ ____________

Naam Handtekening Datum __ / __ / __

Dag maand jaar

__________________________________ ____________

Naam ouder/ voogd 1 Handtekening Datum __ / __ / __

Dag maand jaar

___________________________________ ____________ Datum __ / __ / __

Naam ouder/ voogd 2 Handtekening Dag maand jaar

Ik verklaar hierbij dat ik bovengenoemde persoon volledig heb geïnformeerd over het genoemde onderzoek.

Als er tijdens het onderzoek informatie bekend wordt die de toestemming van het kind, de ouder of voogd zou kunnen beïnvloeden, dan breng ik hem/haar daarvan tijdig op de hoogte.

___________________________________ ____________

Naam onderzoeker Handtekening Datum __ / __ / __
dag maand jaar

Functie:____________________________

Aanvullende informatie is gegeven door.

___________________________________ ____________

Naam onderzoeker Handtekening Datum __ / __ / __
dag maand jaar

Functie:____________________________

Informatie over het onderzoek:

“Vroege versus Late Infuusvoeding bij zieke kinderen”

Inleiding
Geachte ouders/ verzorgers,

U bent in het Sophia Kinderziekenhuis omdat uw kind behandeling nodig heeft op onze intensive care afdeling. Wij begrijpen dat de opname van uw kind op de intensive care een ingrijpende gebeurtenis voor u is.

Toch willen we u vriendelijk vragen om uw kind mee te laten doen aan een medisch-wetenschappelijk onderzoek (zie titel). U beslist zelf of u uw kind wilt laten meedoen. Voordat u de beslissing neemt, is het belangrijk om meer te weten over het onderzoek. De informatie die u ontvangt bestaat uit twee delen, de algemene brochure 'Medisch wetenschappelijk onderzoek' en onze informatie brief. Lees beiden rustig door. U kunt het bespreken met uw partner, vrienden of familie.

Hebt u na het lezen van de informatie nog vragen? Dan kunt u terecht bij de onderzoekers. Op bladzijde 5, paragraaf 18 vindt u de contactgegevens.

Als u uw kind mee wilt laten doen aan het onderzoek vragen wij of u het toestemmingsformulier wilt ondertekenen. U krijgt dan ook een kopie van dit formulier.

Het onderzoek wordt gedaan op de afdeling Intensive Care van het Sophia Kinderziekenhuis -Erasmus MC. Het onderzoek wordt ook uitgevoerd in het Universitair Ziekenhuis te Leuven, België en het Kinderziekenhuis in Edmonton, Canada.

1. Wat is het doel van het onderzoek?

Uw kind is erg ziek en krijgt daarvoor verschillende vormen van behandeling. Een onderdeel van die behandeling die uw kind krijgt is voeding met suikers, vetten en eiwitten. Suikers, vetten en eiwitten heb je nodig om beter te worden en om te groeien. Maar omdat uw kind ziek is kun hij/zij niet voldoende zelf eten. Om dit te verbeteren kunnen we in een ziekenhuis de voeding ook geven via een sonde of eventueel zelfs via een infuus als dat nodig is.

Het geven van voeding via een infuus omdat het via de darmen niet gaat heeft ook nadelen. Zoals het risico's op infecties, ontstekingen, maar het is ook minder gezond voor de lever. Daarnaast kunnen we nog steeds niet alle voedingsstoffen in de goede hoeveelheid via het infuus geven. Er is dus twijfel ontstaan door reeds afgeronde studies of er bij iemand die ziek is wel meteen met voeding via het infuus moet starten, of dat er even afgewacht moet worden tot de voeding via de darmen weer wel beter gaat. Hoewel uw kind hierdoor misschien meer gewicht zal verliezen in de eerste dagen van ziek zijn, zouden de nadelen van een voedingsinfuus hier mogelijk tegen op wegen. Dit willen wij onderzoeken in een studie die maximaal 7 dagen duurt. De gehele studieperiode waarin we naast uw kind nog andere kinderen op onze afdeling zullen volgen zal in totaal ongeveer 3 jaar duren. Deze studie is reeds gedaan bij volwassenen, waaruit bleek dat de groep volwassenen waarbij er werd gewacht met het voedingsinfuus het beter deed (minder infecties, lagen korter op de afdeling en in het ziekenhuis).

Een ander doel van het onderzoek is om te kijken naar het verdragen van voeding via de darmen, waarom dat bij het ene kind wel gaat en bij het andere niet. Dit doen we door naar verschillende factoren te kijken die van invloed zijn op het verdragen van voeding door de darm, namelijk darmhormonen, darmschade en ontsteking in de darm.

2. Welke behandeling wordt onderzocht?

Wij kijken of het afwachten (van maximaal 7 dagen) van voeding via het infuus beter is dan het zo snel mogelijk starten van dit infuus.

3. Hoe wordt het onderzoek uitgevoerd?

In 4 jaar tijd zullen 1440 kinderen meedoen aan dit onderzoek. Er worden 2 groepen van 720 kinderen onderzocht. Het onderzoek duurt voor uw kind maximaal 7 dagen nadat u toestemming heeft gegeven. De gegevens van uw kind zullen tijdens de hele opname op de Intensive Care verzameld worden.

Als u besluit om uw kind mee te laten doen aan deze voedingsstudie dan wordt er geloot of er zo snel mogelijk voeding via het infuus wordt gegeven, of dat we afwachten tot de darmen van uw kind het weer zelf kunnen.

Welke behandeling uw kind ook loot, de gewone behandeling gaat gewoon door en ook zullen we telkens kijken of uw kind zelf voeding kan eten, of dat het met de sonde gaat. Het verschil in de behandeling is dat als uw kind zelf binnen 7 dagen niet kan eten, wij dan wel of niet een voedingsinfuus zullen start. En het gaat dan alleen om eiwitten en vetten. De suikers, vitamines en zouten krijgt uw kind wel via het infuus, ongeacht wat uw kind loot.

Als uw kind binnen 7 dagen beter is en van onze afdeling af kan dan stopt de studie. Als uw kind na 7 dagen nog steeds te ziek is om zelf te eten of voeding via de darmen te krijgen, dan zal er toch worden gestart met voeding via het infuus. Dit doen we omdat we denken dat nog langer wachten een risico op ondervoeding zou geven.

4. Wat merkt uw kind van het onderzoek?

- Wij zullen de infusen en de bloedafnames doen met de infusen die uw kind al heeft, dus hij/zij hoeft hiervoor niet extra geprikt te worden. De bloedafnames zijn 1 x per dag, in principe als uw kind van uw eigen dokter toch al een bloedafnames moest krijgen.
- We zullen op een aantal dagen een kleine hoeveelheid wangslijmvlies afnemen bij uw kind om te kijken naar cellen uit het afweersysteem. Dit doen we door met een borsteltje langs de binnenkant van de wangen te wrijven. Dit doet geen pijn.
- We zullen een plukje haar (ongeveer 100 haren) van het achterhoofd afnemen om te kijken naar het niveau van de stresshormonen. Dit stukje haar zal zo dicht mogelijk bij de hoofdhuid afgeknipt worden. Het plukje haar dat weggeknipt wordt zal, waar mogelijk, op een zodanige manier verwijderd worden dat de rest van het haar hier weer overheen valt, waardoor het niet te zien is.We zullen vragen om met een test mee te doen waarmee we de spierkracht van uw kind meten. Dit doet geen pijn.
- U zult gevraagd worden om een vragenlijst in te vullen. Deze vragen gaan over hoe uw kind zich voelt, hoe hij/zij herstelt is en wat hij/zij na het ziek zijn inmiddels weer kan. Deze vragenlijst sturen wij 3 en 12 maanden na de opname op de Intensive Care afdeling naar u op.
- Ook zullen we u en uw kind vragen, om na ontslag op de polikliniek te komen om te meten en te wegen.

5. Wat is meer of anders dan de reguliere behandeling(en) die uw kind krijgt?

Als uw kind loot voor het “late” voedingsinfuus, dan krijgt hij/zij geen extra infuus met eiwitten en vetten tot maximaal 7 dagen.

6. Wat zijn de andere mogelijke behandelingen?

Er zijn geen andere mogelijke behandelingen, wanneer uw kind niet aan het onderzoek deelneemt, zal hij/zij dezelfde behandeling krijgen die hij/zij nu krijgt. Dat is momenteel nog het zo snel mogelijk starten met voeding via het infuus.

7. Welke bijwerkingen kunt u verwachten?

Wij verwachten dat het onderzoek geen bijwerkingen zal geven, maar er kan een risico bestaan op groter gewichtsverlies, of ondervoeding. Wij zullen gedurende het onderzoek door middel van metingen goed in de gaten houden of dit ook daadwerkelijk het geval is. Hoewel deze studie is opgezet met de gedachte dat een mogelijk gewichtsverlies in de eerste dagen van ziek zijn niet nadelig is en opweegt tegen de nadelen van overvoeding weten we dit op dit moment nog niet zeker.

8. Wat zijn mogelijke voor- en nadelen van deelname aan dit onderzoek?

- De mogelijke bijwerkingen hebben we bij punt 7. genoemd.
- We zullen extra bloed afnemen, maar daarvoor gebruiken we de eigen infusen en wordt er niet extra geprikt.
- We zullen wangslijmvlies afnemen door met een borsteltje langs de binnenkant van de wangen te wrijven. Dit kan kortdurend een raar gevoel geven, maar doet geen pijn. Er zijn geen risico's of bijwerkingen bekend van deze handeling.
- Er wordt een plukje haar (ongeveer 100 haren) van het achterhoofd van uw kind weggeknipt. . Het plukje haar dat weggeknipt wordt zal, waar mogelijk, op een zodanige manier verwijderd worden dat de rest van het haar hier weer overheen valt, waardoor het niet te zien is. Er zijn geen risico's of bijwerkingen bekend van deze handeling.
- We zullen u vragen om vragenlijsten in te vullen over uw situatie hoe u en uw kind zelf vinden dat uw kind van zijn/haar ziekte herstelt. Tevens vragen we uw kind om in een later vervolg nog eens op de polikliniek te komen voor deze vragen en om te wegen en meten.
- Uw kind heeft zelf geen direct voordeel als hij/zij aan het onderzoek meedoet.
  Als uw kind mee doet helpt u en uw kind de kennis over hoe we zieke kinderen beter en veiliger kunnen voeden.

9. Wat gebeurt er bij verzet van uw kind bij deelname aan het onderzoek?

Het behandelteam van de afdeling Intensive Care begeleiden u en uw kind gedurende het onderzoek zo goed mogelijk. Dit team bestaat onder meer uit gespecialiseerde artsen, onderzoekers en verpleegkundigen. Het behandelteam let goed op de belasting die deelname aan het onderzoek voor uw kind met zich mee brengt. Indien de belasting voor uw kind groter is dan verwacht, stoppen we met het onderzoek. We werken daarbij volgens de landelijke afspraken zoals die door de Nederlandse Vereniging voor Kindergeneeskunde(NVK) zijn vastgelegd ter bescherming van minderjarige onderzoeksdeelnemers. Voor meer informatie hierover verwijzen wij u naar de volgende website: www.ccmo.nl (wet- en regelgeving/gedragscode verzet: minderjarigen).

10. Wat gebeurt er wanneer u besluit uw kind niet te laten deelnemen aan dit onderzoek?

U beslist zelf of u uw kind wilt laten meedoen, dit is volledig vrijwillig. Het heeft geen enkel gevolg voor de behandeling of het ziekteproces van uw kind. Het is enkel bedoeld voor de vooruitgang van de geneeskunde. Als u niet wilt dat uw kind meedoet met het onderzoek hoeft u niets te tekenen, zonder uitleg of het geven van een reden.

Voor meer informatie verwijzen we u naar pagina 8 t/m 12 van de Algemene brochure.

11. Wat gebeurt er als het onderzoek is afgelopen?

- Er zitten geen risico's aan het tussentijds staken van het onderzoek.
- Het onderzoek eindigt op het moment dat de studie volgens het protocol is afgelopen, of op het moment dat u of uw kind het onderzoek wil staken. Ook kan het zijn dat de onderzoeker of het behandelteam het onderzoek stopt.

12. Is uw kind verzekerd wanneer hij/zij aan het onderzoek meedoet?

Voor iedereen die meedoet aan dit onderzoek is een verzekering afgesloten, deze verzekering is wettelijk verplicht. Meer informatie vindt u in de Algemene brochure op pagina 14.

In bijlage 2 vindt u de verzekerde bedragen, de uitzonderingen en de adresgegevens van de verzekeraar.

13. Wordt u geïnformeerd als er tussentijds voor u relevante informatie over de studie bekend wordt?

Het onderzoek zal zo nauwkeurig mogelijk volgens plan verlopen. Maar de situatie kan veranderen. Bijvoorbeeld door de reactie van het lichaam van uw kind, of door nieuwe informatie. Als dat zo is, bespreken we dat direct met u. U beslist dan zelf of u met het onderzoek wilt stoppen of doorgaan. Als de veiligheid of welbevinden van uw kind in gevaar is, stoppen we direct met het onderzoek.

14. Wat gebeurt er met de gegevens van uw kind?

De gegevens blijven geheim, krijgen een code en de naam van uw kind wordt weggelaten.

De gegevens worden bewaard gedurende 15 jaar. Dit mag alleen wanneer u daar toestemming voor geeft.

Lichaamsmateriaal, dit is bloed, urine, wangslijmvlies, haar en ontlasting, wordt gedurende het onderzoek met een code bewaard en na afloop van het onderzoek vernietigd.

De mensen die de gegevens kunnen zien zijn het onderzoeksteam, de toetsingscommissie, de veiligheidscommissie en de inspectie van de gezondheidszorg.

Voor meer informatie verwijzen we u naar pagina 12, 14 en 15 van de Algemene brochure.

15. Wordt de huisarts van uw kind geïnformeerd bij deelname?

Wij kunnen de huisarts (schriftelijk) laten weten dat uw kind meedoet aan het onderzoek. Dit is voor de eigen veiligheid van uw kind. U geeft hiervoor toestemming op het toestemmingsformulier.

16. Zijn er extra kosten/ is er een vergoeding wanneer u besluit uw kind aan dit onderzoek mee te laten doen?

Aan het onderzoek zijn voor u geen kosten verbonden. U krijgt ook geen vergoeding voor

deelname van uw kind aan het onderzoek

17. Welke medisch-ethische toetsingscommissie heeft dit onderzoek goedgekeurd?

De Centrale Commissie Mensgebonden Onderzoek (CCMO) heeft dit onderzoek goed gekeurd. Meer informatie over de goedkeuring en controle van het onderzoek vindt u in de Algemene brochure op pagina 13.

18. Wilt u verder nog iets weten?

Voor het verlenen van toestemming krijgt u bedenktijd. Voor het bedenken of u uw kind mee wil laten doen krijgt u bedenktijd. U kunt altijd iets vragen aan de behandelend arts van uw kind of aan het team van onderzoekers.

De onderzoekers

Als u tijdens of na het onderzoek nog vragen of opmerkingen heeft, kunt u contact op nemen met een van de onderzoekers. U kunt hen tijdens kantooruren bereiken via het centrale telefoonnummer **010-7040704**, u kunt daarna vragen naar de desbetreffende onderzoeker.

- Dorian Kerklaan, arts-onderzoeker Intensive Care, tel. 06-83214901
- Dr. S. Verbruggen, Kinderarts Intensive Care Tel. 06-24792312
- Dr. Koen Joosten, Kinderarts Intensive Care, zoemer 36918

Onafhankelijke arts

Indien u twijfelt over deelname of meer informatie wenst over het onderzoek kunt u een onafhankelijke arts raadplegen. Deze arts is niet zelf bij het onderzoek betrokken, maar wel deskundig op het gebied van dit onderzoek. De onafhankelijk arts voor dit onderzoek is:

Prof. Dr. I. Reiss, kinderarts in het Sophia Kinderziekenhuis / Erasmus MC

Telefoonnummer: 010-7040704, zoemer 36026

Klachten

Indien u niet tevreden bent over de gang van zaken tijdens het onderzoek van uw kind, dan kunt u terecht bij de onderzoekers, de behandelend kinderarts of de onafhankelijke klachtencommissie van het ziekenhuis: tel. 010-7033198.

19. Bijlagen

1. Algemene brochure medisch-wetenschappelijk onderzoek met mensen.
2. Verzekering

Bijlage 2. Verzekering

Voor de deelnemers aan dit onderzoek is een verzekering afgesloten. Deze verzekering dekt schade door letsel of dood die het gevolg is van deelname aan het onderzoek, en die zich gedurende de deelname aan het onderzoek openbaart, of binnen vier jaar na beëindiging van de deelname aan het onderzoek. De schade wordt geacht zich te hebben geopenbaard wanneer deze bij de verzekeraar is gemeld.

In geval van schade kunt u zich direct wenden tot de verzekeraar.

De verzekeraar van het onderzoek is:

Naam: Meeus Assurantiën BV

Adres: Postbus 3234, 4800 DE Breda

Telefoonnummer: 076-5313480

Contactpersoon: Mw. Mr. F.A.C. Bergervoet (gezondheidsjurist, afdeling Vrije Beroepen)

De verzekering biedt een maximum dekking van € 450.000 per proefpersoon en een maximumbedrag van € 3.500.000 voor het gehele onderzoek. Indien de opdrachtgever van dit onderzoek meerdere onderzoeken heeft lopen, geldt een maximumbedrag van

€ 5.000.000 euro per verzekeringsjaar voor álle onderzoeken.

De dekking van specifieke schades en kosten is verder tot bepaalde bedragen beperkt. Dit is opgenomen in het Besluit verplichte verzekering bij medisch-wetenschappelijk onderzoek met mensen. Informatie hierover kunt u vinden op de website van de Centrale Commissie Mensgebonden Onderzoek: www.ccmo.nl.

Voor deze verzekering gelden een aantal uitsluitingen. De verzekering dekt niet:

- schade waarvan op grond van de aard van het onderzoek zeker of nagenoeg zeker was dat deze zich zou voordoen;
- schade aan de gezondheid die ook zou zijn ontstaan indien u niet aan het onderzoek had deelgenomen;
- schade die het gevolg is van het niet of niet volledig nakomen van aanwijzingen of instructies;
- schade aan nakomelingen, als gevolg van een nadelige inwerking van het onderzoek op u of uw nakomeling;
- bij onderzoek naar bestaande behandelmethoden: schade die het gevolg is van één van deze behandelmethoden;
- bij onderzoek naar de behandeling van specifieke gezondheidsproblemen: schade die het gevolg is van het niet verbeteren of van het verslechteren van deze gezondheidsproblemen.

Toestemmingsformulier behorende bij het onderzoek:

"Vroege versus Late Infuusvoeding bij zieke kinderen"

- Ik ben gevraagd om toestemming te geven, zodat mijn kind meedoet aan dit medisch wetenschappelijke onderzoek:

Naam kind ______________________________ Geboortedatum __ / __ / __

dag maand jaar

- Ik heb de informatiebrief gelezen. Ik begrijp de informatie. Ik kon aanvullende vragen stellen. De vragen zijn naar tevredenheid beantwoord. Ik heb voldoende tijd gehad om te beslissen mijn kind meedoet.

- Ik weet dat meedoen helemaal vrijwillig is. Ik weet dat ik op ieder moment kan beslissen dat mijn kind toch niet meedoet. Daarvoor hoef ik geen reden te geven.

- Ik geef toestemming om de specialist(en), die mijn kind behandelt, te vertellen dat hij/zij meedoet aan dit onderzoek.

- Ik weet dat de mensen van het onderzoeksteam, de toestingscommissie, veiligheidscommissie en de inspectie van de gezondheidszorg de gegevens van mijn kind kunnen zien..

- Ik geef toestemming om de gegevens te gebruiken, voor de doelen die in de informatiebrief staan.

- Ik vind het goed dat mijn kind meedoet aan dit onderzoek.

- Ik geef wel/geen toestemming om mij/mijn kind na afloop van het onderzoek te benaderen voor vervolgonderzoek

- Ik geef wel/geen toestemming om hoofdhaar bij mijn kind af te knippen ten behoeve van wetenschappelijk onderzoek.

__________________________________ ____________

Naam ouder/ voogd 1 Handtekening Datum __ / __ / __

Dag maand jaar

__________________________________ ____________ Datum __ / __ / __

Naam ouder/ voogd 2 Handtekening Dag maand jaar

Ik verklaar hierbij dat ik bovengenoemde persoon/personen volledig heb geïnformeerd over het genoemde onderzoek.

Als er tijdens het onderzoek informatie bekend wordt die de toestemming van de ouder of voogd zou kunnen beïnvloeden, dan breng ik hem/haar daarvan tijdig op de hoogte.

__________________________________ ____________

Naam onderzoeker Handtekening Datum __ / __ / __
dag maand jaar

Functie:___________________________

Aanvullende informatie is gegeven door.

__________________________________ ____________

Naam onderzoeker Handtekening Datum __ / __ / __
dag maand jaar

Functie:___________________________

Summary of changes in protocol PEPaNIC

PEPaNIC protocol amendment 1, Date May 2, 2013

Addition of:

1. Stollery Children's Hospital Edmonton as recruiting site

2. Collecting buccal swabs as reflection of intestinal intolerance and inflammation

- Ad 1: introduction and rationale (page 10)

  One of the goals of the PEPaNIC study is to look into the tolerance for enteral nutrition (EN). In critically ill children EN is preferred because it is more physiological, presents fewer complications, leads to a better outcome and is less expensive than PN. However, EN often fails in this patient group due to impaired gastrointestinal (GI) motility, digestion and absorption following ischemia, inflammation or starvation leading to intolerance to EN. To improve future EN tolerance in critically ill children we have to gain insight in parameters of influence in this complex process.

  One of the paramaters is mucosal inflammation, which can be related to malabsorption, as seen in patients with inflammatory bowel disease (IBD). Intestinal epithelial cells play a role in initiating and regulating mucosal immune responses through the secretion of chemokines. Studies in patients with IBD showed that epithelial cells derived from the intestine produce significant amounts of pro and anti-inflammatory chemokines (CXCL-8, CXCL-9 and CXCL-10). Chemokine expression can be measured in buccal epithelium which can be used as a reflection of intestinal inflammation.

- Ad 2: objectives (page 13):
  To study the underlying physiological mechanisms of enteral nutrition (EN) intolerance of critically ill children and to identify markers of tolerance for EN to use in future intervention studies clinical signs of intolerance (gastric residual volume, diarrhoea, vomiting, abdominal swelling) will be recorded. Buccal swabs will be collected at several moments and will be stored for analysis (epithelial cell expression of chemokines).

- Ad 5.1: treatment of subjects - investigational treatment (page 21): *In* ***Edmonton*** *patients in all age groups receive continuous insulin infusion to target blood glucose levels <180 mg/dl*

- Ad 7.1.2: methods – secondary study parameters/endpoints(page 26):
  h. Enteral nutrition (EN) tolerance: proportion of patients with diarrhoea, high gastric residual volumes, vomiting and/or abdominal swelling and relation with buccal epithelial cell expression of chemokines (CXCL-8. CXCL-9 and CXCL-10)

- Ad 7.3: data collection following recruitment (page 33-34):

Buccal swabs will be collected at day of admission (day 1), day 2, 4 and 6, and possibly on day of discharge and will be immersed in RNA-later and stored at -80C. Buccal epithelial cell expression of chemokines (CXCL-8. CXCL-9 and CXCL-10) will be measured in by Taqman analysis.

- *In children > 10 kg 1,8 milliliter extra blood (EDTA and heparine) will be collected on 4 selected days*

Informed consents were adapted accordingly.

CCMO

CENTRALE
COMMISSIE
MENSGEBONDEN
ONDERZOEK

Erasmus MC –Sophia afdeling Intensive Care kindergeneeskunde
Tav Dr SCAT Verbruggen, kamer Sp 3140
Postbus 2060
3000 CB Rotterdam

12 juni 2013
uw kenmerk: n.v.t., ons kenmerk: CCMO13.0467/GK /cb/38772

Nader besluit NL38772.000.12

Geachte heer Verbruggen,

Hierbij zend ik u het nader besluit van de CCMO inzake het amendement bij het onderzoeksprotocol getiteld 'Paediatric Early versus late PArenteral Nutrition in Critical illness (PEPaNIC)' (NL38772.000.12).

De CCMO verleent haar goedkeuring aan genoemd amendement. Voor de overwegingen bij het besluit verwijs ik u naar het bijgevoegde oordeel.

Wij verzoeken u alle bij de uitvoering van het onderzoek betrokken partijen van het besluit op de hoogte te brengen. U kunt hiervoor gebruik maken van de digitale versie van het besluit zoals te vinden in ToetsingOnline, in het dossier van deze studie.

Ik hoop u hiermee naar behoren te hebben geïnformeerd.

Hoogachtend,

Namens de Centrale Commissie Mensgebonden Onderzoek
Prof dr GH Koëter, voorzitter

postadres:
Postbus 16302
2500 BH Den Haag
bezoekadres:
Rijnstraat 50
2515 VX Den Haag
tel: (070) 340 6700
ccmo@ccmo.nl
www.ccmo.nl

PEPaNIC protocol amendment 2, Date December, 17, 2013

Addition of:

1. Collecting hair samples for studying cortisol metabolism levels

2. collecting blood samples for studying epigenetic changes

- Ad 7.1.2: methods – secondary study parameters/endpoints(page 30):

  Impact of early versus late PN on the neuroendocrine axes: The metabolism of cortisol will be studied further by the relative levels of its metabolites excreted in urine (26) and by collecting hair samples for the long term cortisol levels.

- Ad 7.3: data collection following recruitment (page 33):

  Around 100 strands of hair from the posterior vertex of the scalp will be cut off as soon as possible after admission and this will be repeated after 3 weeks- 2 months if possible.

- Ad 7.3: data collection following recruitment (page 34):

  On day 3,5 and 7 a 'PAX-tube' (containing 2 milliliter blood) will be collected.

Informed consents were adapted accordingly.

Centrale
Commissie
Mensgebonden
Onderzoek

Postadres:
Postbus 16302
2500 BH Den Haag
Bezoekadres:
Rijnstraat 50
2515 XP Den Haag
t. 070 340 67 00
ccmo@ccmo.nl
www.ccmo.nl

Erasmus MC
afdeling IC kinderen
Dr SCAT Verbruggen
Postbus 2060
3000 CB Rotterdam

Ons kenmerk: CCMO14.0023/GK/cb/38772

Uw kenmerk: nvt

Datum: 10 januari 2014

Betreft: Nader besluit amendement NL38772

geachte heer Verbruggen,

Hierbij zend ik u het besluit van de CCMO inzake het amendement bij het onderzoek 'Pediatric Early versus late Parenteral Nutrition in Critical Illness', (NL38772.000.12).

De CCMO verleent goedkeuring aan genoemd amendement. Voor de overwegingen hierbij verwijs ik u naar het bijgevoegde besluit.

Wij verzoeken u alle bij de uitvoering van het onderzoek betrokken partijen van het besluit op de hoogte te brengen. U kunt hiervoor gebruikmaken van de digitale versie van het besluit dat in ToetsingOnline is te vinden in het dossier van deze studie.

Ik hoop u hiermee naar behoren te hebben geïnformeerd.

Met vriendelijke groet,
Namens de Centrale Commissie Mensgebonden Onderzoek

Prof dr GH Koëter, voorzitter

Final statistical analysis plan

NB: only one version of the SAP was created and therefore no summary of changes has been included

Background

The prevalence of undernutrition in children who are admitted to the Pediatric Intensive Care Unit (PICU) is up to 24%.[1] Undernutrition on admission is associated with increased mortality and morbidity such as infectious complications, longer need for mechanical ventilation and prolonged hospital stay.[2-4]

Hulst et al showed that the nutritional status of children admitted to the PICU declined from admission to discharge from the PICU and that these children showed catch-up growth within 6 months.[1] Both protein intake of at least 2.3 mg/kg/day during PICU stay and higher enteral caloric intake after PICU stay have been associated with an increase in weight-for-age (WFA) SD score in subgroups of children.[5,6] However, this association could not be found for duration of parenteral nutrition (PN).[6] Furthermore, it is unknown if deterioration of nutritional status during PICU stay independently impairs clinical outcome.

The PEPaNIC trial in 1440 critically ill children showed that withholding PN for one week resulted in fewer new infections and reduced the duration of PICU stay as compared with early initiation of PN.[7] These clinical benefits were even larger in children who were at high risk of undernutrition as reflected by a high score on the Screening Tool for Risk on Nutritional status and Growth (STRONGkids). However, the STRONGkids score has originally been developed for children admitted to the pediatric ward, not children admitted to the PICU. The effect of late initiation of PN in children who were undernourished upon admission has not yet been evaluated.

Aims and hypotheses

The aim of this secondary analysis is to investigate the clinical effect of late PN in children who were undernourished upon admission, and to investigate the prognostic value of change in weight during PICU stay and the effect of late PN hereon. Furthermore, we will analyze the impact of Late PN on the relation between weight change and clinical outcome in relation to undernourishment upon admission, if the number of patients in each group is sufficiently large.

We hypothesize that undernourished children who have been allocated to the late PN group will have less new infections and a shorter duration of PICU stay than those allocated to the early PN group. Furthermore, in line with previous findings, we expect that weight deterioration is associated with worse clinical outcome. We expect that there is no difference in weight change between children receiving early PN and those receiving late PN, and that the relation between weight change and clinical outcome is not affected by late PN.

Methods

Context

These hypotheses will be tested with data from the PEPaNIC trial.[7] A total of 1440 children from three large tertiary referral PICUs in three different countries (KU Leuven in Belgium, Erasmus MC in The Netherlands and Stollery Children's Hospital in Canada) were randomly assigned to late PN (Late PN; intervention) or early PN (Early PN; standard care). Initiation and incline of enteral nutrition and administration of trace elements, minerals and vitamins were identical in both groups. Patients assigned to the late PN group (n=717) received no PN at all during the first week of critical illness. Patients in the early PN group (n=723) received PN within 24 hours after admission to supplement insufficient enteral nutrition. After the first week,

PN was administered equally in both groups according to standard nutritional protocol if enteral nutrition was insufficient to meet nutritional goals.[7,8] The full study protocol and clinical outcomes have been reported in detail elsewhere.[7,8]

Data collection and definitions

Data on patient characteristics, outcome measurements and parameters for nutritional status were prospectively collected and registered in the study database. Since multiple measurements of weight and height were not performed for the Belgian and Canadian patients, we will exclude these patients for the analyses concerning change in weight during PICU stay.

The nutritional status will be defined based on WFA in children younger than one year and BMI-for-age (BFA) in children older than one year. Acute undernutrition is defined as WFA or BFA SD scores <-2. Country specific reference values for WFA and BFA will be used to calculate SD scores, if possible. If these are unavailable, WHO reference values will be used.

Outcomes

Primary outcomes are incidence of new infections and duration of PICU dependency, which will be reported as the crude number of PICU days and as the time to live discharge from the PICU, to account for mortality as a competing risk.

Secondary outcomes are duration of mechanical ventilation (and time to life weaning from mechanical ventilation), duration of hospital stay (and time to life discharge from the hospital), and the safety outcomes 7-day mortality (i.e. during the intervention period), 90-day mortality and hypoglycemia during the first week.

Statistical analyses

As the sample size of the present study depended on the number of undernourished children included in the PEPaNIC-trial, we plan to perform a retrospective power analysis based on observed differences for risk of new infection as primary endpoint, rather than performing an a priori sample size calculation.

Variables will be reported as percentages, means (±SD) if normally distributed or as medians (IQR) if not-normally distributed. Change in weight will be reported as change in WFA or BFA SD score. Proportions will be analyzed univariably using chi-square test, means with t-test and medians with Mann Whitney test. The time-to-event effect sizes will be estimated with use of Cox proportional-hazard analysis, with data censored at 90 days. To take into account death as a competing risk for outcomes related to duration of care, data for non-survivors will be censored at 91 days. These time-to-event outcomes will be analyzed adjusted for baseline risk factors treatment center, age, diagnosis group, STRONGkids category, PIM2 score and PELOD score. The multivariable analysis of the effect of the intervention on dichotomized outcomes, adjusted for baseline risk factors, will be performed using logistic regression. Analyses regarding change in weight will additionally be adjusted for malnourishment upon admission.

P-values ≤0.05 will be considered statistically significant. P-values for interaction were calculated with the use of multivariable logistic regression analyses and multivariable Cox proportional-hazard analyses with a threshold for significance of interaction set at P≤0.10.

References

1. Hulst J, Joosten K, Zimmermann L, et al. Undernutrition in critically ill children: from admission to 6 months after discharge. *Clin Nutr.* 2004;23(2):223-232.

2. Hecht C, Weber M, Grote V, et al. Disease associated undernutrition correlates with length of hospital stay in children. *Clin Nutr.* 2015;34(1):53-59.
3. de Souza Menezes F, Leite HP, Koch Nogueira PC. Undernutrition as an independent predictor of clinical outcome in critically ill children. *Nutrition.* 2012;28(3):267-270.
4. Bagri NK, Jose B, Shah SK, Bhutia TD, Kabra SK, Lodha R. Impact of Undernutrition on the Outcome of Critically Ill Children. *Indian J Pediatr.* 2015;82(7):601-605.
5. Bairdain S, Khan FA, Fisher J, et al. Nutritional outcomes in survivors of congenital diaphragmatic hernia (CDH)-factors associated with growth at one year. *J Pediatr Surg.* 2015;50(1):74-77.
6. Hong BJ, Moffett B, Payne W, Rich S, Ocampo EC, Petit CJ. Impact of postoperative nutrition on weight gain in infants with hypoplastic left heart syndrome. *J Thorac Cardiovasc Surg.* 2014;147(4):1319-1325.
7. Fivez T, Kerklaan D, Mesotten D, et al. Early versus Late Parenteral Nutrition in Critically Ill Children. *N Engl J Med.* 2016;374(12):1111-1122.
8. Fivez T, Kerklaan D, Verbruggen S, et al. Impact of withholding early parenteral nutrition completing enteral nutrition in pediatric critically ill patients (PEPaNIC trial): study protocol for a randomized controlled trial. *Trials.* 2015;16:202.
