# Supplementary material for: Outcomes of Delaying Parenteral Nutrition for 1 Week vs Initiation Within 24 Hours Among Undernourished Children in Pediatric Intensive Care: A Subanalysis of the PEPaNIC Randomized Clinical Trial
Source: JAMA Netw Open. 2018 Sep 14;1(5):e182668. doi: 10.1001/jamanetworkopen.2018.2668 (PMC6324499; doi:10.1001/jamanetworkopen.2018.2668)
Supplement: Supplement 2. — eMethods. Protocol for Scoring of Infections eTable 1. Energy and Macronutrient Administration in Undernourished Children for the First Seven Days in PICU eTable 2. Baseline Characteristics of Undernourished vs Well-Nourished Children eTable 3. Main Outcomes of Undernourished vs Well-Nourished Children eTable 4. Baseline Characteristics of Children Well-Nourished on Admission in the Early-PN and Late-PN Group eTable 5. Outcomes of Late-PN vs Early-PN in Well-Nourished Children eTable 6. Baseline Characteristics of Children Severely Undernourished on Admission in the Early-PN and Late-PN Group eReferences [file jamanetwopen-1-e182668-s002.pdf]

Supplementary Online Content

van Puffelen E, Hulst JM, Vanhorebeek I, et al. Outcomes of delaying parenteral nutrition for 1 week vs initiation within 24 hours among undernourished children in pediatric intensive care: a subanalysis of the PEPaNIC randomized clinical trial. *JAMA Netw Open*. 2018;1(5):e182668. doi:10.1001/jamanetworkopen.2018.2668

eMethods. Protocol for Scoring of Infections
eTable 1. Energy and Macronutrient Administration in Undernourished Children for the First Seven Days in PICU
eTable 2. Baseline Characteristics of Undernourished vs Well-Nourished Children
eTable 3. Main Outcomes of Undernourished vs Well-Nourished Children
eTable 4. Baseline Characteristics of Children Well-Nourished on Admission in the Early-PN and Late-PN Group
eTable 5. Outcomes of Late-PN vs Early PN in Well-Nourished Children
eTable 6. Baseline Characteristics of Children Severely Undernourished on Admission in the Early-PN and Late-PN Group
eReferences

This supplementary material has been provided by the authors to give readers additional information about their work.

© 2018 van Puffelen E et al. *JAMA Network Open*.

eMethods. Protocol for Scoring of Infections

1. Data export All patients receiving antimicrobial agents were identified by the data manager, who provided an export of all patient numbers with all the information on antimicrobial agents given as well as the duration of such treatment.

2. Identification of patients with infections The infectious disease specialists, who were blinded for treatment allocation, selected all patients receiving antimicrobial agents for more than 48h, after excluding all patients who received prophylaxis. Each patient who fulfilled the criteria for infection, as well as the type of infection, was identified as such based on thorough review of the medical record[1]. Patients for whom antimicrobials where initiated prior to PICU admission or within the first 48 hours of admission while the criteria for infection were fulfilled, were labelled as “having an infection upon admission”. When antimicrobial agents were initiated after randomization and beyond the first 48 hours in the PICU, and were given for more than 48 hours while the criteria for infection were fulfilled, the patient was labelled as “having a new infection”.[1,2]

© 2018 van Puffelen E et al. *JAMA Network Open*.

eTable 1. Energy and Macronutrient Administration in Undernourished Children for the First Seven Days in PICU

		N		Total dose		Enteral dose		Parenteral dose	
	**Day**	**Early-PN**	**Late-PN**	**Early-PN**	**Late-PN**	**Early-PN**	**Late-PN**	**Early-PN**	**Late-PN**
Energy	1	139	150	23.5 (13.3-33.4)	5.3 (3.7-8.4)	0.0 (0.0-0.0)	0.0 (0.0-0.0)	22.3 (13.3-30.9)	5.2 (3.6-7.5)
(kcal/kg)	2	127	129	52.8 (45.2-66.3)	11.6 (8.2-29.6)	0.0 (0.0-23.5)	0.0 (0.0-20.1)	48.8 (27.7-54.8)	8.4 (6.1-10.6)
	3	106	105	74.0 (55.2-90.7)	27.6 (12.0-58.9)	4.1 (0.0-43.4)	14.4 (0.0-49.6)	54.8 (20.2-73.2)	8.0 (4.5-10.8)
	4	98	85	82.3 (60.6-101.0)	35.9 (20.1-70.5)	14.8 (0.0-59.3)	21.7 (0.0-67.4)	45.5 (16.4-84.4)	6.4 (2.4-12.0)
	5	84	68	93.8 (59.0-107.5)	42.8 (22.1-85.7)	25.1 (0.0-77.2)	37.7 (6.3-85.4)	38.1 (6.3-85.7)	5.4 (1.4-10.7)
	6	70	56	92.1 (60.6-102.3)	55.6 (21.6-82.9)	23.5 (0.0-70.3)	49.3 (10.0-82.3)	35.9 (2.8-95.8)	5.1 (1.4-10.3)
	7	62	44	98.3 (73.6-105.6)	52.6 (21.1-97.5)	42.1 (3.6-91.2)	44.2 (0.0-88.9)	25.4 (1.9-90.3)	4.8 (0.6-12.3)
Glucose	1	139	150	4.9 (3.3-6.9)	1.3 (0.9-2.1)	0.0 (0.0-0.0)	0.0 (0.0-0.0)	4.6 (3.3-6.4)	1.3 (0.9-1.9)
(g/kg)	2	127	129	9.8 (6.9-11.6)	2.8 (2.0-4.6)	0.0 (0.0-2.2)	0.0 (0.0-2.1)	8.9 (4.6-10.5)	2.1 (1.5-2.6)
	3	106	105	10.0 (7.3-12.4)	4.6 (2.7-7.6)	0.0 (0.0-5.0)	1.3 (0.0-4.9)	7.2 (2.8-11.3)	2.0 (1.1-2.7)
	4	98	85	10.0 (7.6-13.6)	5.6 (3.0-8.8)	1.2 (0.0-6.5)	2.5 (0.0-7.4)	5.8 (1.7-12.2)	1.6 (0.6-3.0)
	5	84	68	11.5 (7.6-14.8)	6.3 (3.8-9.9)	2.5 (0.0-8.0)	3.8 (0.6-9.5)	5.0 (1.1-11.8)	1.4 (0.4-2.7)
	6	70	56	11.5 (7.7-14.0)	5.9 (3.8-9.4)	2.1 (0.0-7.8)	4.4 (0.7-8.8)	4.9 (0.7-13.2)	1.3 (0.4-2.4)
	7	62	44	11.8 (8.9-14.6)	6.5 (3.6-10.8)	5.2 (0.2-9.4)	4.7 (0.0-9.4)	3.5 (0.5-11.9)	1.2 (0.1-2.9)
Amino acids	1	139	150	0.8 (0.0-1.4)	0.0 (0.0-0.0)	0.0 (0.0-0.0)	0.0 (0.0-0.0)	0.8 (0.0-1.4)	0.0 (0.0-0.0)
(g/kg)	2	127	129	2.1 (1.5-2.4)	0.0 (0.0-0.5)	0.0 (0.0-0.5)	0.0 (0.0-0.5)	2.0 (0.7-2.3)	0.0 (0.0-0.0)
	3	106	105	2.3 (1.7-2.6)	0.4 (0.0-1.3)	0.0 (0.0-1.1)	0.2 (0.0-1.2)	1.8 (0.2-2.4)	0.0 (0.0-0.0)
	4	98	85	2.1 (1.5-2.4)	0.7 (0.0-1.7)	0.3 (0.0-1.5)	0.7 (0.0-1.6)	1.1 (0.1-2.0)	0.0 (0.0-0.0)
	5	84	68	2.1 (1.8-2.5)	0.9 (0.1-2.0)	0.5 (0.0-1.9)	0.9 (0.1-2.0)	1.2 (0.0-2.0)	0.0 (0.0-0.0)
	6	70	56	2.0 (1.7-2.5)	1.1 (0.2-2.0)	0.5 (0.0-1.7)	1.1 (0.1-1.9)	1.3 (0.0-2.0)	0.0 (0.0-0.0)
	7	62	44	2.0 (1.8-2.6)	1.3 (0.2-2.2)	1.0 (0.0-2.1)	1.1 (0.0-2.1)	0.8 (0.0-2.0)	0.0 (0.0-0.0)

© 2018 van Puffelen E et al. *JAMA Network Open*.

Lipid	1	139	150	0.0 (0.0-0.0)	0.0 (0.0-0.0)	0.0 (0.0-0.0)	0.0 (0.0-0.0)	0.0 (0.0-0.0)	0.0 (0.0-0.0)
(g/kg)	2	127	129	0.6 (0.2-1.8)	0.0 (0.0-1.1)	0.0 (0.0-1.1)	0.0 (0.0-1.0)	0.2 (0.0-0.5)	0.0 (0.0-0.0)
	3	106	105	2.3 (1.6-3.5)	0.4 (0.0-2.4)	0.0 (0.0-2.1)	0.4 (0.0-2.2)	1.5 (0.2-1.9)	0.0 (0.0-0.0)
	4	98	85	3.1 (2.0-4.0)	1.1 (0.0-3.5)	0.5 (0.0-3.0)	0.9 (0.0-3.5)	1.6 (0.1-2.8)	0.0 (0.0-0.0)
	5	84	68	3.4 (2.0-4.5)	1.6 (0.2-4.1)	1.3 (0.0-4.0)	1.5 (0.2-4.1)	1.3 (0.0-2.4)	0.0 (0.0-0.0)
	6	70	56	3.1 (2.0-4.3)	2.0 (0.3-4.2)	0.8 (0.0-3.3)	2.0 (0.3-4.2)	1.2 (0.0-2.8)	0.0 (0.0-0.0)
	7	62	44	3.3 (2.2-5.1)	2.5 (0.4-4.8)	1.7 (0.1-4.5)	2.2 (0.0-4.8)	0.8 (0.0-2.9)	0.0 (0.0-0.0)

Data represent medians and interquartile ranges. All total doses (except lipids administered on day 1) and parenteral doses of energy, glucose, amino acids, and lipids were significantly different for patients in the Early-PN and Late-PN groups, whereas no significant differences were observed for enteral doses. N indicates the number of patients still in PICU on the respective days. PICU = Pediatric Intensive Care Unit; PN = parenteral nutrition

© 2018 van Puffelen E et al. *JAMA Network Open*.

eTable 2. Baseline Characteristics of Undernourished vs Well-Nourished Children

**Baseline characteristics**	**Undernourished**[a] (n=289)	**Well-nourished** (n=1110)	**P-value**
Male – no. (%)	173 (59.9)	634 (57.1)	0.40
Age at randomization - median years (IQR)	0.44 (0.22;2.82)	1.78 (0.24;6.74)	<0.001
High STRONGkids category – no (%)	58 (20.1)	91 (8.2)	<0.001
Weight Z-score – median (IQR)[b]	-2.98 (-3.71;-2.43)	-0.15 (-0.95;0.66)	<0.001
PELOD score – median (IQR)	21 (11;32)	21 (11;31)	0.36
PIM2 score – mean (SD)	-2.47 (1.61)	-2.53 (1.74)	0.56
Risk of mortality (%) – median (IQR)	6.4 (2.6-17.0)	5.7 (2.5-17.3)	0.28
Diagnostic group – no (%)			<0.001
Surgical			
Abdominal	18 (6.2)	92 (8.3)	
Burns	0 (0.0)	7 (0.6)	
Cardiac	124 (42.9)	423 (38.1)	
Neurosurgery	12 (4.2)	101 (9.1)	
Thoracic	5 (1.7)	55 (5.0)	
Transplant	2 (0.7)	22 (2.0)	
Trauma/orthopedic	16 (5.5)	36 (3.2)	
Other	6 (2.1)	42 (3.8)	
Medical			
Cardiac	12 (4.2)	48 (4.3)	
Gastro-intestinal/hepatic	2 (0.7)	4 (0.4)	
Hematologic/oncologic	2 (0.7)	13 (1.2)	
Neurologic	20 (6.9)	76 (6.8)	
Renal	0 (0.0)	2 (0.2)	
Respiratory	57 (19.7)	123 (11.1)	
Other	13 (4.5)	66 (5.9)	
Mechanical ventilation upon PICU admission – no. (%)	251 (86.9)	980 (88.3)	0.50

© 2018 van Puffelen E et al. *JAMA Network Open*.

Inotrope or vasopressor medication upon PICU admission – no. (%)	126 (43.6)	455 (41.0)	0.42
Mechanical hemodynamic support upon PICU admission – no. (%)	3 (1.0)	39 (3.5)	0.03

[a]Acutely undernourished defined as weight-for-age Z-score <-2 if <1 year, or BMI-for-age Z-score <-2 if ≥1 year[3,4];
[b]<1 year: weight-for-age Z-score, ≥1 year: BMI-for-age Z-score[3,4]; [c]based on PIM2 score =(exp (PIM2)/(1+exp (PIM2)))*100%
PICU = pediatric intensive care unit; STRONGkids = Screening Tool for Risk on Nutritional Status and Growth, range from 0 to 5, with a score of 0 indicating low risk of malnutrition, a score of 1 to 3 indicating medium risk, and a score of 4 to 5 indicating high risk; PELOD = Pediatric Logistic Organ Dysfunction, range from 0 to 71, with higher scores indicating more severe illness; PIM2 = Pediatric Index of Mortality 2, with higher scores indicating a higher risk of mortality.

© 2018 van Puffelen E et al. *JAMA Network Open*.

eTable 3. Main Outcomes of Undernourished vs Well-Nourished Children

**Main outcomes**	**Undernourished**[a] (n=289)	**Well-nourished** (n=1110)	**P-value**	**Adjusted OR or HR (95% CI)**[b]	**P-value**[b]
Primary endpoints					
New infections – No. (%)	48 (16.6)	161 (14.5)	0.37	1.23 (0.84-1.79)[c]	0.29
Duration of PICU stay — median days (IQR)	5 (2-9)	3 (2-8)	<0.001	0.86 (0.75-0.99)	0.03
Secondary safety endpoints					
Death during first week – no. (%)	2 (0.7)	33 (3.0)	0.03	0.10 (0.01-0.80)[c]	0.03
Death during PICU stay – no. (%)	10 (3.5)	53 (4.8)	0.34	0.82 (0.35-1.89)[c]	0.63
Death during hospital stay – no. (%)	16 (5.5)	65 (5.9)	0.84	1.14 (0.59-2.23)[c]	0.70
90-day mortality – no. (%)	17 (5.9)	65 (5.9)	0.99	1.29 (0.67-2.48)[c]	0.45
Hypoglycemia (blood glucose <40 mg/dl) during first week after randomization – no. (%)	32 (11.1)	67 (6.0)	0.003	1.65 (1.01-2.70)[c]	0.05
Secondary efficacy endpoints					
Duration of mechanical ventilatory support — median days (IQR)	3 (2-6)	2 (1-5)	<0.001	0.90 (0.79-1.03)	0.13
Duration of hospital stay — median days (IQR)	12 (7-26.5)	10 (6-22)	0.003	0.83 (0.73-0.96)	0.01

[a]Acutely undernourished defined as weight-for-age Z-score <-2 if <1 year, or BMI-for-age Z-score <-2 if ≥1 year[3,4]; [b]Odds ratio (OR) or Hazard ratio (HR), adjusted for randomization group, center, age, diagnosis group, PELOD score, PIM2 score, and STRONGkids category, with corresponding 95% Confidence Interval (CI). [c]These values are adjusted Odds Ratios, the other values are adjusted Hazard Ratios.

PICU = pediatric intensive care unit; STRONGkids = Screening Tool for Risk on Nutritional Status and Growth, range from 0 to 5, with a score of 0 indicating low risk of malnutrition, a score of 1 to 3 indicating medium risk, and a score of 4 to 5 indicating high risk; PELOD = Pediatric Logistic Organ Dysfunction, range from 0 to 71, with higher scores indicating more severe illness; PIM2 = Pediatric Index of Mortality 2, with higher scores indicating a higher risk of mortality.

© 2018 van Puffelen E et al. *JAMA Network Open*.

eTable 4. Baseline Characteristics of Children Well-Nourished on Admission in the Early-PN and Late-PN Group

**Characteristic**	**Early-PN** (n=565)	**Late-PN** (n=545)	**P-value**
Male – no. (%)	315 (55.8)	319 (58.5)	0.35
Age at randomization - median years (IQR)	1.81 (0.28-6.32)	1.77 (0.19-7.32)	0.77
High STRONGkids category – no. (%)	51 (9.0)	40 (7.3)	0.31
Weight Z-score – mean (SD)[a]	-0.07 (1.13)	-0.01 (1.37)	0.44
PELOD score – median (IQR)	21 (11-31)	21 (11-31)	0.98
PIM2 score – mean (SD)	-2.49 (1.75)	-2.58 (1.73)	0.37
Risk of Mortality (%) – median (IQR)[c]	5.8 (2.4-18.8)	5.5 (2.5-16.2)	0.51
Diagnostic group			0.83
Surgical			
Abdominal– no. (%)	45 (8.0)	47 (8.6)	
Burns– no. (%)	3 (0.5)	4 (0.7)	
Cardiac– no. (%)	221 (39.1)	202 (37.1)	
Neurologic– no. (%)	56 (9.9)	45 (8.3)	
Thoracic– no. (%)	30 (5.3)	25 (4.6)	
Transplant– no. (%)	7 (1.2)	15 (2.8)	
Trauma/orthopedic– no. (%)	19 (3.4)	17 (3.1)	
Other– no. (%)	16 (2.8)	26 (4.8)	
Medical			
Cardiac– no. (%)	23 (4.1)	25 (4.6)	
Gastro-intestinal/hepatic– no. (%)	2 (0.4)	2 (0.4)	
Hematologic/oncologic– no. (%)	7 (1.2)	6 (1.1)	
Neurologic– no. (%)	35 (6.2)	41 (7.5)	
Renal– no. (%)	1 (0.2)	1 (0.2)	
Respiratory– no. (%)	65 (11.5)	58 (10.6)	
Other– no. (%)	35 (6.2)	31 (5.7)	
Syndrome or genetic abnormality			0.24
No– no. (%)	479 (84.8)	480 (88.2)	
Yes– no. (%)	63 (11.2)	46 (8.5)	

© 2018 van Puffelen E et al. *JAMA Network Open*.

Suspected– no. (%)	23 (4.1)	18 (3.3)	
Mechanical ventilatory support upon PICU admission – no. (%)	501 (88.7)	479 (87.9)	0.69
Inotrope or vasopressor medication upon PICU admission – no (%)	234 (41.4)	221 (40.6)	0.77
Mechanical hemodynamic support upon PICU admission – no. (%)	17 (3.0)	22 (4.0)	0.35

[a] <1 year: weight-for-age Z-score, ≥1 year: BMI-for-age Z-score[3,4]; [b]PN = parenteral nutrition; STRONGkids = Screening Tool for Risk on Nutritional Status and Growth, range from 0 to 5, with a score of 0 indicating low risk of malnutrition, a score of 1 to 3 indicating medium risk, and a score of 4 to 5 indicating high risk [5]; PELOD = Pediatric Logistic Organ Dysfunction, range from 0 to 71, with higher scores indicating more severe illness; PIM2 = Pediatric Index of Mortality 2, with higher scores indicating a higher risk of mortality

© 2018 van Puffelen E et al. *JAMA Network Open*.

eTable 5. Outcomes of Late-PN vs Early PN in Well-Nourished Children

[a] Odds ratio (OR) or Hazard ratio (HR), adjusted for baseline risk factors center, age, diagnosis group, PELOD score, PIM2 score, and

**Outcomes**	**Early-PN** (n=565)	**Late-PN** (n=545)	**P-value**	**Adjusted OR or HR (95% CI)[a]**	**P-value[a]**
Primary endpoints					
New infections – no. (%)	102 (18.1)	59 (10.8)	0.001	0.53 (0.37-0.77)[b]	0.001
Duration of PICU stay — median days (IQR)	4 (2-8)	3 (2-7)	0.03	1.16 (1.03-1.31)	0.02
Secondary safety endpoints					
Death during first week – no. (%)	19 (3.4)	14 (2.6)	0.44	0.55 (0.22-1.40)[b]	0.21
Death during PICU stay – no. (%)	30 (5.3)	23 (4.2)	0.40	0.69 (0.34-1.37)[b]	0.29
Death during hospital stay – no. (%)	38 (6.7)	27 (5.0)	0.21	0.62 (0.34-1.14)[b]	0.12
90-day mortality – no. (%)	39 (6.9)	26 (4.8)	0.13	0.56 (0.30-1.05)[b]	0.07
Hypoglycemia (blood glucose <40 mg/dl) during first week after randomization – no. (%)	22 (3.9)	45 (8.3)	0.002	3.19 (1.77-5.73)[b]	<0.001
Weight Z-score deterioration – no. (%)	107 (59.1)	108 (57.8)	0.79	0.96 (0.63-1.47)[b]	0.85
Secondary efficacy endpoints					
Duration of mechanical ventilatory support – median days (IQR)	2 (1-6)	2 (1-4)	0.07	0.11 (0.98-1.25)	0.11
Duration of hospital stay – median days (IQR)	11 (6-22)	10-6-21)	0.09	1.12 (0.99-1.26)	0.08

STRONGkids category, with corresponding 95% Confidence Interval (CI). [b]These values are adjusted Odds Ratios, the other values are adjusted Hazard Ratios.

PN = parenteral nutrition; PICU = pediatric intensive care unit; STRONGkids = Screening Tool for Risk on Nutritional Status and Growth, range from 0 to 5, with a score of 0 indicating low risk of malnutrition, a score of 1 to 3 indicating medium risk, and a score of 4 to 5 indicating high risk; PELOD = Pediatric Logistic Organ Dysfunction, range from 0 to 71, with higher scores indicating more severe illness; PIM2 = Pediatric Index of Mortality 2, with higher scores indicating a higher risk of mortality.

© 2018 van Puffelen E et al. *JAMA Network Open*.

eTable 6. Baseline Characteristics of Children Severely Undernourished on Admission in the Early-PN and Late-PN Group

**Characteristic**	**Early-PN** (n=61)	**Late-PN** (n=76)	**P-value**
Male – no. (%)	43 (70.5)	52 (68.4)	0.79
Age at randomization - median years (IQR)	0.37 (0.21-0.63)	0.40 (0.20-2.52)	0.34
High STRONGkids category – no. (%)	15 (24.6)	17 (22.4)	0.76
Weight Z-score – mean (SD)[a]	-4.19 (0.94)	-4.17 (1.13)	0.90
PELOD score – median (IQR)	14 (6.5-32)	21.5 (12-31)	0.72
PIM2 score – mean (SD)	-2.79 (1.47)	-2.47 (2.03)	0.30
Risk of Mortality (%) – median (IQR)[c]	5.6 (2.0-20.3)	5.7 (2.5-16.6)	0.62
Diagnostic group			0.51
Surgical			
Abdominal– no. (%)	4 (6.6)	8 (10.5)	
Burns– no. (%)	0 (0)	0 (0)	
Cardiac– no. (%)	27 (44.3)	30 (39.5)	
Neurologic– no. (%)	1 (1.6)	4 (5.3)	
Thoracic– no. (%)	1 (1.6)	0 (0)	
Transplant– no. (%)	0 (0)	0 (0)	
Trauma/orthopedic– no. (%)	3 (4.9)	5 (6.6)	
Other– no. (%)	3 (4,1)	1 (1.3)	
Medical			
Cardiac– no. (%)	2 (3.3)	2 (2,6)	
Gastro-intestinal/hepatic– no. (%)	0 (0)	1 (1.3)	
Hematologic/oncologic– no. (%)	0 (0)	0 (0)	
Neurologic– no. (%)	5 (8.2)	5 (6.6)	
Renal– no. (%)	0 (0)	0 (0)	
Respiratory– no. (%)	15 (24.6)	16 (21.1)	
Other– no. (%)	0 (0)	4 (5.3)	
Syndrome or genetic abnormality			0.18
No– no. (%)	42 (68.9)	42 (55.3)	
Yes– no. (%)	17 (27.9)	27 (35.5)	

© 2018 van Puffelen E et al. *JAMA Network Open*.

Suspected– no. (%)	2 (3.3)	7 (9.2)	
Mechanical ventilatory support upon PICU admission – no. (%)	53 (86.9)	64 (84.2)	0.66
Inotrope or vasopressor medication upon PICU admission – no (%)	26 (42.6)	32 (42.1)	0.95
Mechanical hemodynamic support upon PICU admission – no. (%)	0 (0)	1 (1.3)	0.37

[a] <1 year: weight-for-age Z-score, ≥1 year: BMI-for-age Z-score[3,4]; [b]PN = parenteral nutrition; STRONGkids = Screening Tool for Risk on Nutritional Status and Growth, range from 0 to 5, with a score of 0 indicating low risk of malnutrition, a score of 1 to 3 indicating medium risk, and a score of 4 to 5 indicating high risk [5]; PELOD = Pediatric Logistic Organ Dysfunction, range from 0 to 71, with higher scores indicating more severe illness; PIM2 = Pediatric Index of Mortality 2, with higher scores indicating a higher risk of mortality

© 2018 van Puffelen E et al. *JAMA Network Open*.

eReferences

1. Horan TC, Andrus M, Dudeck MA. CDC/NHSN surveillance definition of health care-associated infection and criteria for specific types of infections in the acute care setting. *Am J Infect Control.* 2008;36(5):309-332.
2. Fivez T, Kerklaan D, Mesotten D, et al. Early versus Late Parenteral Nutrition in Critically Ill Children. *N Engl J Med.* 2016;374(12):1111-1122.
3. WHO Multicentre Growth Reference Study Group. WHO child growth standards: length/height-for-age, weight-for-age, weight-for-length, weight-for-height and body mass index-for-age: methods and development. Geneva: World Health Organization; 2006.
4. de Onis M, Onyango AW, Borghi E, Siyam A, Nishida C, Siekmann J. Development of a WHO growth reference for school-aged children and adolescents. *Bull World Health Organ.* 2007;85(9):660-667.
5. Hulst JM, Zwart H, Hop WC, Joosten KF. Dutch national survey to test the STRONGkids nutritional risk screening tool in hospitalized children. *Clin Nutr.* 2010;29(1):106-111.

© 2018 van Puffelen E et al. *JAMA Network Open*.
